# RNA-Seq and secondary metabolite analyses reveal a putative defence-transcriptome in Norway spruce (*Picea abies*) against needle bladder rust (*Chrysomyxa rhododendri*) infection

**DOI:** 10.1186/s12864-020-6587-z

**Published:** 2020-05-01

**Authors:** Carlos Trujillo-Moya, Andrea Ganthaler, Wolfgang Stöggl, Ilse Kranner, Silvio Schüler, Reinhard Ertl, Sarah Schlosser, Jan-Peter George, Stefan Mayr

**Affiliations:** 1 Federal Research and Training Centre for Forests, Landscape and Natural Hazards (BFW)-Department of Forest Genetics, Seckendorff-Gudent-Weg 8, 1131 Vienna, Austria; 2 Department of Botany, University of Innsbruck, Sternwartestraße 15, 6020 Innsbruck, Austria; 3 Federal Research and Training Centre for Forests, Landscape and Natural Hazards (BFW)- Department of Forest Growth & Silviculture, Seckendorff-Gudent-Weg 8, 1131 Vienna, Austria; 4 VetCore Facility for Research, University of Veterinary Medicine, Veterinärplatz 1, 1210 Vienna, Austria

**Keywords:** Conifer, Forest tree, Fungal infection, Host-pathogen-interaction, Phenolic compounds, PR proteins, RNA sequencing, Transcriptomics

## Abstract

**Background:**

Norway spruce trees in subalpine forests frequently face infections by the needle rust fungus *Chrysomyxa rhododendri*, which causes significant growth decline and increased mortality of young trees. Yet, it is unknown whether trees actively respond to fungal attack by activating molecular defence responses and/or respective gene expression.

**Results:**

Here, we report results from an infection experiment, in which the transcriptomes (via RNA-Seq analysis) and phenolic profiles (via UHPLC-MS) of control and infected trees were compared over a period of 39 days. Gene expression between infected and uninfected ramets significantly differed after 21 days of infection and revealed already known, but also novel candidate genes involved in spruce molecular defence against pathogens.

**Conclusions:**

Combined RNA-Seq and biochemical data suggest that Norway spruce response to infection by *C. rhododendri* is restricted locally and primarily activated between 9 and 21 days after infestation, involving a potential isolation of the fungus by a hypersensitive response (HR) associated with an activation of phenolic pathways. Identified key regulatory genes represent a solid basis for further specific analyses in spruce varieties with varying susceptibility, to better characterise resistant clones and to elucidate the resistance mechanism.

## Background

Biotic stress constitutes a major threat for forest trees, negatively affecting tree growth and survival, and compromising important ecological, economical, and social forest functions [46]. Altered climatic conditions due to climate change are expected to favour insect and microbial pathogen attacks [79], and to depreciate plant defence responses [43]. Some of the economically most relevant biotic agents are fungi, and numerous pathosystems of forest trees and either ascomycetes or basidiomycetes are known (e.g. [1, 20, 83]). Given that many of these pathosystems have co-evolved over very long time periods, evolutionary arm races between trees and fungi have resulted in significant phenotypic variation among trees growing on the same site [47]. As a consequence, some trees often remain healthy, whereas the vast majority develops symptoms of fungal infection. This phenotypic variation has been thoroughly studied at the DNA level by identifying the responsible major QTLs (Quantitative Trait Locus) (e.g. [24, 57]). In contrast, the defence transcriptome working upon infection is poorly understood. This is particularly the case in conifers, owing to the fact that genomic resources, such as annotated reference genomes and –transcriptomes, are rarely available [40]. Furthermore, studying gene expression in trees was so far restricted to the use of microarrays, which suffer from a number of technological weaknesses (e.g. cross-hybridization of related sequences) and only allowed for interrogating the expression pattern of already annotated genes [95]. Such draw backs have recently been overcome by completion of numerous reference genomes, such as for *P. abies* [74] and emerging next-generation-sequencing technologies (NGS), such as RNA-Seq which allows for the analysis of differential gene expression across the entire genome [48, 61, 89]. RNA-Seq has nowadays become a standard tool for investigating differences in gene expression under contrasting abiotic and biotic treatments, and thus may also be used to compare the expression of fungus-infected and non-infected individuals [91].

Among the various defence mechanisms against fungi described in plants, acquired or induced resistance (IR), although important [2], is not well understood and in long-living plants, such as trees, largely unexplored [22]. However, two specific molecular defence mechanisms within the IR spectrum, have been described in tree species (as reviewed in [22]): first, an inducible chemical defence that mainly comprises low molecular weight compounds (i.e. secondary metabolites) belonging to different biosynthetic pathways. Amongst them are terpenoids, phenolic compounds, and alkaloids, which are either working as phytoalexins [31] or phytoanticipins [86], depending on whether they are synthesized upon or already before an infection. In conifers, a large range of these metabolites is synthesized and accumulated in high concentrations in the needles [76, 92]. Second, an inducible defence system defined by a group of low molecular weight proteins (usually < 100 kDa) can be rapidly produced upon infection. These proteins are also called pathogenesis-related proteins (PR proteins) and are mainly involved in degrading and destroying the cell wall of the fungus, either directly or indirectly by enhancing the release of additional elicitors from the fungal cell wall (e.g. [9]).

Here, we present a study on a pathosystem, which comprises the economically important European conifer Norway spruce (*Picea abies*) and the rust fungus *Chrysomyxa rhododendri* [17]. The parasite undergoes a host shift between the main host rhododendron (*Rhododendron ferrugineum* or *R. hirsutum*) and Norway spruce and its occurrence is therefore restricted to higher elevation areas, where both hosts co-occur. Attacks have been reported since the end of the nineteenth century and continue to be regularly recorded by the forest health monitoring programmes carried out in different parts of the Alps [25, 96]. The infection of Norway spruce naturally occurs in spring, when freshly released basidiospores are wind-dispersed and arrive at current-year needles of the trees, where they quickly germinate and enter the young needles [8]. The infection causes a characteristic yellow needle discoloration during summer and severe needle loss in autumn [25], and repeated infections lead to significant reductions in timber yield. Infections also cause severe problems for rejuvenation at high elevation sites [25], which is critical considering the important protective function of mountain forests. Infected trees show several anatomical, morphological and physiological impairments, such as a decrease in chlorophyll content and net photosynthesis in affected needles, lower biomass production, and reduced radial and height growth [7, 25, 65]. Recent studies also indicated changes of the phenolic needle profile following infection and revealed phenolic concentrations of needles to correspond to their infection susceptibility [27, 28].

**Fig. 1:**
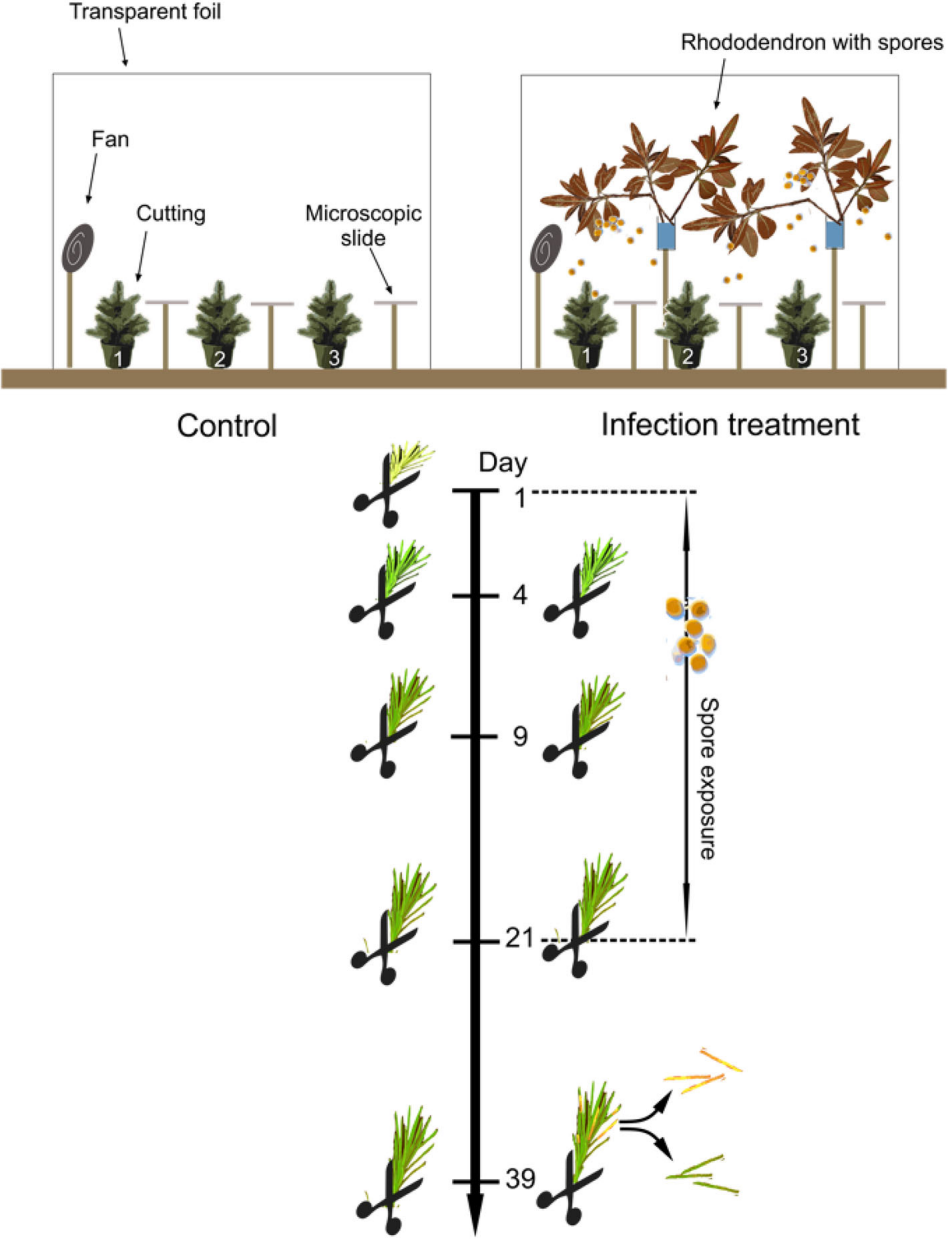
Study design. Design of plant treatment and sampling. Genetically identical cuttings were positioned for 39 days in a control and an infection tent (n = 3, respectively), the latter equipped for the first 21 days with rhododendron branches covered by mature *C. rhododendri* spore stocks. Fans improved spore distribution and microscopic slides collected spores for concentration monitoring. One current-year shoot per plant and time point was cut, the needles detached and stored at −80 ° C for RNA and phenolic analyses. Note that on day 39 infection symptoms were clearly visible on treated cuttings and needles were separated into healthy and symptomized ones

In the present study, we investigated the activation of defence mechanisms at both expression and metabolite level by combining next-generation-sequencing (RNA-Seq) with ultra high performance liquid chromatography-mass spectrometry (UHPLC-MS). In a greenhouse experiment, Norway spruce cuttings were infected with *C. rhododendri* and responses compared with uninfected controls over 39 days (for detailed study design see Fig. 1). Use of clonal ramets, derived from one tree, enabled us to focus on the analysis of changes in expression patterns, minimising the influence of genotypic variation. We hypothesized that *C. rhododendri* infection causes differential gene expression due to an inducible defence reaction. Differences in gene expression were expected to coincide with specific changes in concentration of phenolic compounds. A comparison of infected symptomatic needles and non-symptomatic needles on plants attacked by *C. rhododendri* should reveal if observed defence reactions are localized or systemic.

## Results

### cDNA sequencing and mapping of reads to the Norway spruce reference genome

A total of 19.9 million reads (median across samples) were obtained from cDNA sequencing of the libraries. The number of input reads (after quality control trimming) varied between 18.3 million and 22.5 million depending on treatment and time point (Table 1). The number of reads that uniquely aligned to a location in the reference genome varied between 6.6 million and 17.6 million, corresponding to 36 and 78% of uniquely aligned reads, respectively. Average input read length varied slightly between 72 and 73 nt. Inter-replicate correlation between the three technical replicates per treatment and time point combination was high in all cases, suggesting that cDNA sequencing produced reliable and robust results for downstream analyses (Additional file 1: Figure S1).

**Table 1:** Summary of RNA-sequencing and mapping results

Time point	Condition	Number of input reads	Number of uniquely mapped reads	Uniquely mapped reads %
T_1_	Baseline Control	22,035,322 (18,767,889-26,400,889)	17,130,560 (14,538,711-20,630,864)	77.69 (77.47–78.14)
T_4_	Control	22,496,254 (19,113,114-25,212,429)	17,574,219 (14,954,558-19,584,476)	78.14 (77.68–78.5)
T_9_	Control	19,503,128 (18,889,722-20,042,058)	15,364,519 (14,898,108-15,598,512)	78.79 (77.82–79.68)
T_21_	Control	22,109,409 (19,805,446-26,471,942)	17,150,393 (15,345,112-20,413,654)	77.62 (77.11–78.26)
T_39_	Control	20,954,099 (19,548,183-22,982,591)	16,150,647 (15,021,861-17,772,272)	77.06 (76.85–77.33)
T_4_	Infected	20,294,334 (17,827,443-22,137,544)	16,042,961 (14,297,556-17,475,634)	79.11 (78.19–80.20)
T_9_	Infected	20,571,206 (17,652,233-22,094,245)	16,243,524 (13,782,277-17,487,010)	78.91 (78.08–79.61)
T_21_	Infected	18,552,412 (18,380,964-18,769,955)	10,972,473 (9,567,906-12,175,200)	59.18 (50.97–65.79)
T_39_	Infected with symptoms	18,284,058 (17,804,501-18,763,615)	6,652,156 (6,600,580-6,703,731)	36.42 (35.18–37.65)
T_39_	Infected without symptoms	20,227,789 (19,812,720-20,642,858)	15,663,373 (15,240,145-16,086,601)	77.43 (76.92–77.93)

legend: Numbers in parentheses show the range between the three technical replicates

### Differential gene expression patterns among treatments and time points

**Fig. 2:**
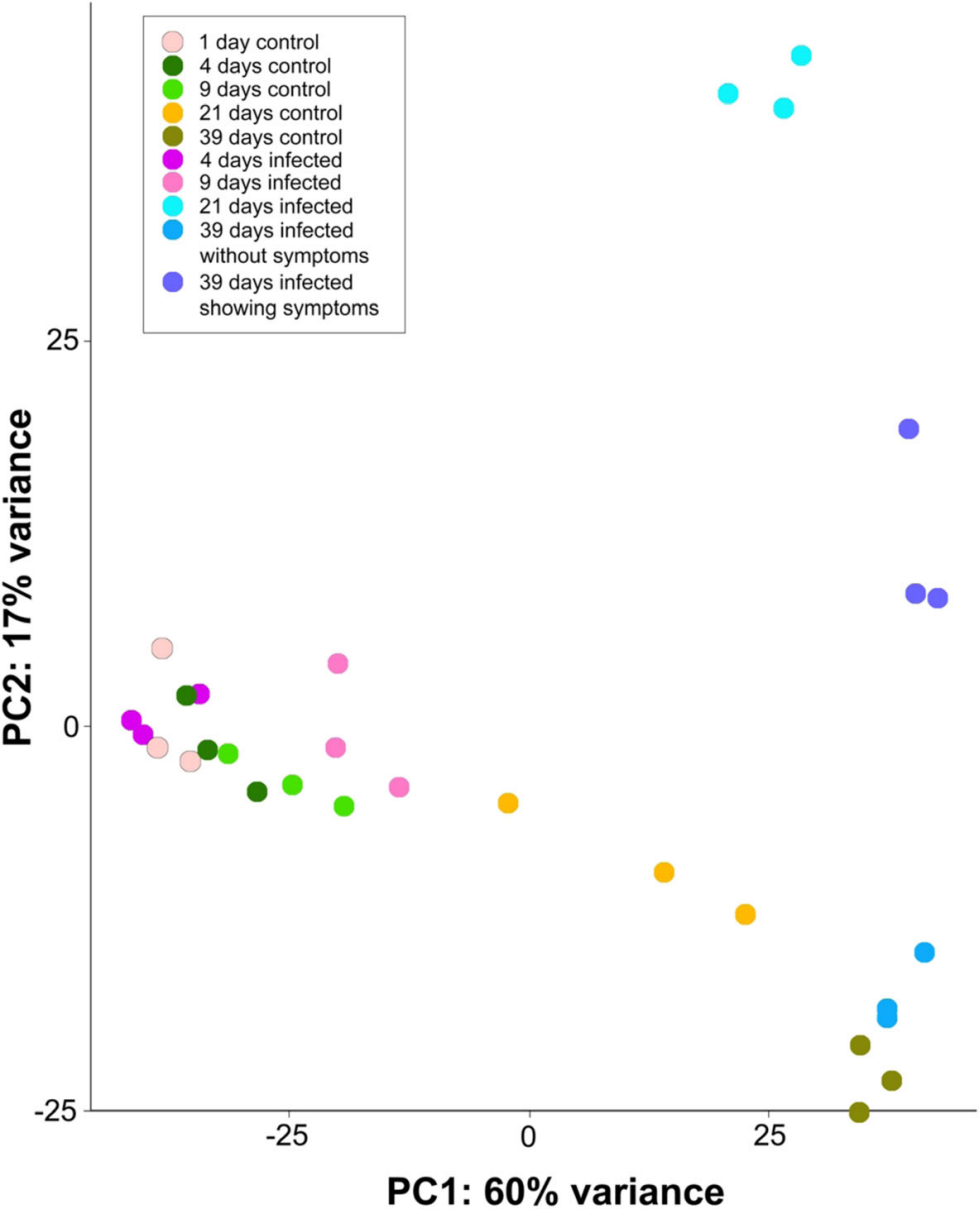
Principal component analysis (PCA) of differentially expressed transcripts. Note the high conformance within the three replicates and clear separation between controls and infection treatment at 21 and 39 dpi (symptomatic needles)

Principal component analysis of the overall differential gene expression profile revealed few differences between control and infected trees until 9 days post infection (dpi). This pattern changed drastically from 21 dpi on, when infected and non-infected trees showed significantly different gene expression profiles (Fig. 2). At that time, the first symptoms of infection were detectable, i.e., small blisters on the needle surface, visible under the microscope only. In contrast, at 39 dpi, infected trees showed the characteristic yellow needle discoloration (Additional file 2: Figure S2). Needles without symptoms sampled from the infected trees formed one cluster together with control samples, whereas needles with symptoms showed a different expression and were significantly separated along PC2.

### Global analysis of differentially expressed genes (DEGs)

**Fig. 3:**
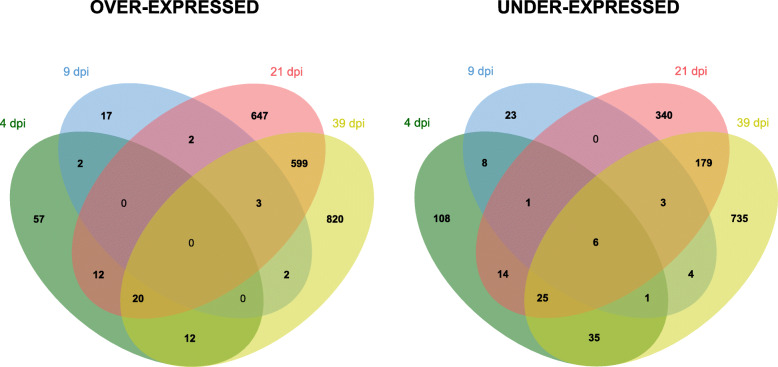
Venn’s diagrams of over- and under-expressed transcripts. Overlap between differentially expressed transcripts in *Picea abies* plants infected with *Chrysomyxa rhododendri* compared to control plants at the four time points after infestation (4 dpi, 9 dpi, 21 dpi, 39 dpi symptomatic needles)

**Fig. 4:**
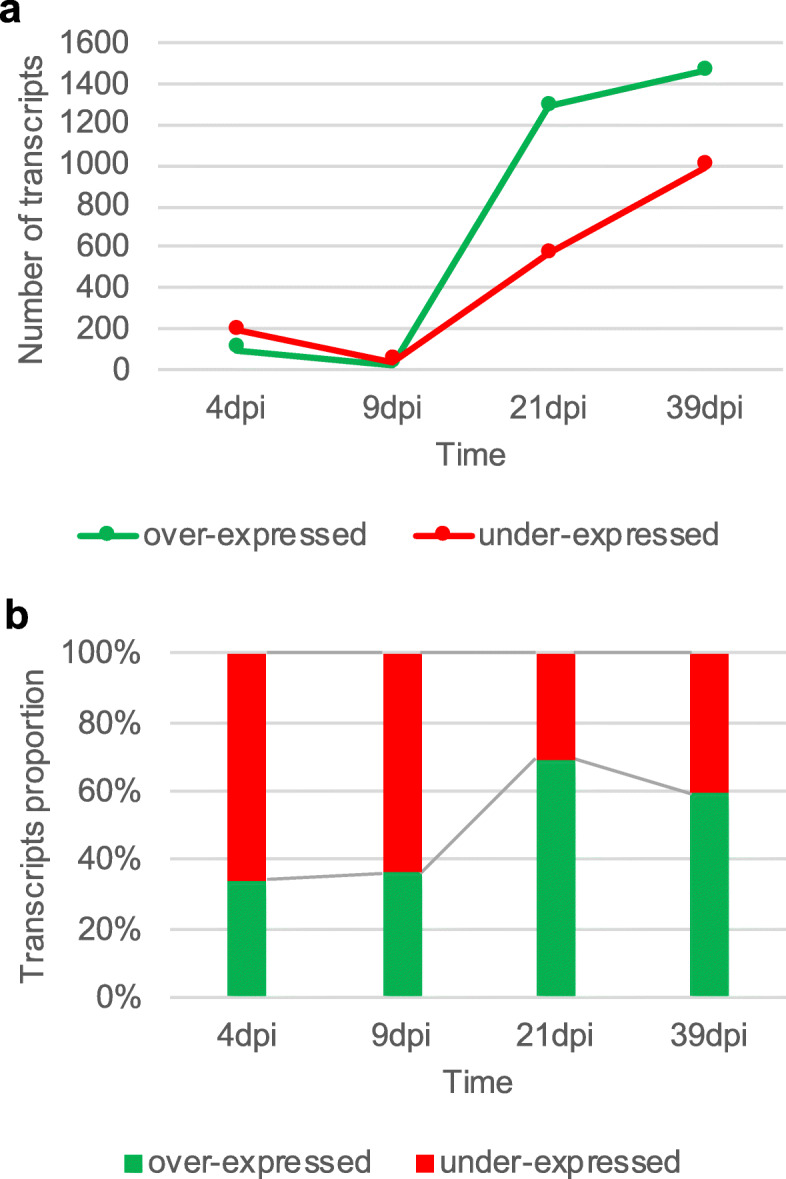
Number of differentially expressed transcripts in infested needles of *Picea abies.* Time course of (**a**) number and (**b**) proportions of over- and under-expressed transcripts between 4 and 39 dpi (symptomatic needles)

Overall 301, 72, 1851 and 2444 transcripts were differentially expressed in infected compared to control plants in needles at 4, 9, 21, and 39 dpi, respectively (Fig. 3 and Additional file 3: Table S1A-D). For this and subsequent analysis, 39 dpi symptomatic needles were considered as only 11 transcripts were differentially expressed in 39 dpi non-symptomatic needles compared to control plants (Additional file 3: Table S1E). 3320 transcripts were specifically over- and under-expressed at 21 dpi and 39 dpi but not at other time points (Fig. 3), whereby ~ 30% of over-expressed and ~ 15% of under-expressed transcripts were shared between both time points. During the infection period, the proportions of under- and over-expressed transcripts changed from ~ 35% over-expressed transcripts at 4 and 9 dpi, to ~ 60% at 21 and 39 dpi (Fig. 4).

### Gene ontology (GO) enrichment analysis

**Fig. 5:**
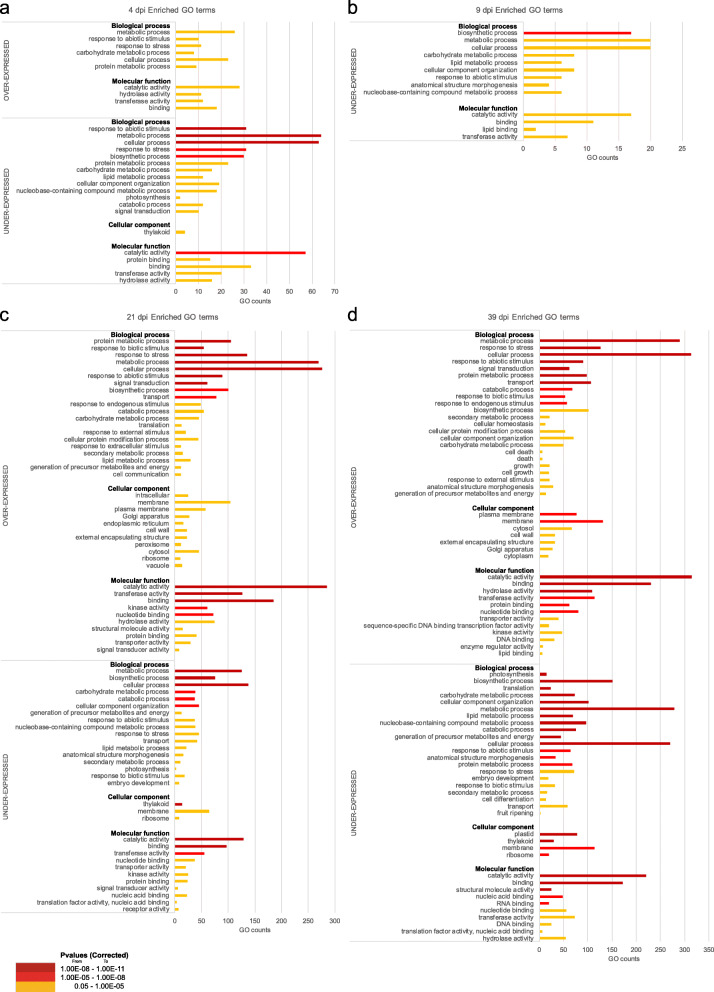
Gene ontology (GO) term enrichment analysis. GO terms overrepresented in over- or under-expressed transcripts of *Picea abies* needles infected by needle rust at (**a**) 4 dpi, (**b**) 9 dpi, (**c**) 21 dpi, and (d) 39 dpi (symptomatic needles). Terms ranked by the corrected *p*-value

Significantly enriched GO terms occurred in both, over- and under-expressed gene sets at all analyzed time points (Fig. 5). During the onset of infection (4 dpi) metabolic processes, response to abiotic stimulus and response to stress (category *biological processes*), as well as catalytic, hydrolase and transferase activity (*molecular functions*), whereas no *cellular component* term were enriched for over-expressed gene sets. Regarding the under-expressed gene set, similar enriched terms plus the *cellular component* thylakoid were found. At 9 dpi, low number of transcripts were differentially expressed and only under-expressed DEGs were significantly enriched. Among *biological processes*, the most enriched terms were biosynthetic, metabolic and cellular processes, whereas among *molecular functions*, the most enriched terms were catalytic activity, binding and lipid binding (Fig. 5), while no *cellular component* term was enriched. At 21 dpi, numerous enriched GO terms occurred both in over-expressed (41 terms) and under-expressed transcripts (31 terms). Among the over-expressed gene set, the most enriched terms were related to protein metabolic processes, response to biotic stimulus, response to stress, metabolic processes, cellular processes, response to abiotic stimulus, signal transduction and biosynthetic process (*biological processes*), intracellular aspects, membrane and plasma membrane (*cellular components*), catalytic activity, transferase activity, binding and kinase activity (*molecular function*). For under-expressed transcripts, the distribution of GO categories differed, and again changes of the *cellular component* thylakoid were indicated. Finally, at 39 dpi, 42 and 35 GO terms were enriched in the over- and under-expressed transcript sets, respectively, and observed patterns were very similar to 21 dpi. Most striking differences were observed in the *biological processes* category, with enriched GO terms in over-expressed transcripts related to cell death and growth, suggesting pronounced changes after 21 dpi (Fig. 5).

### Metabolic pathway analysis

With the emphasis on biochemical pathways, Kyoto Encyclopedia of Genes and Genomes (KEGG) database pathways were used as an alternative approach to categorize gene functions. On average, 40% of DEGs could be assigned to key enzymes involved in a total of 118 biological pathways, most of them at 21 and 39 dpi (Additional file 4: Table S2). Due to the complexity of the *P. abies* genome, several genes were assigned to the same key enzymes of pathways involved in metabolism, genetic information processing, environmental information processing, cellular processes and organismal systems (Table 2; Additional file 5: Table S3, Additional file 6: Table S4). Particularly notable is the over-expression of several key enzymes in the carbohydrate metabolism pathways, glycolysis/gluconeogenesis and starch and sucrose metabolism, and the under-expression of many key enzymes in the energy metabolism pathways, photosynthesis and carbon fixation. Translation, protein folding, sorting and degradation were also differentially regulated upon rust infection.

**Table 2:** KEGG pathways of differentially expressed genes by *Picea abies* infected by *Chrysomyxa rhododendri*

**METABOLISM**	4dpi		9dpi		21dpi		39dpi	
** Carbohydrate metabolism**	OVER	UNDER	OVER	UNDER	OVER	UNDER	OVER	UNDER
00010 Glycolysis / Gluconeogenesis		**1**		**1**	**5**	**5**	**13**	**11**
00020 Citrate cycle (TCA cycle)					**4**	**2**	**4**	**3**
00030 Pentose phosphate pathway		**1**	**1**	**1**	**2**	**3**	**5**	**7**
00040 Pentose and glucuronate interconversions		**1**			**1**	**2**	**4**	**5**
00051 Fructose and mannose metabolism		**1**		**1**	**3**	**2**	**6**	**4**
00052 Galactose metabolism		**1**			**4**	**3**	**5**	**1**
00053 Ascorbate and aldarate metabolism		**1**			**5**	**1**	**4**	**6**
00500 Starch and sucrose metabolism	**1**	**1**			**8**	**7**	**11**	**4**
00520 Amino sugar and nucleotide sugar metabolism		**1**			**6**	**4**	**9**	**4**
00620 Pyruvate metabolism					**3**	**2**	**5**	**7**
00630 Glyoxylate and dicarboxylate metabolism		**3**	**1**	**1**	**4**	**8**	**4**	**17**
00562 Inositol phosphate metabolism				**2**	**3**	**1**	**4**	**3**
** Energy metabolism**								
00190 Oxidative phosphorylation	**3**	**1**			**2**	**3**	**6**	**5**
00195 Photosynthesis	**1**	**2**		**1**		**8**		**27**
00196 Photosynthesis - antenna proteins	**1**					**2**		**7**
00710 Carbon fixation in photosynthetic organisms		**1**		**1**	**3**	**9**	**4**	**17**
00910 Nitrogen metabolism			**1**	**1**	**2**	**4**	**2**	**3**
00920 Sulfur metabolism				**2**	**4**		**3**	
** Lipid metabolism**								
00061 Fatty acid biosynthesis					**2**	**1**	**1**	**3**
00071 Fatty acid degradation	**2**				**4**	**2**	**6**	**5**
00073 Cutin, suberine and wax biosynthesis		**1**		**1**		**3**		**2**
00561 Glycerolipid metabolism					**4**	**2**	**5**	**1**
00564 Glycerophospholipid metabolism		**1**		**1**	**6**	**2**	**3**	
00565 Ether lipid metabolism				**1**	**3**		**2**	
00600 Sphingolipid metabolism					**3**		**5**	**2**
00592 alpha-Linolenic acid metabolism	**1**				**6**	**2**	**4**	**3**
01040 Biosynthesis of unsaturated fatty acids	**2**	**1**			**2**		**4**	**2**
** Nucleotide metabolism**								
00230 Purine metabolism				**1**	**3**	**3**	**5**	**4**
00240 Pyrimidine metabolism					**2**	**1**	**4**	**1**
** Amino acid metabolism**								
00250 Alanine, aspartate and glutamate metabolism		**1**			**3**	**2**	**4**	**4**
00260 Glycine, serine and threonine metabolism		**2**			**1**	**2**	**6**	**6**
00270 Cysteine and methionine metabolism	**1**			**1**	**7**	**3**	**9**	**4**
00280 Valine, leucine and isoleucine degradation					**2**	**1**	**3**	
00220 Arginine biosynthesis					**1**	**1**	**1**	**4**
00330 Arginine and proline metabolism					**6**	**1**	**5**	**3**
00350 Tyrosine metabolism					**3**	**2**	**4**	**5**
00360 Phenylalanine metabolism					**5**		**4**	**3**
00380 Tryptophan metabolism					**4**	**2**	**4**	**2**
00400 Phenylalanine, tyrosine and tryptophan biosynthesis		**1**			**2**	**2**	**2**	**3**
** Metabolism of other amino acids**								
00410 beta-Alanine metabolism					**5**	**3**	**6**	
00450 Selenocompound metabolism				**1**	**3**		**3**	**1**
00460 Cyanoamino acid metabolism			**1**		**3**	**1**	**2**	**3**
00480 Glutathione metabolism		**1**		**1**	**6**	**3**	**4**	**6**
** Metabolism of cofactors and vitamins**								
00730 Thiamine metabolism						**2**	**1**	**5**
00740 Riboflavin metabolism					**1**	**1**	**1**	**3**
00790 Folate biosynthesis				**1**	**3**	**3**	**1**	**1**
00670 One carbon pool by folate						**3**		**3**
00860 Porphyrin and chlorophyll metabolism	**1**			**2**		**5**	**2**	**13**
00130 Ubiquinone and other terpenoid-quinone biosynthesis					**4**		**3**	**2**
** Metabolism of terpenoids and polyketides**								
00900 Terpenoid backbone biosynthesis				**1**	**6**	**3**	**5**	**4**
00904 Diterpenoid biosynthesis					**2**	**1**	**1**	**3**
00906 Carotenoid biosynthesis		**2**			**1**	**3**	**1**	**7**
** Biosynthesis of other secondary metabolites**								
00940 Phenylpropanoid biosynthesis	**1**				**9**		**8**	**4**
00941 Flavonoid biosynthesis		**3**			**5**		**4**	**3**
**GENETIC INFORMATION PROCESSING**								
**Transcription**								
03040 Spliceosome	**2**	**1**			**1**	**1**	**5**	**4**
** Translation**								
03010 Ribosome	**2**	**1**		**1**	**19**	**7**	**7**	**31**
00970 Aminoacyl-tRNA biosynthesis		**1**				**3**	**1**	**7**
03013 RNA transport							**4**	**5**
03015 mRNA surveillance pathway	**1**					**1**	**3**	**3**
** Folding, sorting and degradation**								
03060 Protein export		**1**			**3**	**1**	**4**	**4**
04141 Protein processing in endoplasmic reticulum	**3**	**4**		**1**	**19**	**3**	**18**	**5**
04120 Ubiquitin mediated proteolysis					**7**	**1**	**4**	**1**
03050 Proteasome					**1**		**5**	
03018 RNA degradation					**4**	**2**	**4**	**3**
**ENVIRONMENTAL INFORMATION PROCESSING**								
** Signal transduction**								
04016 MAPK signaling pathway - plant		**3**			**11**	**1**	**11**	**1**
04070 Phosphatidylinositol signaling system		**1**		**1**	**2**	**1**	**3**	**2**
04075 Plant hormone signal transduction					**11**	**7**	**10**	**3**
**CELLULAR PROCESSES**								
**Transport and catabolism**								
04144 Endocytosis	**2**	**2**			**5**	**7**	**6**	**2**
04145 Phagosome	**2**	**1**			**4**	**2**	**7**	**1**
04146 Peroxisome	**1**	**1**			**5**	**1**	**7**	**5**
**ORGANISMAL SYSTEMS**								
**Environmental adaptation**								
04712 Circadian rhythm - plant		**3**			**2**	**1**	**1**	
04626 Plant-pathogen interaction		**4**			**9**	**2**	**11**	**6**

legend: Numbers refer to key enzymes represented by *Picea abies* KAAS assigned orthologous. For 39 dpi, only symptomatic needles were considered. To simplify the table, only those pathways with at least 3 key enzymes represented at one of the time points where considered (for full table see Additional file 5: Table S3)

**Fig. 6:**
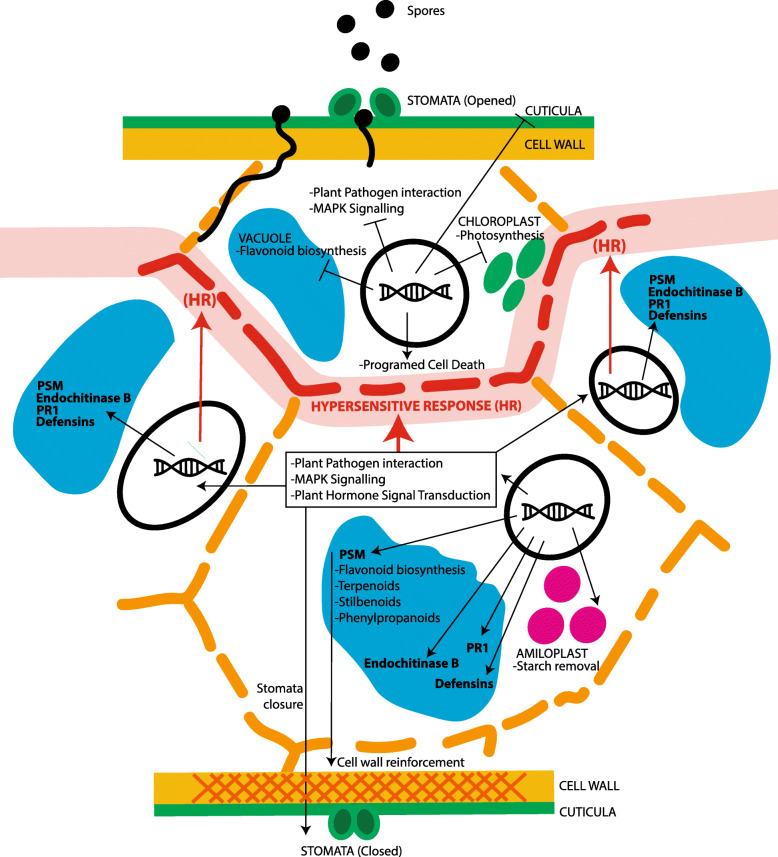
Overview of the complex defense response. Shown are the main activated and inhibited pathways and processes in the different compartments of *Picea abies* cells during infection by *Chrysomyxa rhododendri*

Several pathways were identified to be probably involved in the defense (Table 2) of *P. abies* against *C. rhododendri* comprising a total set of 152 DEGs (Additional file 7: Table S5). Several play a role in signal transduction and environmental adaptation (plant-pathogen interaction, MAPK signaling and plant hormone signal transduction), biosynthesis of plant secondary metabolites (phenylpropanoid, flavonoid, flavone and flavonol, stilbenoid, diarylheptanoid and gingerol, terpenoid backbone), lipid biosynthesis (cutin, suberine and wax), and amino acid metabolism (phenylalanine, tyrosine and tryptophan biosynthesis). At the early stage of infection (4 dpi), several key enzymes of these pathways were under-expressed (Table 2, Fig. 6), and after 21 dpi, the majority of key enzymes were over-expressed (Additional file 8: Figure S3). Interestingly, many key genes related to translation machinery were under-expressed in later infection stages, and few genes from cutin, suberine and wax biosynthesis were under-expressed during the entire period investigated.

### Symptomatic versus non-symptomatic needles

Only few transcripts were differentially expressed in 39 dpi non-symptomatic needles compared to control plants (Additional file 3: Table S1E) and we thus focused our analysis on transcripts differentially expressed in 39 dpi symptomatic needles compared to control plants. However, it is remarkable that of the differentially expressed transcripts found by comparing 39 dpi symptomatic needles to 39 dpi non-symptomatic needles (Additional file 3: Table S1F), 61.45% were shared with 39 dpi symptomatic needles vs. control DEGs (Additional file 9: Table S6A). In addition, Gene Ontology (GO) enrichment analysis and metabolic pathway analysis revealed similar patterns for both comparisons (Additional file 9: Table S6B-F).

### RT-qPCR validation of selected DEGs

**Fig. 7:**
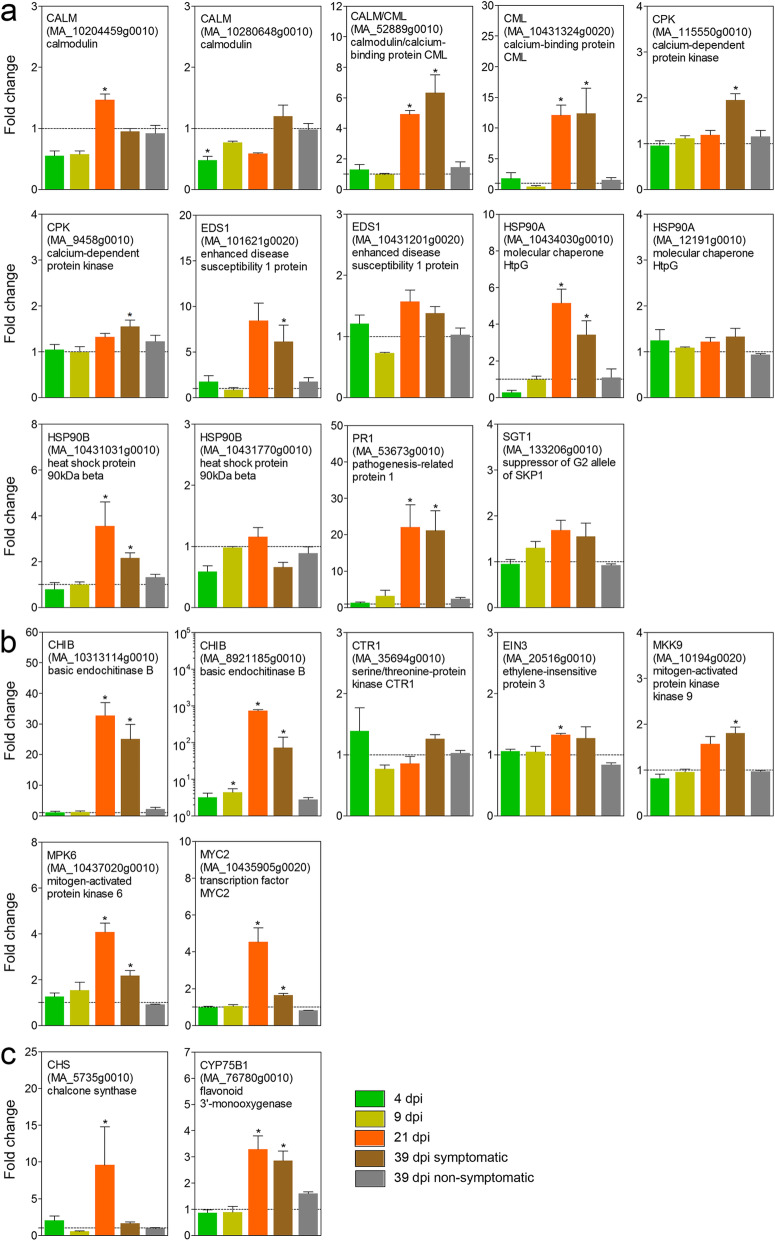
RT-qPCR results of differentially expressed transcripts assigned to the pathways (**a**) plant-pathogen interaction, (**b**) MAPK signaling and (**c**) flavonoid biosynthesis. Relative expression levels (mean fold change ± SE, *n* = 3) were compared to control plants (set to 1) at 4 dpi, 9 dpi, 21 dpi and 39 dpi (symptomatic and non-symptomatic needles). *P < 0.05

23 DEGs assigned to the significantly enriched pathways plant-pathogen interaction, MAPK signaling and flavonoid biosynthesis were validated by RT-qPCR (Fig. 7). Overall RNA-Seq and RT-qPCR results were in a good agreement. Expression changes detected by RNA-Seq could be verified for the majority of transcripts. Only the over-expression of serine/threonine-protein kinase CTR1 (MA_35694g0010) at 21 dpi and 39 dpi symptomatic vs. non-symptomatic was not detected by RT-qPCR (Additional file 10: Figure S4). The most prominent expression changes were detected by RT-qPCR at 21 dpi and 39 dpi symptomatic needles (Fig. 7). The highest levels of over-expression at 21 dpi were found for the basic endochitinase B gene (CHIB, MA_8921185g0010: 738-fold increase; and MA_10313114g0010: 33-fold) and the pathogenesis-related protein 1 (PR1, MA_53673g0010: 22-fold). Under-expression among the selected transcripts was only detected at the early time points of infection. No major changes were found in non-symptomatic needles at 39 dpi.

### Changes in phenolic profiles

**Fig. 8:**
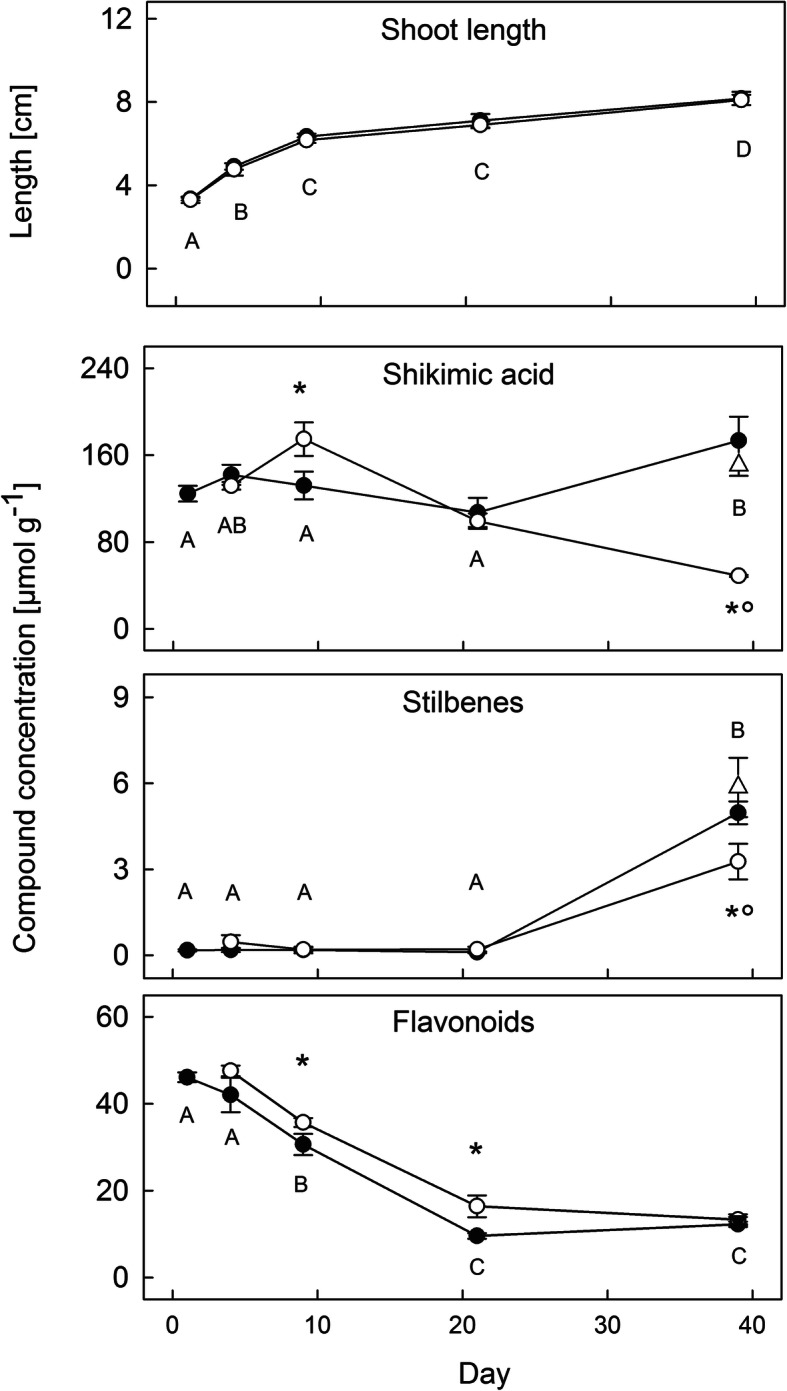
Concentration changes of phenolic needle metabolites. Shoot elongation and compound concentrations from 1 to 39 dpi in needles of control (filled symbols, mean ± SE, n = 3) and spore exposed spruce cuttings (open symbols, mean ± SE, n = 3). Triangles on day 39 indicate concentrations in healthy needles of treated cuttings (mean ± SE, n = 3). Concentration values are given as μmol g^−1^ dry weight. Significant differences between time points are marked with different letters, between the control and treated group with asterisks and between healthy and symptomized needles of infested cuttings on day 39 with a circle. Patterns of all individual compounds measured are shown in Additional file 11: Fig. S5

Needles of control plants showed a rather constant concentration of shikimic acid during the experiment, while flavonoid concentrations constantly decreased until 21 dpi and stilbenes strongly accumulated towards the end (Fig. 8). In cuttings attacked by the fungus, significantly higher levels of shikimic acid were detected at 9 dpi, which decreased constantly to far below the level of control plants until 39 dpi. Flavonoid concentrations significantly increased in infected plants at 9 and 21 dpi, mainly due to an increase of kaempferol and quercetin on 9 dpi and of quercetin 3-glucoside on 21 dpi (Fig. 8, Additional file 11: Figure S5). Additionally, taxifolin significantly increased in infected symptomatic needles at 39 dpi. In contrast, stilbene accumulation was reduced in attacked cuttings, mainly due to lower concentration of the compound astringin. Healthy and symptomized needles originating from the same shoot of treated plants at 39 dpi differed strongly in their phenolic composition (Fig. 8). The healthy needles corresponded precisely to needles of control plants with respect to all analysed metabolites (Fig. 8, Additional file 11: Figure S5).

## Discussion

Plant defence systems against pathogens are well studied in model organisms and crops, but largely unexplored in conifers. In the present study, we combined RNA-Seq and LC-MS to investigate the defense response of Norway spruce infected by *C. rhododendri* [25, 27, 28]. This combined approach has been proven to be very useful in the study of molecular defense mechanisms of host plants against pathogens [63, 69, 90].

RT-qPCR was used to validate the RNA-seq results. Expression changes detected by RNA-seq could be verified for 22 transcripts out of the 23 DEGs selected for validation (Additional file 10: Figure S4). Differences in the relative expression values might be explained by the different normalisation processes implemented by the two methods. The combination of both datasets, however, improves the identification of the most affected targets, especially for those *P. abies* transcripts that were assigned to identical gene functions.

### General host response

An overall analysis of GO Enrichment terms showed that from the beginning of the infection process (4 dpi) onwards, numerous genes related to stress responses were differentially regulated (Fig. 5). In particular, enriched terms at 21 dpi indicated an activation of genes related to plant defense, plant secondary metabolism, signal transduction and kinases, which may play important roles in signaling, communication and coordination of defense reactions. Also, enriched terms indicated effects at the thylakoids, which is in agreement with reductions in chlorophyll content and photosynthesis observed in field studies [64]. Over-expressed transcripts at the end of the infection experiment (39 dpi) related to cell death suggest the occurrence of a hypersensitive response (HR), which prevents the spread of infections by the rapid death of cells surrounding the infected area [54]. Also many key enzymes from the ubiquitin mediated proteolysis and proteasome were over-expressed from 21 dpi on, which play a key role in plant immunity [18, 81, 85] and degradation of unneeded or damaged proteins in cells undergoing programmed cell death (Table 2). Accordingly, distinct necrotic areas in partly infected Norway spruce needles were described in Mayr et al. [66]. At 39 dpi several key enzymes related to endocytosis, phagosome [70] and peroxisome [38, 45] were over-expressed. The onset of this strong defense response occurred relatively late compared to other pathosystems (reactions within hours or days; e.g. in [90, 94]) which may be due to overall low metabolic activity in trees adapted to high elevation and extreme climatic conditions [37].

### Plant-pathogen interaction and signal transduction

During infection, plant-pathogen interaction pathways were considerably differentially regulated (Table 2, Fig. 6, Additional file 7: Table S5, Additional file 8: Figure S3A). Among identified DEGs, some are involved in recognition of pathogen-associated molecular patterns (PAMPs) by plasma membrane receptors (PAMP-triggered immunity PTI), while others are involved in the detection of pathogen effectors by intracellular or plasma membrane–localized immune receptors (effector-triggered immunity - ETI) (review by [75]). Interestingly, at 4 dpi two calmodulins (MA_10204459g0010; MA_10280648g0010) and two HSP90 (MA_10431770g0010 and MA_10434030g0010) were under-expressed, indicating blockage of the Ca^2+^ signaling pathway and thus the defense response via HR, cell wall reinforcement and stomata closure, and probably enabled *C. rhododendri* to colonize other tissues [55, 67]. Rust fungus can interfere with a variety of host defense pathways and suppress multiple plant defense responses [59, 78]. In contrast, at 21 dpi, 9 key enzymes (22 genes) involved in both PTI and ETI and including several calmodulines as well as HSP90 were over-expressed. It is likely that Norway spruce, at this infection phase, activated defense reactions against the fungus via stomatal closure, hypersensitive response (HR) and cell wall reinforcement [30]. This response is also promoted by the over-expression of MA_9458g0010, a calcium-dependent protein kinase (CDPK) putatively involved in abscisic acid-activated signaling pathway and intracellular signal transduction upon phosphorylation by fungal elicitors. Finally, DNA expression promoted by this signaling pathway enhanced the expression of two (MA_501572g0010, MA_53673g0010) pathogenesis-related protein 1 (PR1), which is induced in response to a variety of pathogens and was shown to be a useful molecular marker for the salicylic acid-mediated disease resistance [10].

Furthermore, the MAPK signaling pathway and plant hormone signal transduction were differentially regulated: In both cases, at least 11 key enzymes were over-expressed from 21 dpi on, which again indicates that the fundamental plant defense reactions started between 9 and 21 dpi (Additional file 8: Figure S3B-C). In the MAPK signaling pathway, mitogen-activated protein kinase 6 (MPK6: MA_10437020g0010) is a key protein in several signaling routes and plays a critical role in plant disease resistance [68]. Jagodzik et al. [41] reported a complex crosstalk between the MAPK cascade and hormone signaling pathways to orchestrate plant defense. Accordingly, in infected Norway spruce, over-expressed genes included serine/threonine-protein kinase (CTR1: MA_35694g0010), mitogen-activated protein kinase kinase 9 (MKK9: MA_10194g0020), ethylene-insensitive protein 3 (EIN3: MA_20516g0010) and transcription factor MYC2 (MYC2: MA_10435905g0020), promoting defense responses via DNA expression of several genes as basic endochitinases B (MA_10313114g0010; MA_8921185g0010), an effective defense against chitin-containing fungal pathogens [42, 44, 51, 87]. In addition, plant defensins well known efficient antimicrobial peptides against fungi [53] were also over-expressed (MA_5320g0010, MA_1489g0010, MA_4353361g0010) in response to infection [72]. In parallel, plant growth is affected by under-expression of several genes involved in the auxin signaling pathway, contributing to an optimized use of resources during this biotic stress.

### Primary and secondary metabolism

Both the primary and secondary metabolism [4] of infected plants were highly affected, especially from 21 dpi on (Table 2, Fig. 6). The over-expression of genes involved in the biosynthesis of secondary metabolites (Additional file 8: Figure S3 D-I) is one of the frequently observed response patterns [3, 12, 13], and frequently comes at the cost of an under-expression of key enzymes involved in photosynthesis and carbon fixation. An extensive list of genes involved in photosynthesis were under-expressed, in accordance with the yellow discoloration of rust infected needles and the documented reduction in chlorophyll content and photosynthetic activity [7, 64]. In contrast, many key enzymes of starch and sucrose metabolism were over-expressed by the end of the infection process and DEGs indicated increased glycolysis. Lipid metabolism was also affected and several genes involved in cutin, suberin and wax biosynthesis were under-expressed during infection (Additional file 8: Figure S3 J). Infected cuttings obviously changed their metabolism from autotrophy (photosynthesis) to a consumption of resources [80, 82] and optimized limited resources by use of lipids on alternative pathways [56].

Many transcripts differentially expressed in infected needles are involved in the biosynthesis of secondary metabolites, among them phenolic compounds and terpenoids. The antifungal activity of these compounds has been extensively described [14, 32, 34, 35, 52, 77] and they are considered to play a central role in conifer response to fungal pathogens [5, 11, 14, 62, 92], including spruce response to needle rust [28]. Accordingly, numerous pronounced effects were observed in the infection experiment: In the terpenoid backbone biosynthesis pathway genes belonging to the mevalonate pathway (cytoplasm) were over-expressed in comparison to the non-mevalonate pathway (plastid) (Additional file 8: Figure S3 I). Variations in phenolic profiles were detected from 4 dpi on, whereby concentrations of individual compounds showed highest changes at 9 dpi (increased concentration levels) and 39 dpi (mainly decreased concentration levels). Please note that constitutive concentration levels (in healthy spruce needles) also changed considerably during the experiment (Fig. 8, Additional file 11: Figure S5), as the phenolic profile changes during needle development [28]. A key molecule in the plant secondary metabolism is shikimic acid, as it is the precursor of phenylalanine, tryptophan, tyrosine (Additional file 8: Figure S3D), and subsequently for the main phenolic pathway [88]. Therefore, the increase and decline of shikimic acid at 4 and 9 dpi can be directly related to a suppressed or enhanced phenolic production downstream. In addition, at 9 dpi an over-expressed transaldolase on the pentose phosphate pathway (MA_30531g0010), which produces the precursor D-erythrose 4-phosphatemay explain the increased concentrations in infected plants. It remains unclear, why later (39 dpi), shikimic acid was reduced in infected compared to control trees: production may have been limited due to reduced photosynthetic capacity and respective resources, or due to under-expression of the first key enzyme on the shikimate pathway (3-deoxy-7-phosphoheptulonate synthase; MA_54864g0010). Alternatively, shikimic acid may have been consumed downstream by the production of plant secondary metabolites, as indicated by over-expression of many phenylpropanoid and flavonoid biosynthesis genes from 21 dpi on [16].

Due to the complexity and interconnection of phenolic pathways and the different nature of involved enzymes it is hardly possible to directly link over- and under-expressed genes in the individual pathways directly to measured metabolite concentrations. However, chalcone synthase (*CHS*: MA_20764g0010 and MA_5735g0010), a key enzyme related to the flavonoid biosynthesis, was under-expressed at 4 dpi and over-expressed at 21, and corresponded well to flavonoids concentration at these time points (Fig. 8, Additional file 8: Figure S3G). Up-regulation of chalcone synthase was shown to be central in the defence mechanisms of Norway spruce [73]. In addition, over-expression of a flavonoid 3`-monooxygenase (*CYP75B1*: MA_76780g0010) at 21 dpi and 39 dpi corresponded to concentration changes in quercetin 3-glucoside. Nevertheless, Flavanone-3-hydroxylase and Flavonoid 3′,5′-hydrolase genes that plays an important role in the biosynthesis of spruce phenolic defenses against bark beetle-associated fungus *Endoconidiophora polonica* [34, 35] were not differentially expressed here. Different pathogens and infected tissues may result in different responses even some similarities can be found. Here, high concentration of taxifolin in symptomatic needles is remarkable as it is a relevant antifungal flavonoid induced by various infections [11, 21, 28, 35, 49].

On the other hand the decline of picein and stilbene concentrations at 39 dpi, when needle tissues were densely pervaded by fungal hyphae, may be explained either (i) through the reduction on investments in the plant defence when tissue damages are already severe [7], (ii) metabolization of those compounds by the fungus (see e.g. [33]) or (iii) conversion, modification, and incorporation into the cell wall [23].

Like in the flavonoid biosynthesis, also in the phenylpropanoid biosynthesis pathway a large number of genes were over-expressed at 21 and 39 dpi, but partially also at 4 dpi (Additional file 7: Table S5, Additional file 8: Figure S3E). In particular, several phenylalanine ammonia lyases (*PAL*), and some peroxidases (*PX3*), but likewise also a class IV chitinase (CHI4; MA_10427514g0010 were differentially over-expressed as reported also for spruce infected by *Ceratocystis polonica* [72] and *Heterobasidion annosum* [36]. In any case, combined RNA-Seq and biochemical data suggest that the rust infection provoked complex and dynamic regulative changes in the phenolic pathways, leading to altered metabolite concentration in damaged spruce needles.

### Local versus systemic defence response

The comparison of 39 dpi symptomatic and non-symptomatic needles confirms the local activation of molecular defence mechanisms based on both inducible chemical defence (plant secondary metabolites) and low-molecular-weight proteins (Fig. 6). Interestingly, we observed no systemic induction of defence response in healthy needles of infected cuttings, neither on the RNA expression nor on the metabolic level (Figs. 2 and 8). Similar defence response was found under *Ceratocystis polonica* infection [50]. Systemic acquired resistance represents, besides constitutive and inducible defence, a third defence strategy, and was hypothesized to be a common phenomenon also in conifers [9, 49, 92]. However, as the complex life cycle of *C. rhododendri* limits infection events to a short period of the year, an acquired resistance may be of little advantage and thus not outweigh the respective costs for the plant.

## Conclusions

Norway spruce shows a locally activated response to infection by *C. rhododendri,* which starts at 4 dpi but is pronounced between 9 and 21 dpi and mainly aims at isolating the fungus by a hypersensitive response (HR) in conjunction with an activation of phenolic pathways. This has been extensively described also for other pathosystems involving plants and fungi [15, 90] and probably differs between resistant and susceptible spruce phenotypes [16]. The presented study represents a solid basis for further specific analyses of identified key regulatory genes in clones with varying resistance.

## Methods

### Plant material and experimental design

The experiment was conducted with five-year-old cuttings gained from the Norway spruce clone ASS-7, originating from a highly needle rust affected subalpine site in Tyrol, Austria [27]. The adult tree ASS-7 was shown to exhibit a slightly lower percentage of infected needles compared to surrounding trees in the field, but nevertheless showed clear symptoms of needle rust infection. We therefore expected this clone to show pronounced responses upon infection on the molecular level. By using genetically identical cuttings for both the infection treatment and controls we avoided genotype influences on gene expression patterns and enabled three clonal replicates within the groups. Cuttings were grown in pots under field conditions in the forest garden Bad Häring, Tyrol, Austria and, one month before start of the treatment, six equally grown individuals were moved to the Botanical Garden of the Department of Botany of the University of Innsbruck.

The experiment was performed in a greenhouse to control climatic and infection conditions, but simulated the field situation, where Norway spruce is constantly exposed to this pathogen spores during the infection period. Inoculation treatment was started when buds opened and needles started to unfold, which corresponds to the period when needles are susceptible to the fungus [26]. Cuttings were split into a treatment (*n* = 3) and control (n = 3) group and placed in two different custom made plastic tents sized 1x1x1m. Tents were equipped with fans for better fungal spore distribution and with microscopic slides for monitoring of spore densities (Fig. 1). In the infection tent, cuttings were exposed to *C. rhododendri* spores by placing rhododendron branches with mature spore stocks above the cuttings. Spore exposure was continued from day 1 to day 21, to mimic a situation that plants face in the natural environment. All plants were regularly watered and repeatedly randomly rearranged within the tent to ensure uniform spore exposure. Spore deposition within the exposure time equalled 2.04 ± 0.17 spores per mm^2^. On infected cuttings, 51 to 76% of current year needles developed clear infection symptoms, while control cuttings remained completely healthy. First infection symptoms (small blisters at the needle surface) appeared 21 days after start of the treatment, while the clear yellow discoloration was visible one week later (Additional file 2: Figure S2).

Needle samples were taken at 1, 4, 9, 21, and 39 days post infection (dpi) by cutting one current-year shoot per plant and distributing needles on two RNase-free tubes for (a) RNA-Seq analysis and (b) phenolic analysis. At 1 dpi, only control plants were sampled. At 39 dpi, samples from infected trees were further subdivided into symptomized and healthy needles (Fig. 1), as infection symptoms were clearly visible. Samples were immediately submersed in liquid nitrogen and stored at − 80 °C until further analysis.

### RNA isolation, sequencing and mapping to reference genome

RNA was extracted from a total of 30 Norway spruce samples by homogenizing needle samples with mortar and pestle and subsequently mixing with lysis solution in order to preserve RNA integrity. The Protocol B Sigma-Aldrich Spectrum Plant Total RNA Kit (PN: STRN50-1KT) was used for extraction according to the manufacturer’s guidelines (Sigma Aldrich, St. Louis, USA). RNA concentrations were measured with a NanoDrop 2000c spectrophotometer (Thermo Fisher Scientific, Waltham MA, USA) and approximately 1 mg of DNAse I treated RNA was diluted in a total reaction volume of 10 μl. Quality of DNAse I treated total RNA was determined by using an Agilent 2100 Bioanalyzer with RNA 6000 Nano kit as plant run mode class (Agilent technologies, Santa Clara CA, USA). 5 μl of DNAse I digested total RNA was processed with QuantSeq 3′ mRNA-Seq FWD Library Preparation Kit and each reaction was spiked in with SIRV-Set 3 variant controls by following the manufacturer’s guidelines (Lexogen, Vienna, Austria). Quality of the libraries was determined with an Agilent 2100 Bioanalyzer DNA High Sensitivity Kit (Agilent technologies, Santa Clara, USA). Samples were pooled in equimolar ratio and the library pool was quantified using aQubit dsDNA HS assay kit (Thermo Fisher Scientific, Waltham MA, USA). Sequencing was performed on an Illumina NextSeq 500 system with SR75 High Output Kit at Lexogens (Lexogen, Vienna, Austria). The obtained reads were mapped to the Norway spruce reference genome [74] by using the open-source software STAR [19]. We used the alternate protocol 1 as described in Dobin & Gingeras [19] and only those contigs that had a gene annotation in the Norway spruce genome assembly were retained for mapping. Since the Norway spruce reference genome is approximately 20 GB in size [74], genome indices were produced by using a 189 GB RAM server according to the required Genome size/bytes ratio described in Dobin & Gingeras [19]. The detailed STAR settings used for mapping and genome index generation can be found in the Additional file 12: Command S1.

### Differential gene expression analysis

For differential gene expression analysis the DESeq2 package [60], which is implemented in the *Bioconductor* platform in R [29], was used. Briefly, DESeq2 transforms read counts per gene obtained from RNA-Seq data into systematic contrasts of gene expression across experimental conditions. For this, DESeq2 uses so called shrinkage estimators which were shown to better estimate and compare fold changes in gene expression across treatments compared to other available methods [60]. The null hypothesis tested implies that the logarithmic fold change in gene expression between infected and control plants is exactly zero. We first used the *DESeqDataSetFromHTSeqCount* function for importing read count data and pre-filtered for rows with zero counts. Subsequently, differential gene expression analysis was performed by using the *DESeq* function which generates the log2fold changes with adjusted *p*-values for each treatment per time point combination (infected vs control at 4, 9, 21, 39 dpi symptomatic and non-symptomatic needles). Differential gene expression analysis was performed also for 39 dpi symptomatic vs 39 dpi non-symptomatic needles.

Venn diagrams of over- and under-expressed transcripts were created by using jvenn [6]. Nowadays, ConGenIE database (http://congenie.org/enrichment) [84] provides useful information of the ongoing *P. abies* sequencing project, even if genes are not yet annotated hindering the work of mining. Nevertheless, gene role or function can be investigated further by using as starting point its PFAM domains or Gene ontology (GO) terms. GO enrichment analysis with Fisher exact tests on differentially expressed transcripts was performed with functional enrichment analysis tools available at ConGenIE, using p-values corrected with a false discovery rate of (FDR) < 0.05. GO-Slim was run to reduce complexity of GO terms for gene class analysis. Based on GO-Slim annotations, over- and under-expressed transcripts were classified into three ontological categories: cellular component, biological process, and molecular function.

Homology is a fruitful tool for mapping conserved genes (orthologous) and here we try to elucidate changes in well described and characterized pathways and genes. Metabolic pathway and orthology-oriented functional annotations of the protein sequences (.fasta files downloaded at ConGenIE) were performed against the Kyoto Encyclopedia of Genes and Genomes (KEGG) database [39] using the KEGG Automatic Annotation Server (KAAS; https://www.genome.jp/tools/kaas/) [71]. KEGG Orthology (KO) assignment was applied using the Bi-directional Best Hit (BBH) method and all datasets of dicot plants (excluding *Camelina sativa*, *Lupinus angustifolius* and *Cucurbita moschata*), monocot plants and a basal magnoliophyta (*Amborella* family) were selected as reference organisms (Additional file 13: Table S7). This analysis was applied at each time point for differentially over- and under-expressed genes (DEGs). KEGG Orthology (KO) output htext was converted to tabulated excel file (Additional file 6: Table S4). Norway spruce pathway hierarchy model was created by merging several plant reference pathway hierarchies available at KEGG (Additional file 14: Table S8) and this was used to standardize the KO assignment results (Additional file 6: Table S4).

### RT-qPCR

Twenty three DEGs were selected for RT-qPCR validation. Primers were designed using the PrimerQuest tool (Integrated DNA Technologies, Coralville, IA, USA). RT-qPCR assay details are listed in Additional file 15: Figure S6. The amplification efficiency of each assay was calculated based on the standard curve from a four-fold dilution series of cDNA (0.025–100 ng). Seven candidate reference genes (RGs) were tested on a selection of 21 *P. abies* test RNAs that reflect the experimental setup. The expression stability of the RGs was assessed using the RefFinder tool [93]. Two of the most stably expressed genes, actin (ACT) and ubiquitin (UBI), were selected for normalisation (Additional file 15: Figure S6). Reverse transcription (RT) of 1 μg total RNA was done with the SuperScript III First-Strand Synthesis SuperMix (Invitrogen, Carlsbad, CA, USA) with oligo (dT) primers according to the manufacturer’s protocol. No RT controls (without enzyme mix) were included to monitor the amplification of contaminating DNA. RT-qPCR reactions were conducted in 20 μl reaction volumes including 1x HOT FIREPol EvaGreen qPCR Mix Plus ROX (Solis BioDyne, Tartu, Estonia), 200 nM of each primer and 25 ng cDNA. Each sample was analysed in duplicates on the AriaMx Real-time PCR System (Agilent, Santa Clara, CA, USA) with the following temperature protocol: initial activation at 95 °C for 12 min, 40 cycles of 95 °C for 15 s and 60 °C for 1 min, followed by a melting curve analysis step (60 °C - 95 °C). The efficiency-corrected target gene Cq values were normalised to the geometric mean of ACT and UBI, and relative expression changes compared to non-infected controls and for 39 dpi also for symptomatic vs. non-symptomatic needles, were calculated with the comparative 2^-ΔΔCT^ method [58]. Mean Cq values obtained by RT-qPCR are enclosed in Additional file 16: Table S9. Statistical analyses (two-tailed unpaired t-test with Welch’s correction) of the RT-qPCR data were done GraphPad Prism 5 software (GraphPad Software, San Diego, CA, USA). A *p* value of < 0.05 was considered as significant.

### Identification and quantification of phenolic compounds

Phenolic analysis was conducted as described in Ganthaler et al. [28] and included the identification and quantification of eleven flavonoids (kaempferol, kaempferol 3-glucoside, kaempferol 7-glucoside, kaempferol 3-rutinoside, quercetin, quercetin 3-glucoside, quercitrin, naringenin, taxifolin, catechin, gallocatechin), five stilbenes (astringin, isorhapontin, piceid, piceatannol, resveratrol), three simple phenylpropanoids (picein, gallic acid, chlorogenic acid) and one precursor (shikimic acid). Briefly, needles were freeze-dried, homogenized, and extracted two times with 1 ml 95% (v/v) ethanol, containing 2 μmol L^− 1^ orientin, pinosylvin and naringin as internal quantification standards. Liquid chromatography-mass spectrometry (UHPLC-MS) was conducted using an ekspert ultraLC 100 UHPLC system with a reversed-phase UHPLC column (NUCLEODUR C18 Pyramid, EC 50/2, 50 × 2 mm, 1.8 μm, Macherey-Nagel, Düren, Germany) coupled to a QTRAP 4500 mass spectrometer (AB SCIEX, Framingham, MA, USA) operated in negative ion mode using multiple reaction monitoring (MRM). Based on retention time and MRM transition (for detailed parameters see [28]), and calibration curves of authentic samples of all substances, peaks were automatically detected and normalized relative to the internal standards, and concentrations were calculated using the software Analyst (version 1.6.3) and MultiQuant (version 2.1.1, both AB SCIEX, Framingham, MA, USA).

For phenolic concentrations, differences were tested (1) between time points, (2) between the control and treatment group, and (3) between healthy and symptomized needles of the treatment group at 39 dpi. Differences were analysed with the Bonferroni test (for data with homogeneity of variance) or Tamhane test (no homogeneity of variance) after testing for homogeneity (Levene test) and for Gaussian distribution (Kolmogorov–Smirnov test). All tests (two-tailed) were performed pairwise at a probability level of 5% using SPSS (version 24; SPSS, IL, USA), and all values are given as mean ± standard error (SE).

## Supplementary information

**Additional file 1: Figure S1.** Inter-replicate correlation plots. Inter-replicate correlation of (a) sample R1 (Baseline control T_1_), (b) sample R4 (Control T_4_), (c) sample R9 (Control T_9_), (d) sample R21 (Control T_21_), (e) sample R39 (Control T_39_), (f) sample R4 (Infected T_4_), (g) sample R9 (Infected T_9_), (h) sample R21 (Infected T_21_), (i) sample R39 (Infected T_39_ no symptoms), (j) sample R39 (Infected T_39_ clear symptoms). (k) Pearson correlation coefficients.

**Additional file 2: Figure S2.** Symptoms of *C. rhododendri* infection at 21 and 39 dpi. At 21 dpi, first symptoms of infection were detectable under the microscope, i.e. small blisters on the needle surface become visible (see arrows). At 39 dpi, several current-year-needles of infected trees showed the characteristic yellow discoloration and first aecio spore stocks were formed.

**Additional file 3: Table S1.** Differentially expressed genes. Transcripts differentially expressed in infected compared to control plants in needles at 4 dpi (A), 9 dpi (B), 21 dpi (C). At 39 dpi, infected symptomatic needles were compared to control plants (D), infected non-symptomatic needles were compared to control plants (E), and infected symptomatic needles were compared to infected non-symptomatic needles (F). Over- and under-expressed genes are highlighted in red and green respectively. (XLS 1218 kb)

**Additional file 4: Table S2.** KEGG Orthology (KO) assignment and proportion. KO assignment to the *Picea abies* differentially expressed genes during infection by *Chrysomyxa rhododendri* and proportion of assignments at each time point. At 39 dpi symptomatic needles were compared to controls. (XLS 284 kb)

**Additional file 5: Table S3.** KEGG pathways of differentially expressed genes by *Picea abies* infected by *Chrysomyxa rhododendri*. Metabolic pathway and orthology-oriented functional annotations of the protein sequences were performed against the KEGG database using the KEGG Automatic Annotation Server. KEGG Orthology (KO) assignment was applied using the Bi-directional Best Hit (BBH) method. Numbers refer to key enzymes represented by *Picea abies* KAAS assigned orthologous. At 39 dpi symptomatic needles were compared to controls. (XLS 47 kb)

**Additional file 6: Table S4.** KEGG Orthology (KO) detailed output. This analysis was applied at each time point for differentially over- and under-expressed genes. Resulting hierarchical text (Htext) was converted to tabulated excel file and KO assignments resumed on a Norway spruce pathway hierarchy model for each time point. At 39 dpi symptomatic needles were compared to controls. (XLS 962 kb)

**Additional file 7: Table S5.** List of putative defense related genes differentially expressed by *Picea abies* infected by *Chrysomyxa rhododendri*. Genes arranged according to the related KEGG pathways (several genes are shared by more than one pathway). Selection was done based on literature, therefore also pathways and genes not included in this list could be relevant for defense response (see Additional file 6: Table S4). For 39 dpi, only symptomatic needles were compared with controls. (XLS 696 kb)

**Additional file 8: Figure S3.** KEGG pathways related to plant defence. A) Plant-pathogen interaction, B) MAPK signaling pathway-Plant, C) Plant hormone signal transduction, D) Phenylalanine, tyrosine and tryptophan biosynthesis, E) Phenylpropanoid biosynthesis, F) Stilbenoid, diarylheptanoid and gingerol biosynthesis, G) Flavonoid biosynthesis, H) Flavone and flavonol biosynthesis, I) Terpenoid backbone biosynthesis, J) Cutin, suberine and wax biosynthesis. At 39 dpi symptomatic needles were compared to controls.

**Additional file 9: Table S6.** Thirty nine dpi symptomatic vs. non-symptomatic (S-NS) differential expression analysis. A) DEGs overlapped between 39 dpi symptomatic vs. control (S-C) and 39 dpi symptomatic vs. non-symptomatic (S-NS), B) Gene ontology (GO) term enrichment analysis for symptomatic vs. non-symptomatic (S-NS) DEGs, C) KEGG Orthology assignment for symptomatic vs. non-symptomatic (S-NS) DEGs, D) KEGG pathways for symptomatic vs. non-symptomatic (S-NS) DEGs, E) KEGG Orthology detailed output for symptomatic vs. non-symptomatic (S-NS) over-expressed genes, GF) KEGG Orthology detailed output for symptomatic vs. non-symptomatic (S-NS) under-expressed genes. (XLS 61 kb)

**Additional file 10: Figure S4.** RT-qPCR validation of selected DEGs. Relative expression changes obtained by RT-qPCR (red bars; mean fold changes ± SE, *n* = 3) and RNA-seq (blue bars) compared to control plants at 4 dpi, 9 dpi, 21 dpi and 39 dpi (S-C). Symptomatic needles at 39 dpi were additionally compared to non-symptomatic needles (S-NS). Asterisk means significant (*p* < 0.05) difference compared to non-infected needles.

**Additional file 11: Figure S5.** Concentration changes of all phenolic needle metabolites. Concentration changes of individual phenolic compounds during the experiment in needles of control (filled symbols, mean ± SE, n = 3) and spore exposed spruce cuttings (open symbols, mean ± SE, n = 3). Triangles on day 39 indicate concentrations in healthy needles of treated cuttings (mean ± SE, n = 3). Concentration values are given as μmol g^− 1^ dry weight; compounds with needle concentrations below the quantification threshold (naringenin, quercitrin, piceid, piceatannol, resveratrol, chlorogenic acid, gallic acid) are not shown. Significant differences between the control and treated group are indicated with asterisks and between healthy and symptomized needles of treated cuttings on day 39 with a circle.

**Additional file 12:** Command S1. STAR command. The detailed STAR settings used for mapping and genome index generation.

**Additional file 13: Table S7.** Sequence datasets of plant species applied for KEGG Orthology (KO) assignment using the KEGG Automatic Annotation Server (KAAS). KEGG Orthology (KO) assignment was applied using the Bi-directional Best Hit (BBH) method and all datasets of dicot plants (excluding *Camelina sativa*, *Lupinus angustifolius* and *Cucurbita moschata*), monocot plants and a basal magnoliophyta (*Amborella* family) were selected as reference organisms. (XLS 1108 kb)

**Additional file 14: Table S8.** *Picea abies* pathway hierarchy model. This model was created by merging several plant reference pathway hierarchies available at KEGG and was used to standardize the KO assignment results (Additional file 6: Table S4). (XLS 25 kb)

**Additional file 15: Figure S6.** RT-qPCR assay details. Primer sequences of RT-qPCR assays.

**Additional file 16: Table S9.** RT-qPCR data. Mean efficiency-corrected Cq values obtained by RT-qPCR for (A) experimental samples and (B) determination of reference gene stability. (XLS 40 kb)

## Data Availability

Raw sequence data have been submitted to the NCBI Short Read Archive (SRA) under accession number PRJNA579736.

## Abbreviations

ACT: Actin
BBH: Bi-directional best hit
CDPK: Calcium-dependent protein kinase
DEGs: Differentially expressed genes
DPI: Days post infection
ETI: Effector-triggered immunity
FDR: False discovery rate
GO: Gene ontology
HR: Hypersensitive response
IR: Induced resistance
KAAS: KEGG automatic annotation server
KEGG: Kyoto encyclopedia of genes and genomes
KO: KEGG orthology
MPK/MAPK: Mitogen-activated protein kinase
MRM: Multiple reaction monitoring
NGS: Next generation sequencing
PAMPs: Pathogen-associated molecular patterns
PCA: Principal component analysis
PR: Pathogenesis-related proteins
PTI: PAMP-triggered immunity
QTLs: Quantitative trait locus
RGs: Reference genes
RNA-Seq: RNA-sequencing
RT: Reverse transcription
RT-qPCR: Quantitative reverse transcription PCR
SE: Standard error
UBI: Ubiquitin
UHPLC-MS: Ultra high performance liquid chromatography-mass spectrometry
