# Correction: RNA-Seq and secondary metabolite analyses reveal a putative defence-transcriptome in Norway spruce (*Picea abies*) against needle bladder rust (*Chrysomyxa Rhododendri*) infection

**DOI:** 10.1186/s12864-024-10831-z

**Published:** 2024-10-07

**Authors:** Carlos Trujillo-Moya, Andrea Ganthaler, Wolfgang Stöggl, Ilse Kranner, Silvio Schüler, Reinhard Ertl, Sarah Schlosser, Jan-Peter George, Stefan Mayr

**Affiliations:** 1 Federal Research and Training Centre for Forests, Landscape and Natural Hazards (BFW)-Department of Forest Genetics, Seckendorff-Gudent-Weg 8, 1131 Vienna, Austria; 2 Department of Botany, University of Innsbruck, Sternwartestraße 15, 6020 Innsbruck, Austria; 3 Federal Research and Training Centre for Forests, Landscape and Natural Hazards (BFW)- Department of Forest Growth & Silviculture, Seckendorff-Gudent-Weg 8, 1131 Vienna, Austria; 4 VetCore Facility for Research, University of Veterinary Medicine, Veterinärplatz 1, 1210 Vienna, Austria

**Correction:*BMC Genomics*** **21, 336 (2020)**

10.1186/s12864-020-6587-z

Following publication of the original article the following errors were reported for Additional files 13–15:

- The content of additional file 15 should have been the content of additional file 13.
- The content of additional file 13 were a duplicate of those of additional file 14.
- The correct content of additional file 15 was missing.

The correct additional files 13 and 15 are given in this Correction and the original article has been updated.

## Electronic supplementary material

Below is the link to the electronic supplementary material.

**Additional file 13:** Table S7. Sequence datasets of plant species applied for KEGG Orthology (KO) assignment using the KEGG Automatic Annotation Server (KAAS). KEGG Orthology (KO) assignment was applied using the Bi-directional Best Hit (BBH) method and all datasets of dicot plants (excluding Camelina sativa, Lupinus angustifolius and Cucurbita moschata), monocot plants and a basal magnoliophyta (Amborella family) were selected as reference organisms. (XLS 1108 kb)

**Additional file 15:** Figure S6. RT-qPCR assay details. Primer sequences of RT-qPCR assays.

