# Supplementary material for: RNA-Seq and secondary metabolite analyses reveal a putative defence-transcriptome in Norway spruce (Picea abies) against needle bladder rust (Chrysomyxa rhododendri) infection
Source: BMC Genomics. 2020 May 1;21:336. doi: 10.1186/s12864-020-6587-z (PMC7195740; doi:10.1186/s12864-020-6587-z)
Supplement: Supplementary file 1 — Additional file 1: Figure S1. Inter-replicate correlation plots. Inter-replicate correlation of (a) sample R1 (Baseline control T1), (b) sample R4 (Control T4), (c) sample R9 (Control T9), (d) sample R21 (Control T21), (e) sample R39 (Control T39), (f) sample R4 (Infected T4), (g) sample R9 (Infected T9), (h) sample R21 (Infected T21), (i) sample R39 (Infected T39 no symptoms), (j) sample R39 (Infected T39 clear symptoms). (k) Pearson correlation coefficients. [file 12864_2020_6587_MOESM1_ESM.pdf]

a

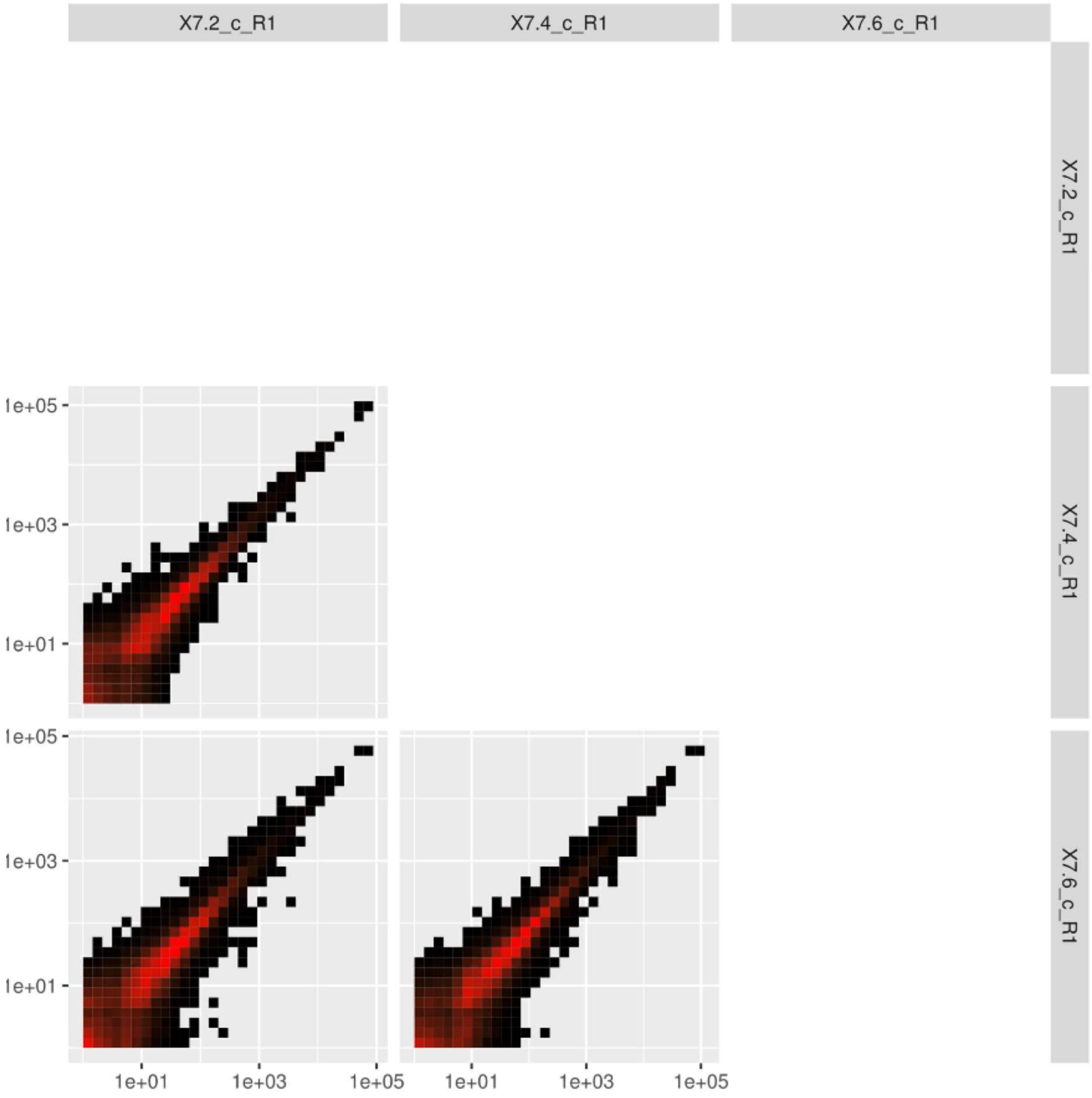

b

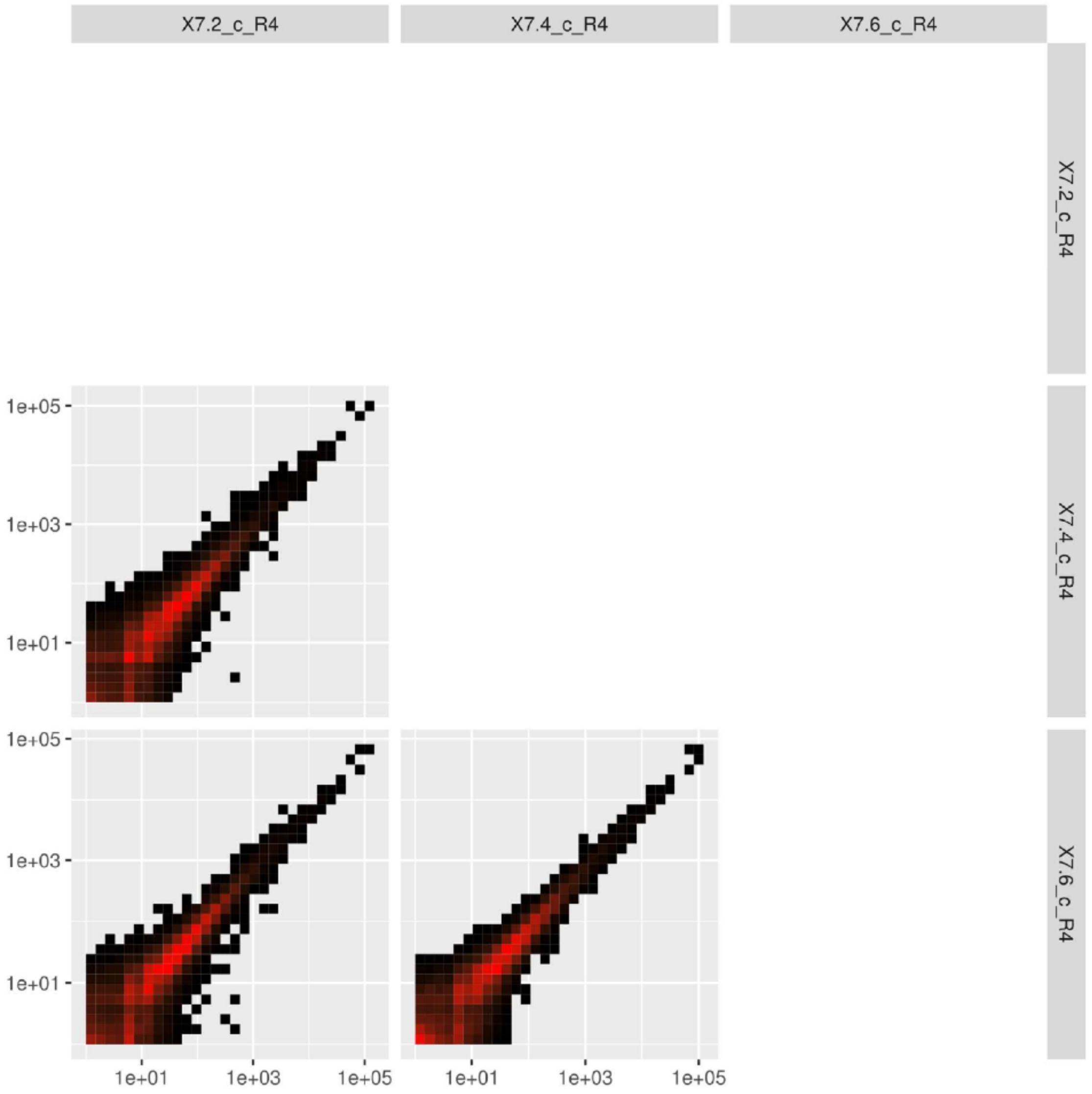

X7.2_c_R4
X7.4_c_R4
X7.6_c_R4
X7.2_c_R4
X7.4_c_R4
X7.6_c_R4
1e+05
1e+03
1e+01
1e+05
1e+03
1e+01
1e+01
1e+03
1e+05
1e+01
1e+03
1e+05

C

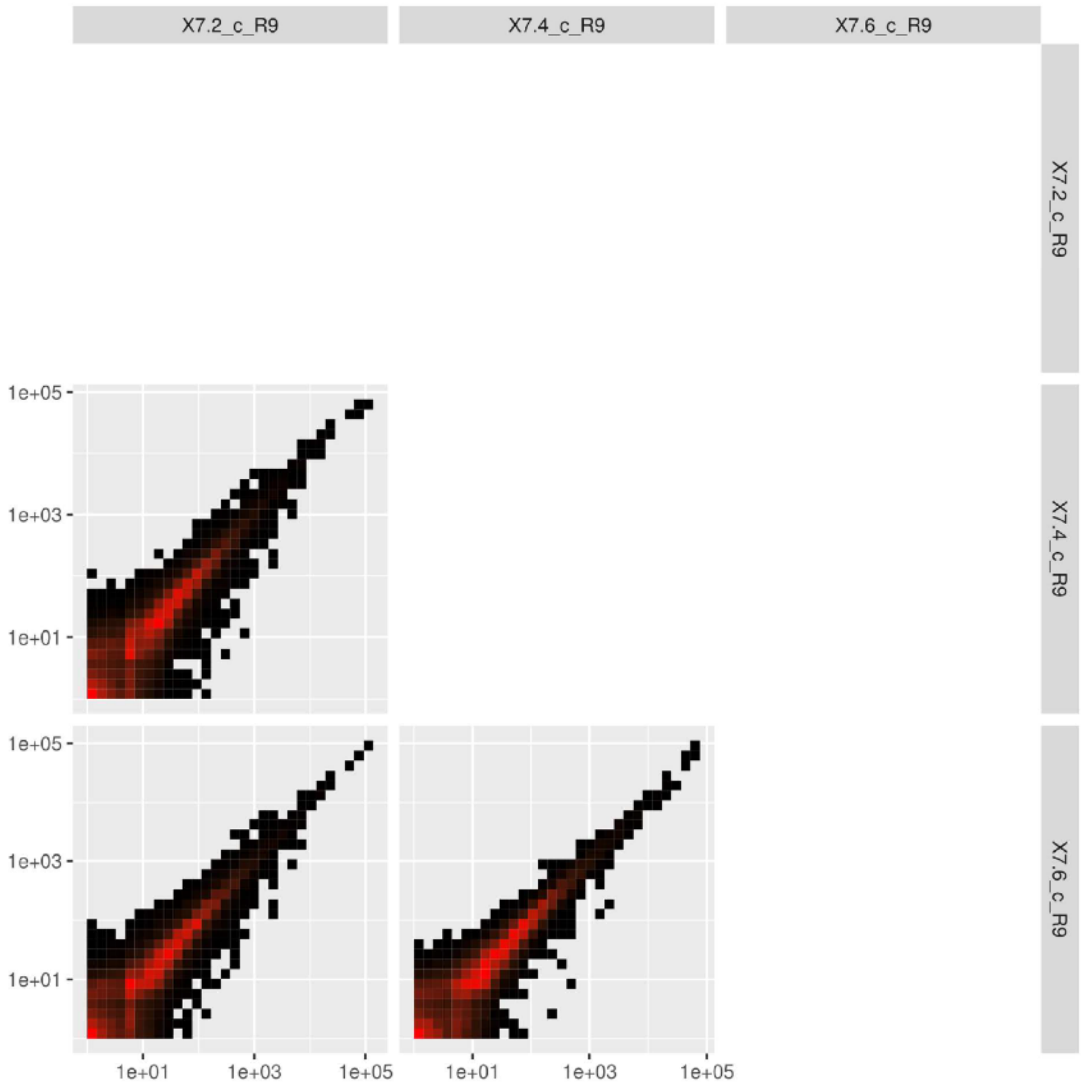

d

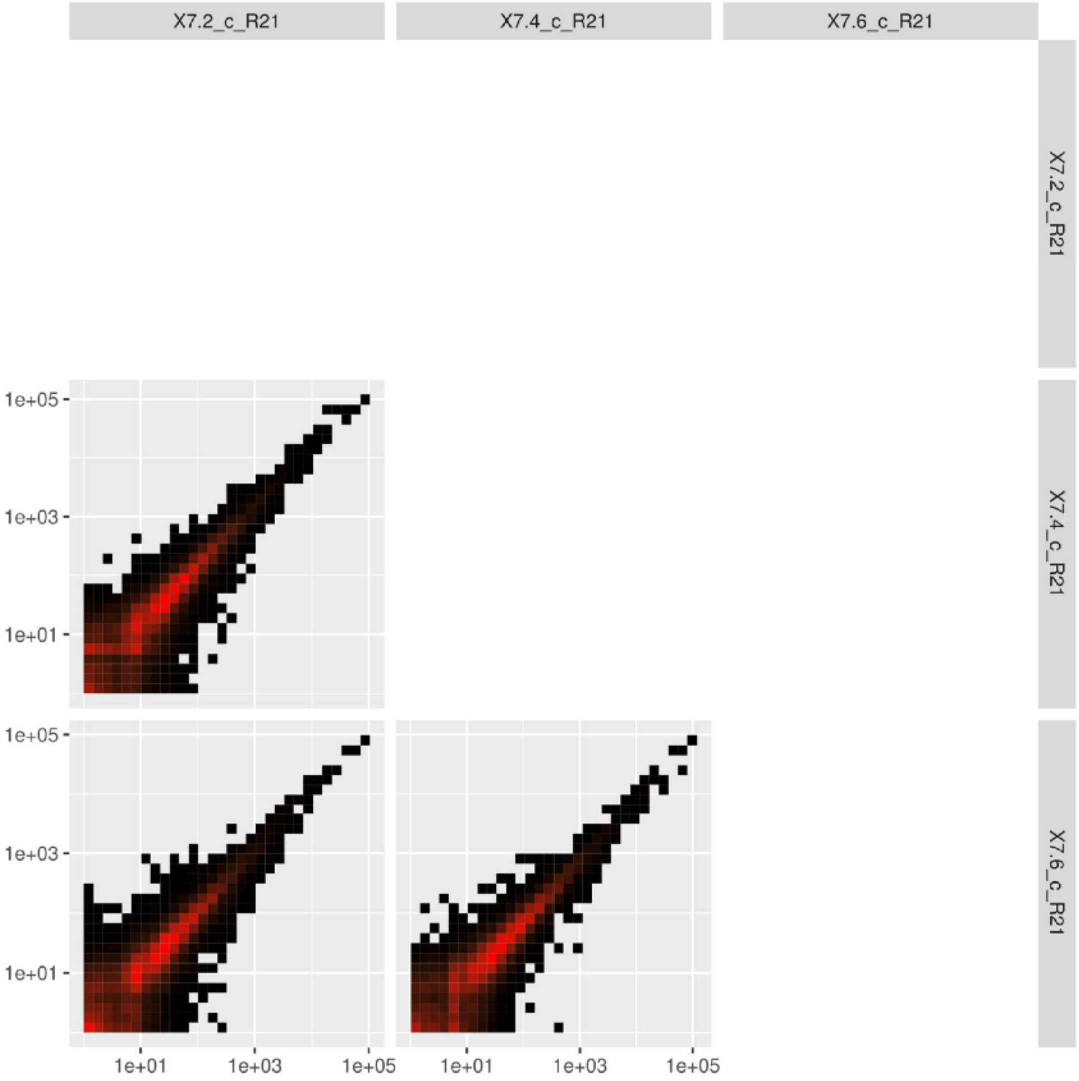
X7.2_c_R21
X7.4_c_R21
X7.6_c_R21
X7.2_c_R21
X7.4_c_R21
X7.6_c_R21
1e+05
1e+03
1e+01
1e+05
1e+03
1e+01
1e+01
1e+03
1e+05
1e+01
1e+03
1e+05

e

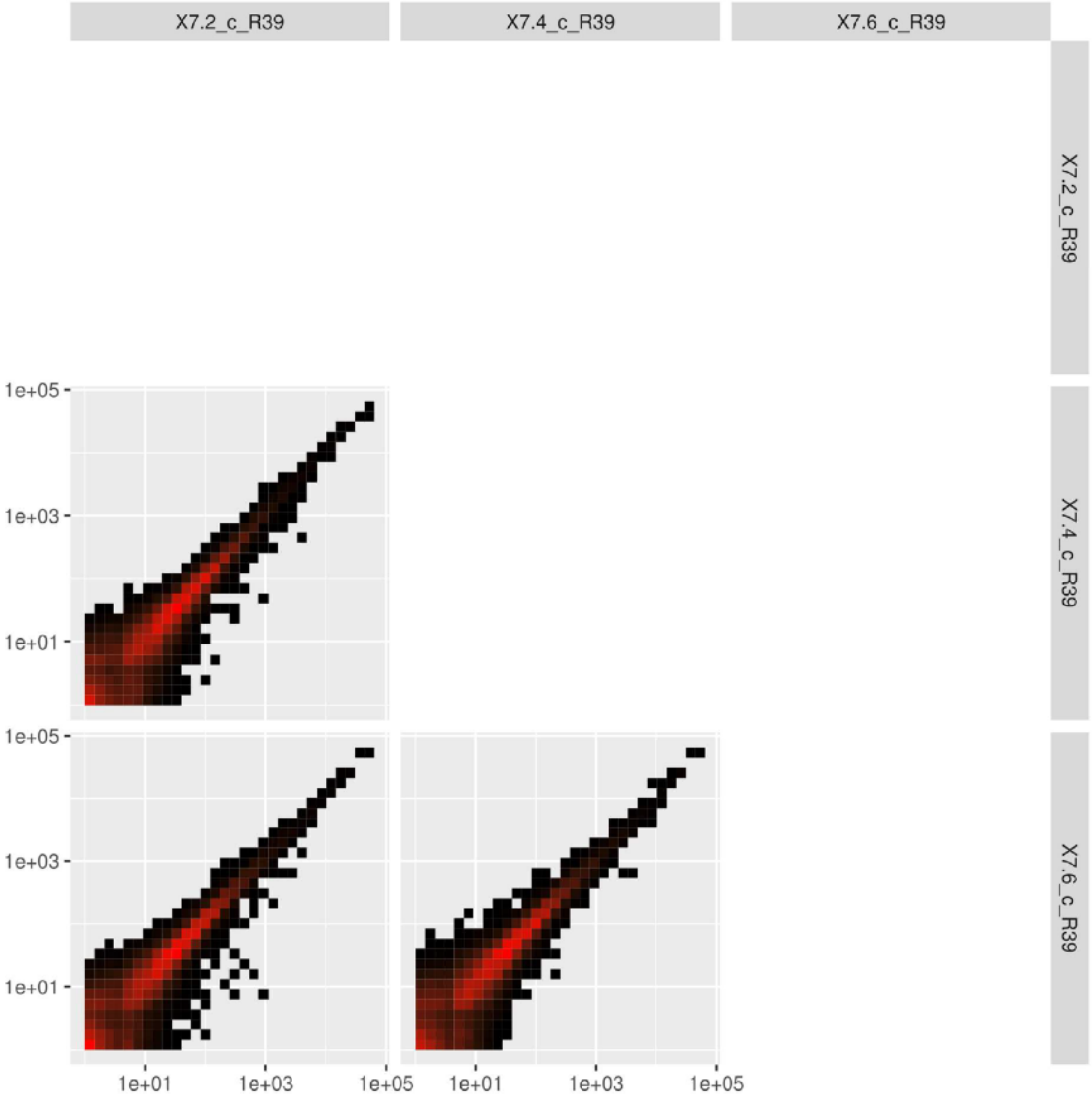

f

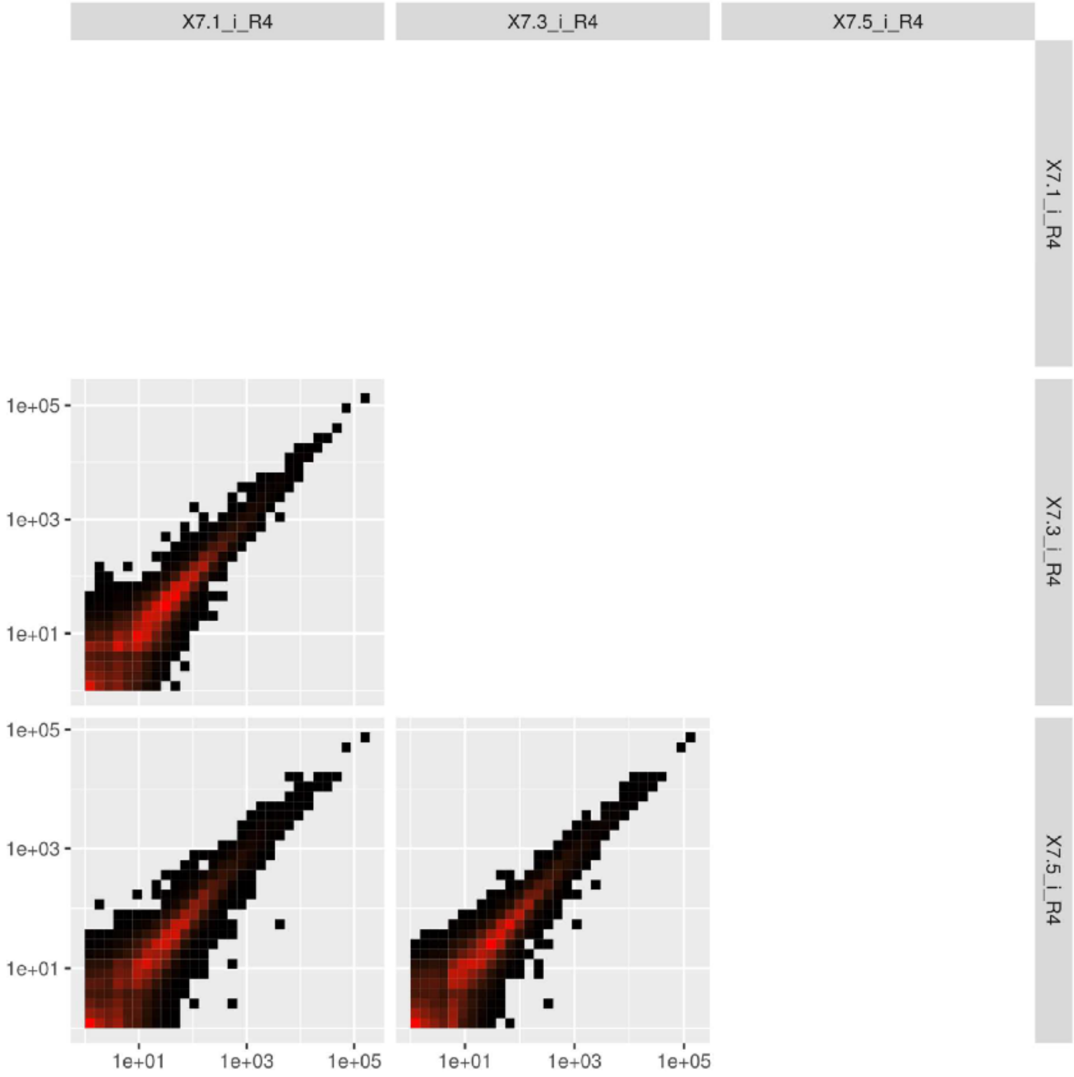
X7.1_i_R4
X7.3_i_R4
X7.5_i_R4
X7.1_i_R4
X7.3_i_R4
X7.5_i_R4
1e+05
1e+03
1e+01
1e+05
1e+03
1e+01
1e+01
1e+03
1e+05
1e+01
1e+03
1e+05

g

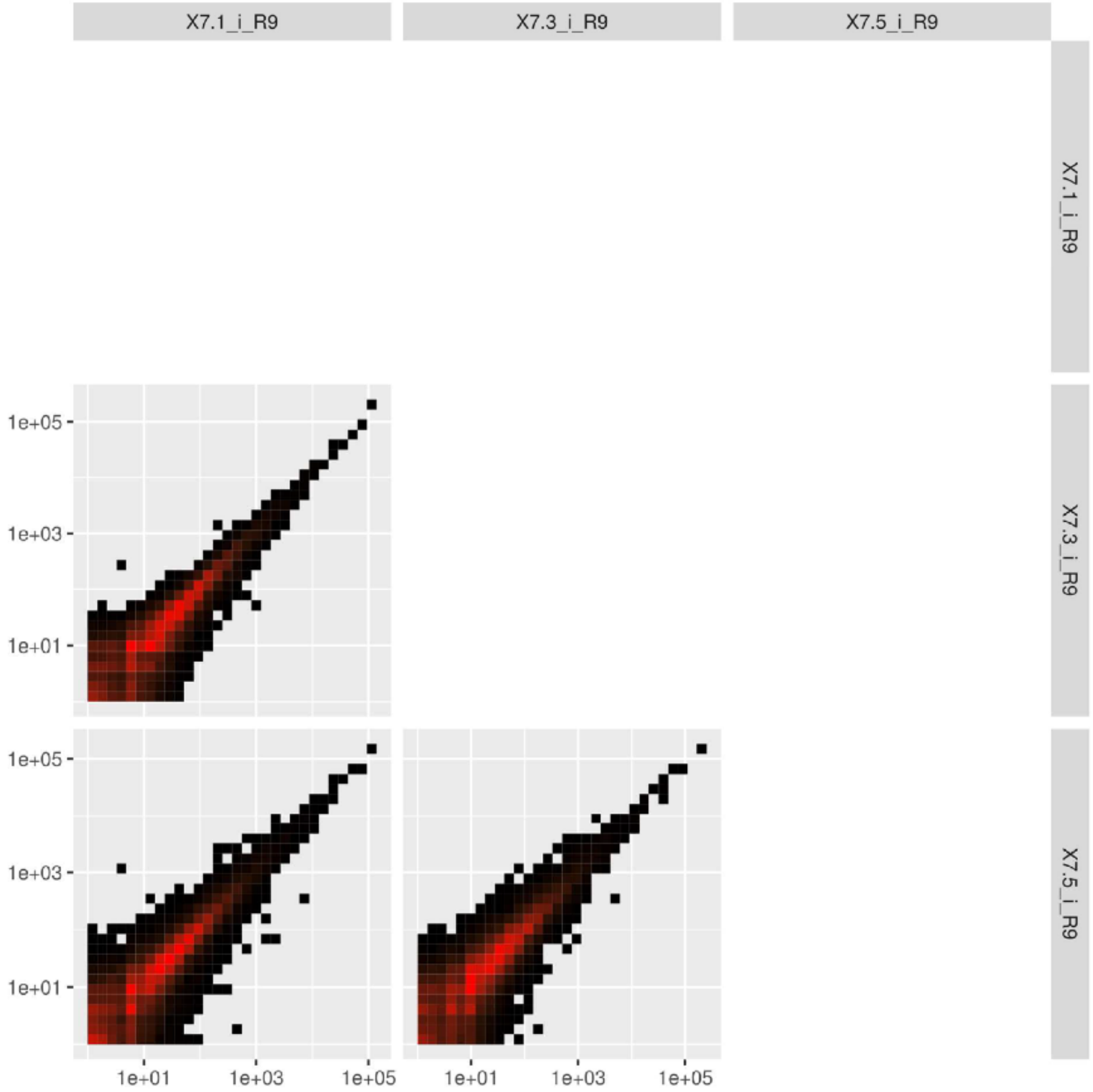
X7.1_i_R9
X7.3_i_R9
X7.5_i_R9
X7.1_i_R9
X7.3_i_R9
X7.5_i_R9
1e+05
1e+03
1e+01
1e+05
1e+03
1e+01
1e+01
1e+03
1e+05
1e+01
1e+03
1e+05

h

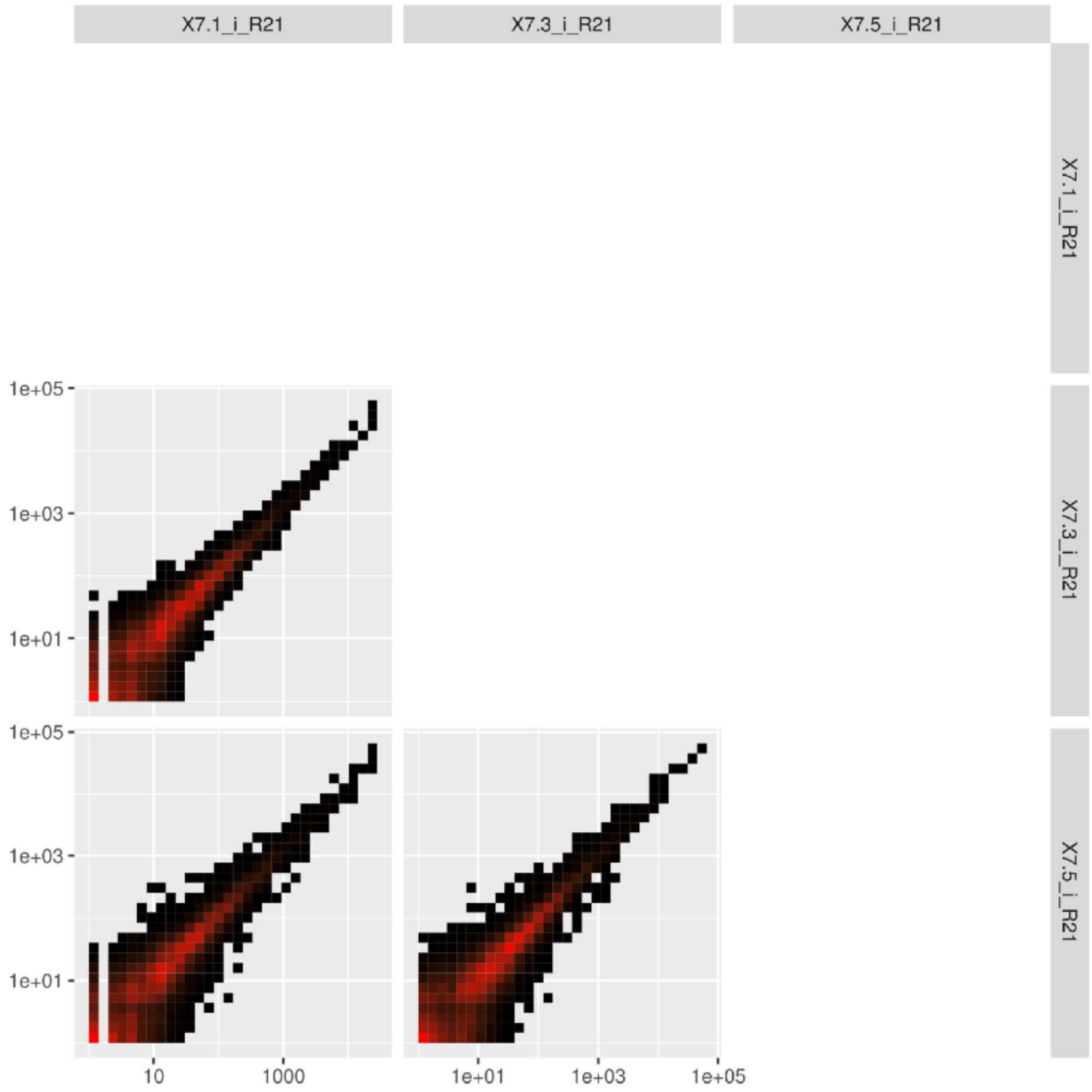

X7.1_i_R21
X7.3_i_R21
X7.5_i_R21
X7.1_i_R21
X7.3_i_R21
X7.5_i_R21
1e+05
1e+03
1e+01
1e+05
1e+03
1e+01
10
1000
1e+01
1e+03
1e+05

i

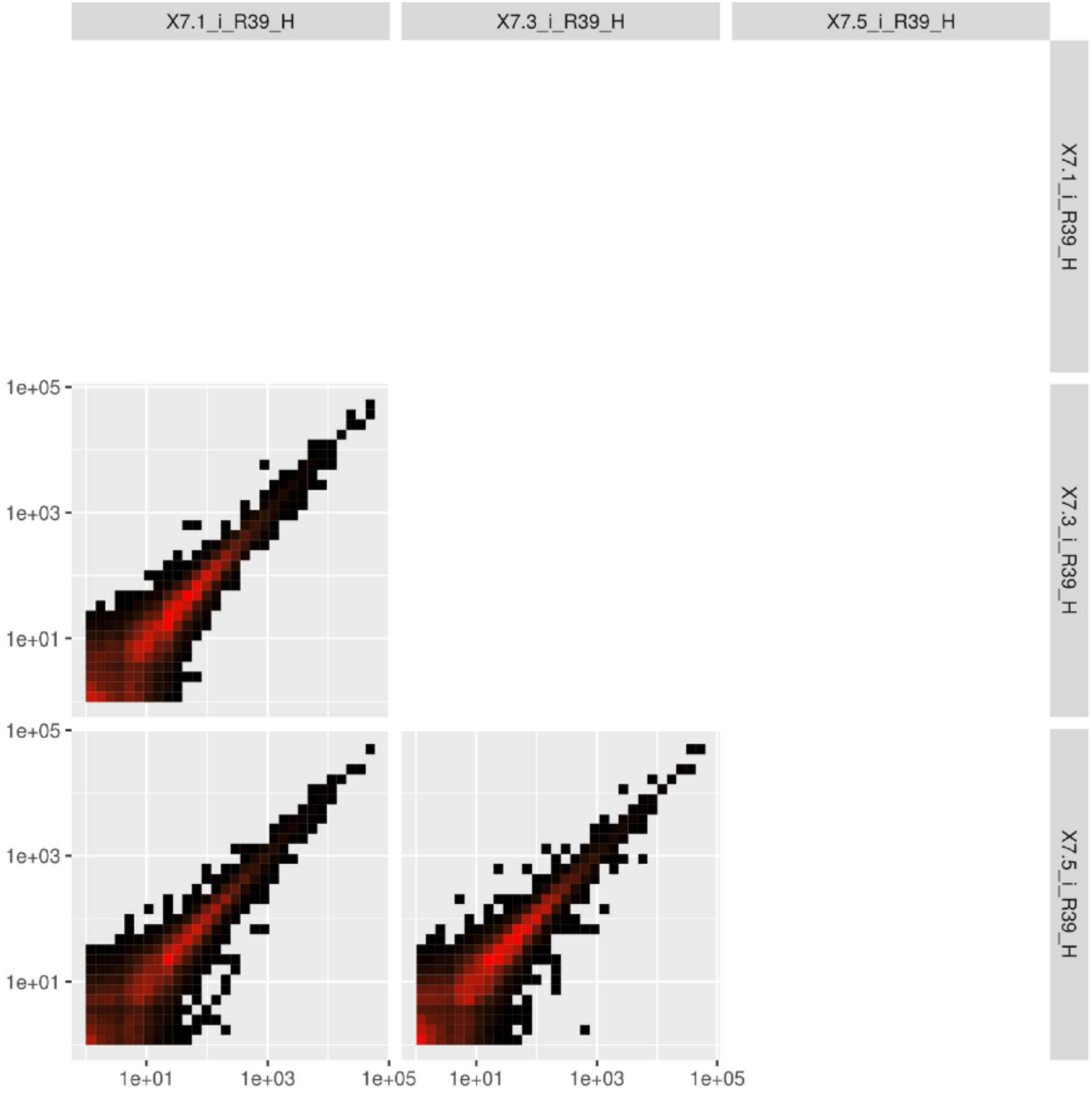

X7.1_i_R39_H
X7.3_i_R39_H
X7.5_i_R39_H
X7.1_i_R39_H
X7.3_i_R39_H
X7.5_i_R39_H
1e+05
1e+03
1e+01
1e+05
1e+03
1e+01
1e+01
1e+03
1e+05
1e+01
1e+03
1e+05

j

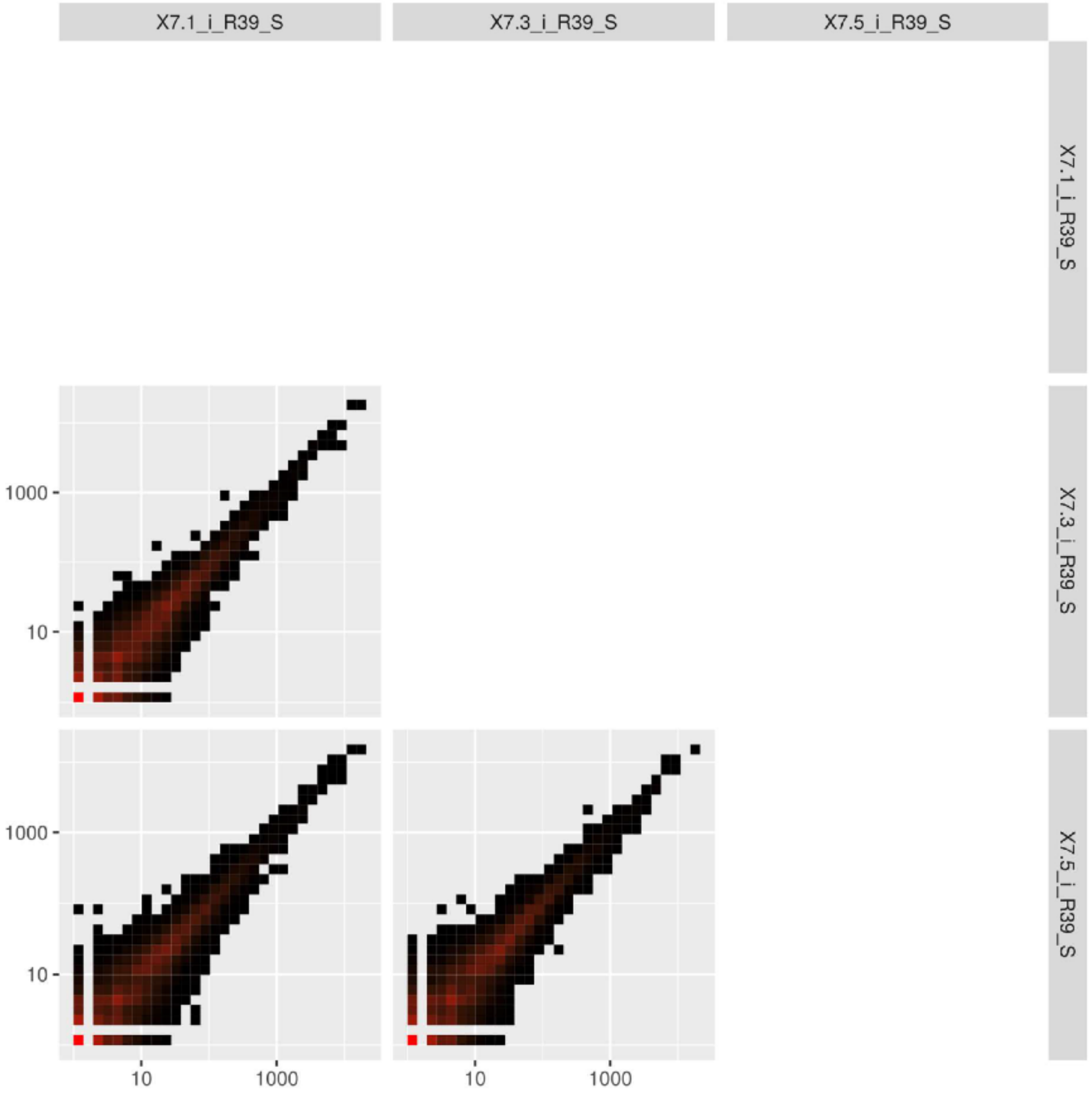

X7.1_i_R39_S
X7.3_i_R39_S
X7.5_i_R39_S
X7.1_i_R39_S
X7.3_i_R39_S
X7.5_i_R39_S
1000
10
1000
10
10
1000
10
1000

k

Sample1	Sample2	R_log
7.2_c_R1	7.4_c_R1	0.890562
7.2_c_R1	7.6_c_R1	0.881834
7.4_c_R1	7.6_c_R1	0.890782
7.2_c_R4	7.4_c_R4	0.869999
7.2_c_R4	7.6_c_R4	0.880093
7.4_c_R4	7.6_c_R4	0.898205
7.2_c_R9	7.4_c_R9	0.879975
7.2_c_R9	7.6_c_R9	0.885650
7.4_c_R9	7.6_c_R9	0.897163
7.2_c_R21	7.4_c_R21	0.865316
7.2_c_R21	7.6_c_R21	0.866502
7.4_c_R21	7.6_c_R21	0.881011
7.2_c_R39	7.4_c_R39	0.894217
7.2_c_R39	7.6_c_R39	0.900031
7.4_c_R39	7.6_c_R39	0.892715
7.1_i_R4	7.3_i_R4	0.884719
7.1_i_R4	7.5_i_R4	0.882345
7.3_i_R4	7.5_i_R4	0.893090
7.1_i_R9	7.3_i_R9	0.862172
7.1_i_R9	7.5_i_R9	0.862338
7.3_i_R9	7.5_i_R9	0.840485
7.1_i_R21	7.3_i_R21	0.882457
7.1_i_R21	7.5_i_R21	0.875458
7.3_i_R21	7.5_i_R21	0.875587
7.1_i_R39_H	7.3_i_R39_H	0.894651
7.1_i_R39_H	7.5_i_R39_H	0.890544
7.3_i_R39_H	7.5_i_R39_H	0.892327
7.1_i_R39_S	7.3_i_R39_S	0.889329
7.1_i_R39_S	7.5_i_R39_S	0.889856
7.3_i_R39_S	7.5_i_R39_S	0.886331
