# Supplementary figures and images for: RNA-Seq and secondary metabolite analyses reveal a putative defence-transcriptome in Norway spruce (Picea abies) against needle bladder rust (Chrysomyxa rhododendri) infection
Source: BMC Genomics. 2020 May 1;21:336. doi: 10.1186/s12864-020-6587-z (PMC7195740; doi:10.1186/s12864-020-6587-z)

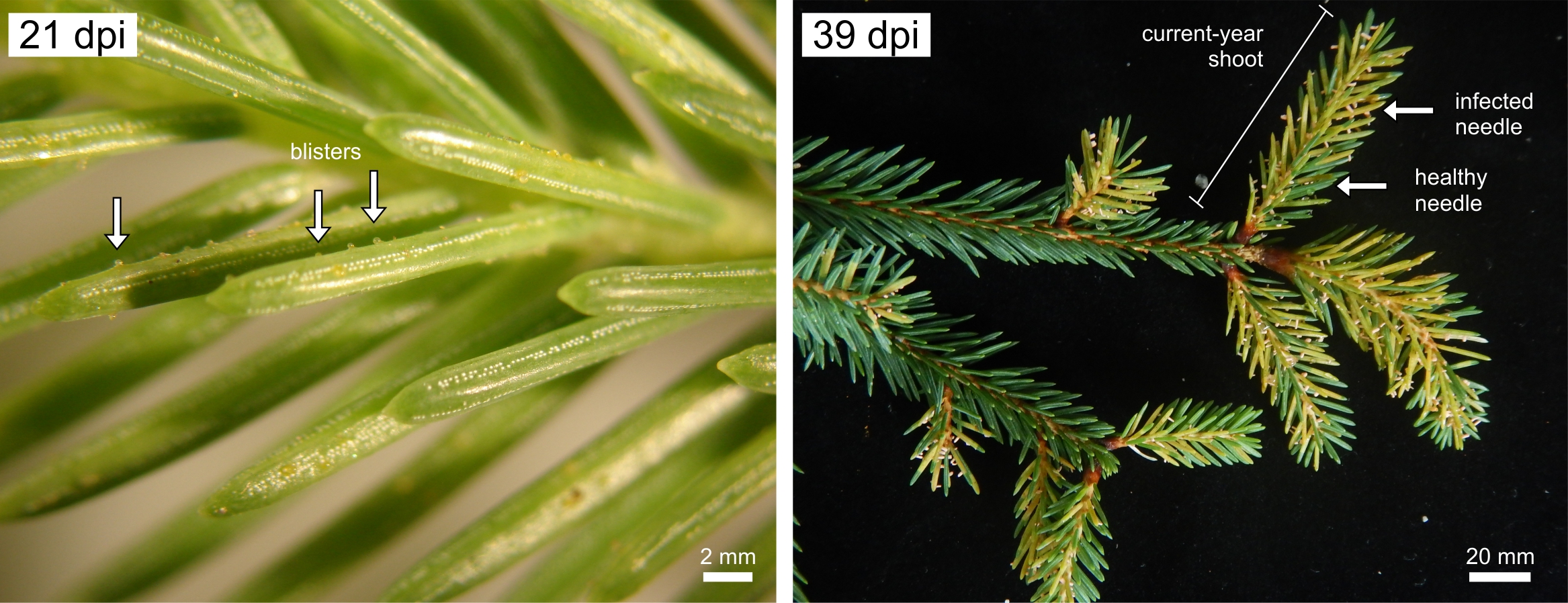

Supplement: Supplementary file 2 — Additional file 2: Figure S2. Symptoms of C. rhododendri infection at 21 and 39 dpi. At 21 dpi, first symptoms of infection were detectable under the microscope, i.e. small blisters on the needle surface become visible (see arrows). At 39 dpi, several current-year-needles of infected trees showed the characteristic yellow discoloration and first aecio spore stocks were formed. [file 12864_2020_6587_MOESM2_ESM.jpg]

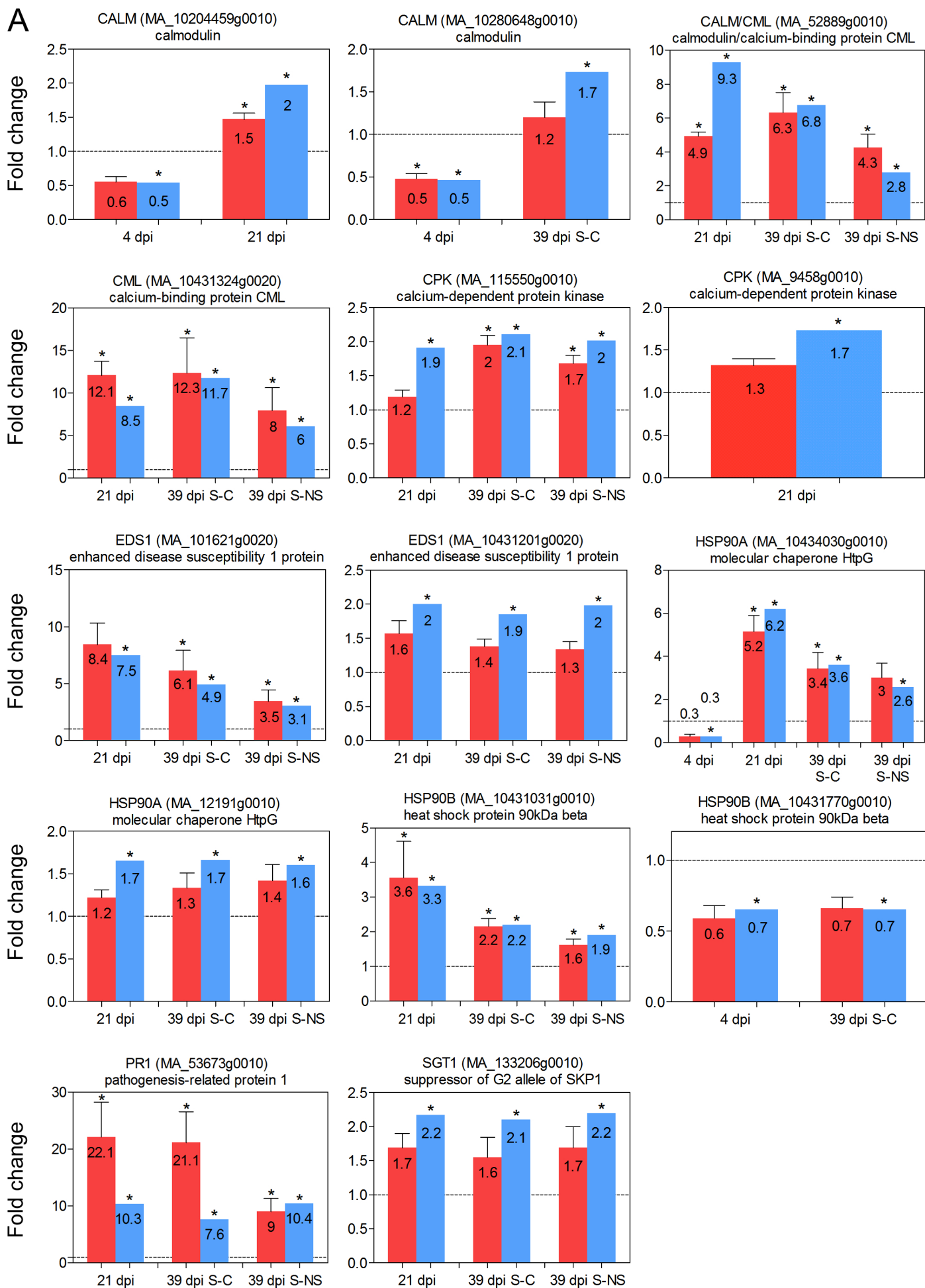

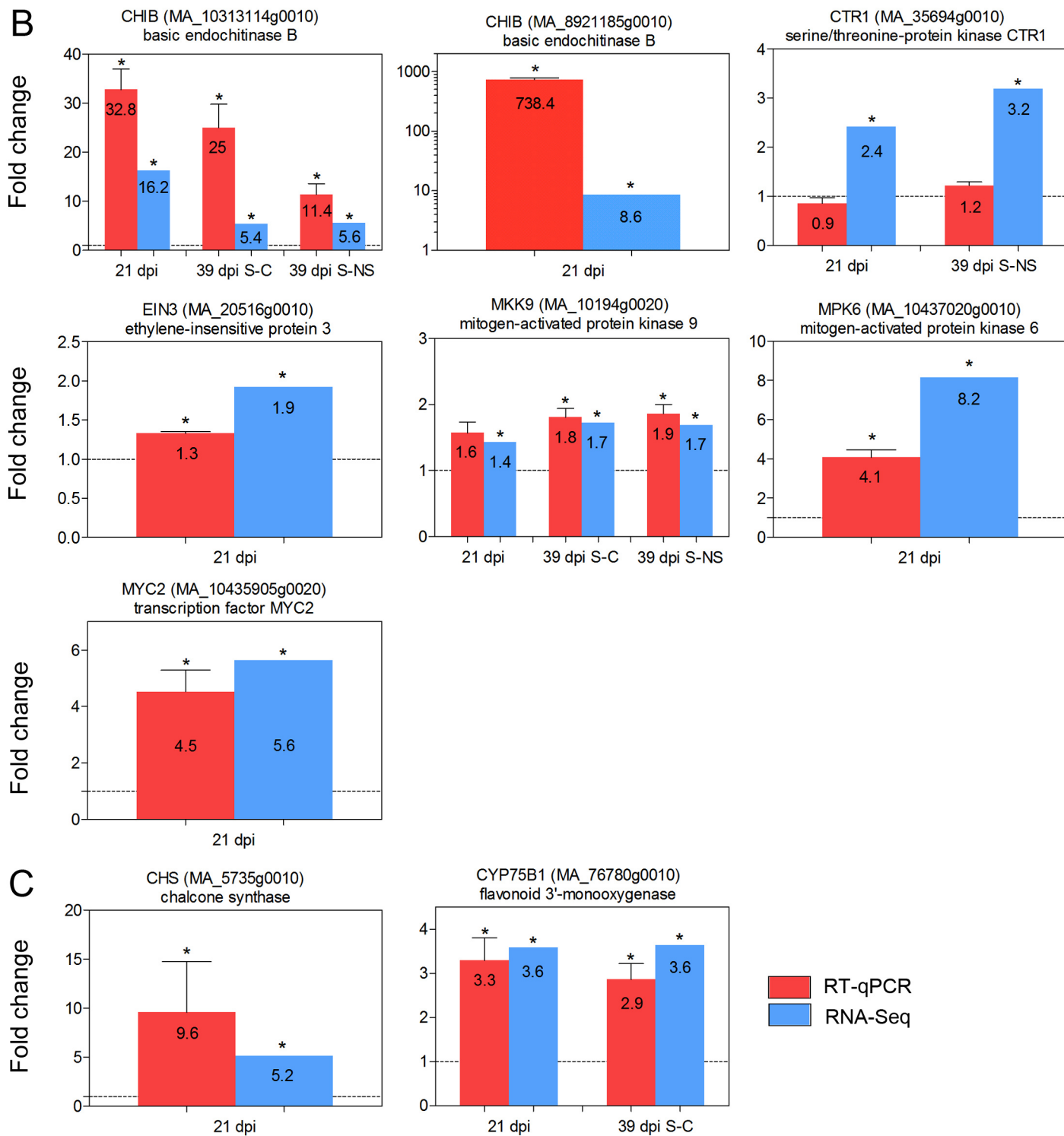

Supplement: Supplementary file 10 — Additional file 10: Figure S4. RT-qPCR validation of selected DEGs. Relative expression changes obtained by RT-qPCR (red bars; mean fold changes ± SE, n = 3) and RNA-seq (blue bars) compared to control plants at 4 dpi, 9 dpi, 21 dpi and 39 dpi (S-C). Symptomatic needles at 39 dpi were additionally compared to non-symptomatic needles (S-NS). Asterisk means significant (p < 0.05) difference compared to non-infected needles. [file 12864_2020_6587_MOESM10_ESM.pdf]

## STILBENES

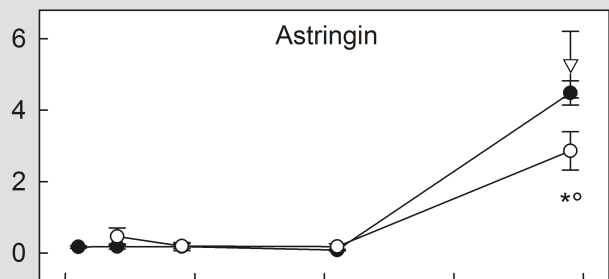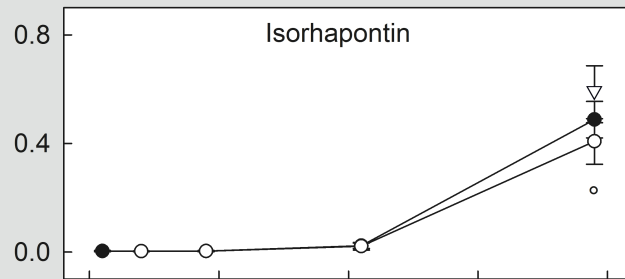

## FLAVONOIDS

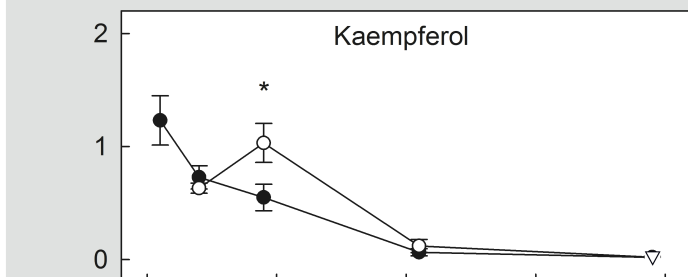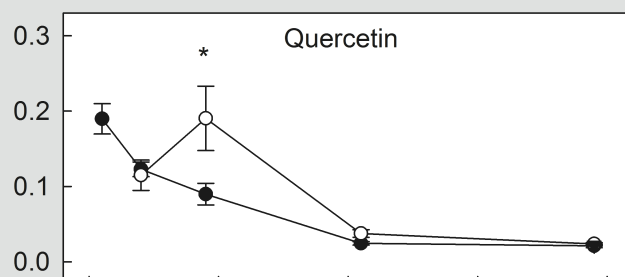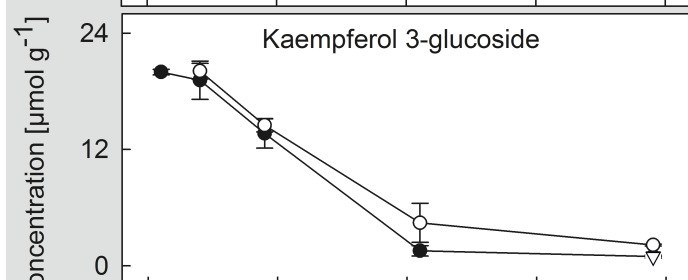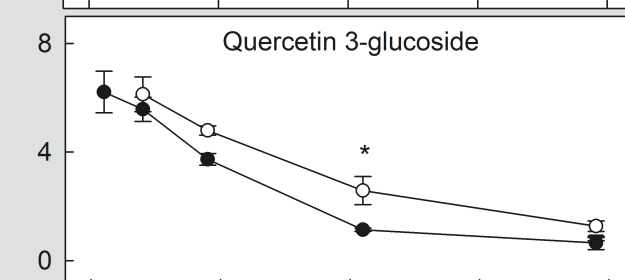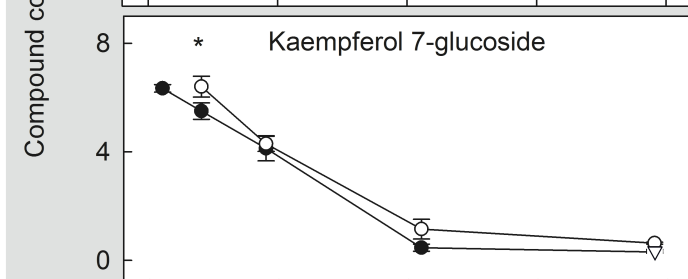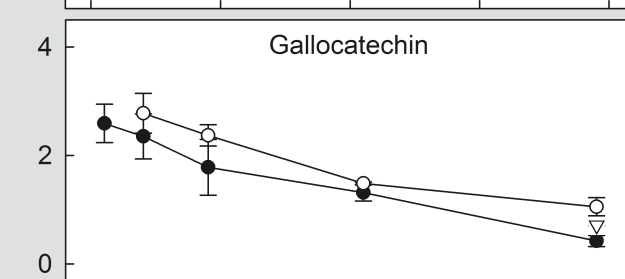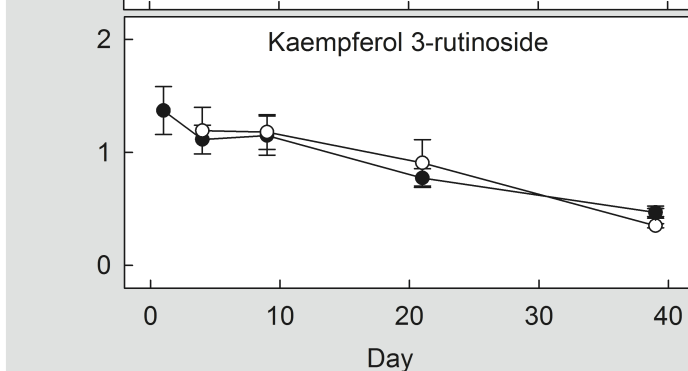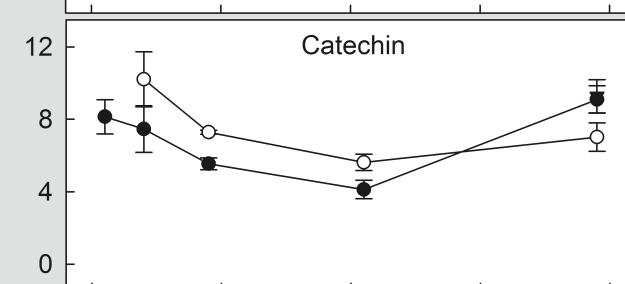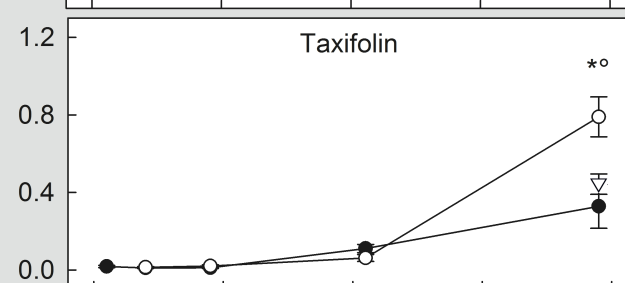

## SIMPLE PHENYLPROPANOID

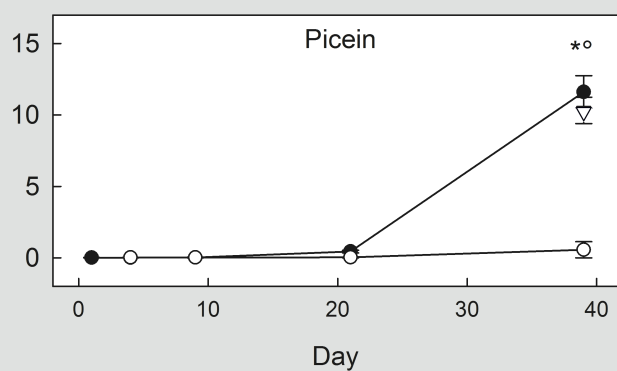

Supplement: Supplementary file 11 — Additional file 11: Figure S5. Concentration changes of all phenolic needle metabolites. Concentration changes of individual phenolic compounds during the experiment in needles of control (filled symbols, mean ± SE, n = 3) and spore exposed spruce cuttings (open symbols, mean ± SE, n = 3). Triangles on day 39 indicate concentrations in healthy needles of treated cuttings (mean ± SE, n = 3). Concentration values are given as μmol g− 1 dry weight; compounds with needle concentrations below the quantification threshold (naringenin, quercitrin, piceid, piceatannol, resveratrol, chlorogenic acid, gallic acid) are not shown. Significant differences between the control and treated group are indicated with asterisks and between healthy and symptomized needles of treated cuttings on day 39 with a circle. [file 12864_2020_6587_MOESM11_ESM.pdf]
