# Supplementary material for: RNA-Seq and secondary metabolite analyses reveal a putative defence-transcriptome in Norway spruce (Picea abies) against needle bladder rust (Chrysomyxa rhododendri) infection
Source: BMC Genomics. 2020 May 1;21:336. doi: 10.1186/s12864-020-6587-z (PMC7195740; doi:10.1186/s12864-020-6587-z)
Supplement: Supplementary file 8 — Additional file 8: Figure S3. KEGG pathways related to plant defence. A) Plant-pathogen interaction, B) MAPK signaling pathway-Plant, C) Plant hormone signal transduction, D) Phenylalanine, tyrosine and tryptophan biosynthesis, E) Phenylpropanoid biosynthesis, F) Stilbenoid, diarylheptanoid and gingerol biosynthesis, G) Flavonoid biosynthesis, H) Flavone and flavonol biosynthesis, I) Terpenoid backbone biosynthesis, J) Cutin, suberine and wax biosynthesis. At 39 dpi symptomatic needles were compared to controls. [file 12864_2020_6587_MOESM8_ESM.pdf]

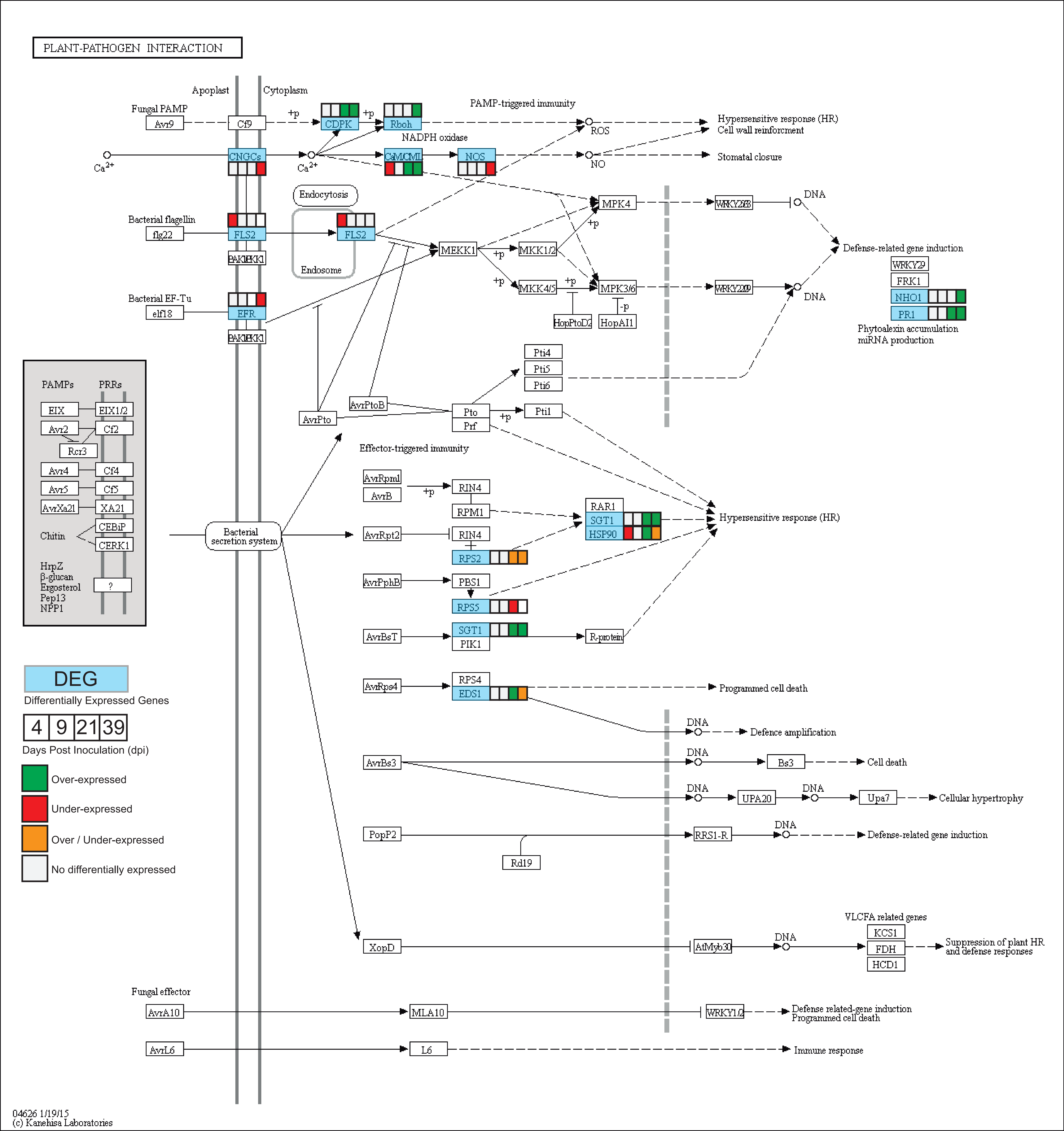

PLANT-PATHOGEN INTERACTION
Apoplast
Cytoplasm
Fungal PAMP
Avr9
Cf9
CDPK
Rboh
NADPH oxidase
PAMP-triggered immunity
ROS
Hypersensitive response (HR)
Cell wall reinforcment
Ca2+
CNGCs
CaM/CML
NOS
NO
Stomatal closure
Endocytosis
Endosome
Bacterial flagellin
flg22
FLS2
BAK1/BKK1
MEKK1
MKK1/2
MPK4
WRKY25/33
DNA
Defense-related gene induction
MKK4/5
MPK3/6
WRKY22/29
HopPtoD2
HopAI1
WRKY29
FRK1
NHO1
PR1
Phytoalexin accumulation
miRNA production
Bacterial EF-Tu
elf18
EFR
PAMPs
PRRs
EIX
EIX1/2
Avr2
Cf2
Rcr3
Avr4
Cf4
Avr5
Cf5
AvrXa21
XA21
Chitin
CEBiP
CERK1
HrpZ
β-glucan
Ergosterol
Pep13
NPP1
?
Pti4
Pti5
Pti6
AvrPtoB
AvrPto
Pto
Prf
Pti1
Effector-triggered immunity
Bacterial secretion system
AvrRpm1
AvrB
RIN4
RPM1
RAR1
SGT1
HSP90
AvrRpt2
RPS2
AvrPphB
PBS1
RPS5
AvrBsT
PIK1
R-protein
AvrRps4
RPS4
EDS1
Programmed cell death
Defence amplification
AvrBs3
Bs3
Cell death
UPA20
Upa7
Cellular hypertrophy
PopP2
RRS1-R
Rd19
XopD
AtMyb30
VLCFA related genes
KCS1
FDH
HCD1
Suppression of plant HR and defense responses
Fungal effector
AvrA10
MLA10
WRKY1/2
Defense related-gene induction
Programmed cell death
AvrL6
L6
Immune response
DEG
Differentially Expressed Genes
4 9 21 39
Days Post Inoculation (dpi)
Over-expressed
Under-expressed
Over / Under-expressed
No differentially expressed
04626 1/19/15
(c) Kanehisa Laboratories

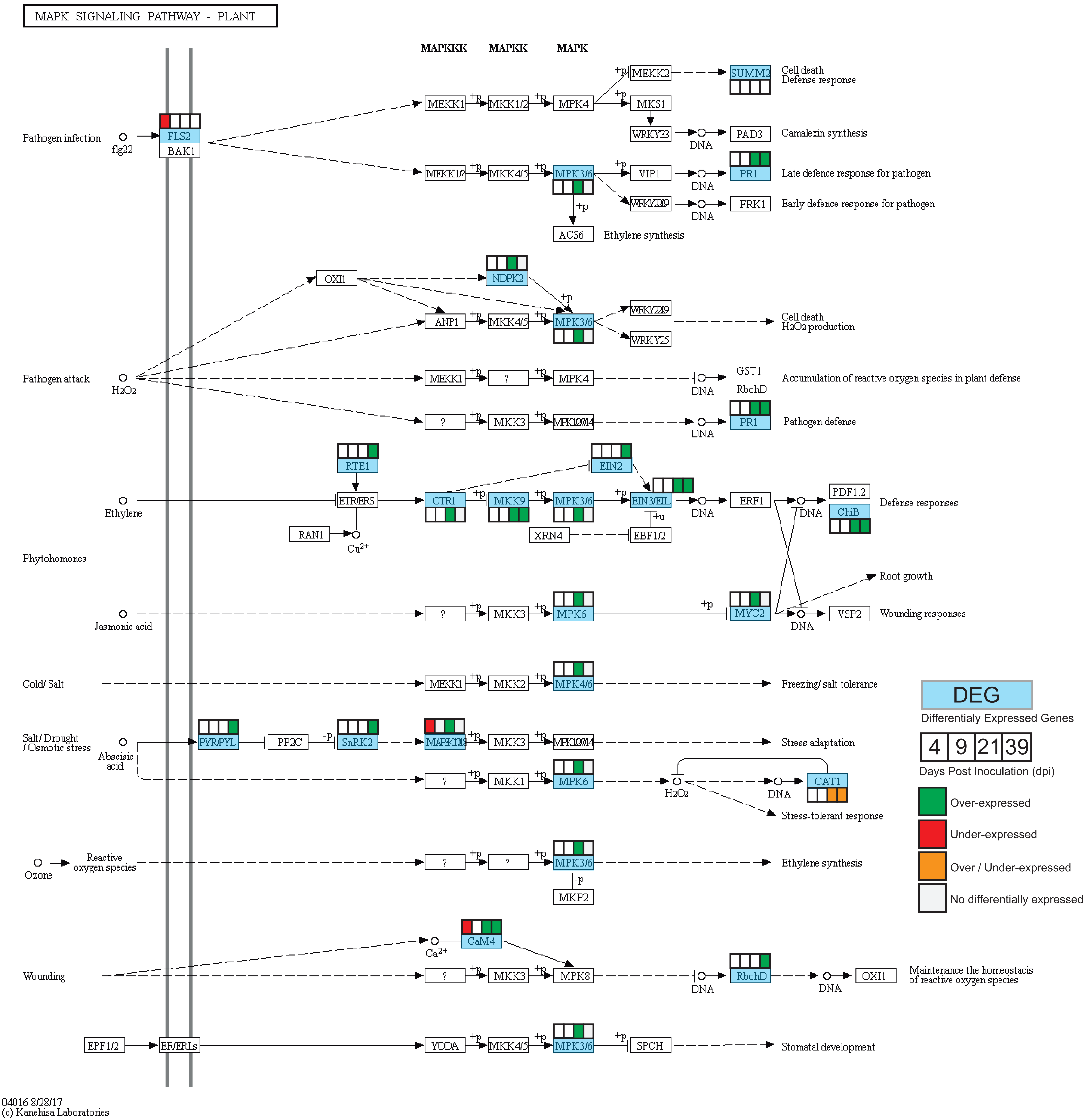

MAPK SIGNALING PATHWAY - PLANT
MAPKKK
MAPKK
MAPK
Pathogen infection
flg22
FLS2
BAK1
MEKK1
MKK1/2
MPK4
MEKK2
SUMM2
Cell death
Defense response
MKS1
WRKY33
DNA
PAD3
Camalexin synthesis
MKK4/5
MPK3/6
VIP1
PR1
Late defence response for pathogen
WRKY22/29
FRK1
Early defence response for pathogen
ACS6
Ethylene synthesis
OXI1
NDPK2
ANP1
WRKY22/29
WRKY25
Cell death
H2O2 production
Pathogen attack
H2O2
?
GST1
RbohD
Accumulation of reactive oxygen species in plant defense
MKK3
MPK1/2/7/14
Pathogen defense
RTE1
EIN2
Ethylene
ETR/ERS
CTR1
MKK9
EIN3/EIL
ERF1
PDF1.2
ChiB
Defense responses
RAN1
Cu2+
XRN4
EBF1/2
Phytohomones
Root growth
Jasmonic acid
MPK6
MYC2
VSP2
Wounding responses
Cold/ Salt
MKK2
MPK4/6
Freezing/ salt tolerance
DEG
Differentialy Expressed Genes
4 9 21 39
Days Post Inoculation (dpi)
Over-expressed
Under-expressed
Over / Under-expressed
No differentially expressed
Salt/ Drought
/ Osmotic stress
Abscisic acid
PYR/PYL
PP2C
SnRK2
MAPKKK17/18
Stress adaptation
MKK1
CAT1
Stress-tolerant response
Ozone
Reactive oxygen species
MKP2
Ethylene synthesis
CaM4
Ca2+
Wounding
MPK8
OXI1
Maintenance the homeostacis of reactive oxygen species
EPF1/2
ER/ERLs
YODA
SPCH
Stomatal development
04016 8/28/17
(c) Kanehisa Laboratories

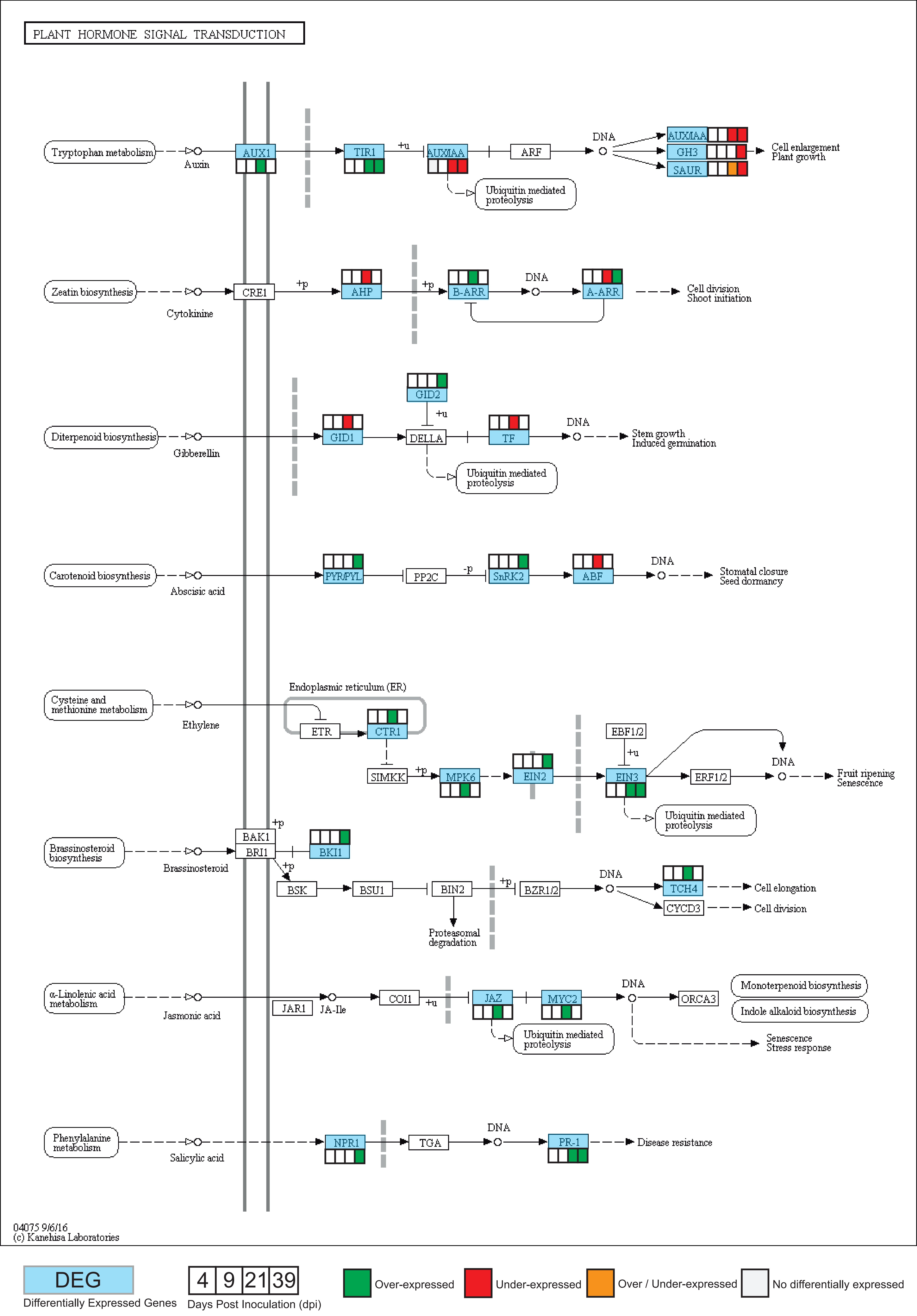
PLANT HORMONE SIGNAL TRANSDUCTION
Tryptophan metabolism
Auxin
AUX1
TIR1
+u
AUX/IAA
ARF
DNA
AUX/IAA
GH3
SAUR
Cell enlargement
Plant growth
Ubiquitin mediated proteolysis
Zeatin biosynthesis
Cytokinine
CRE1
+p
AHP
+p
B-ARR
DNA
A-ARR
Cell division
Shoot initiation
GID2
+u
Diterpenoid biosynthesis
Gibberellin
GID1
DELLA
TF
DNA
Stem growth
Induced germination
Ubiquitin mediated proteolysis
Carotenoid biosynthesis
Abscisic acid
PYR/PYL
PP2C
-p
SnRK2
ABF
DNA
Stomatal closure
Seed dormancy
Endoplasmic reticulum (ER)
Cysteine and methionine metabolism
Ethylene
ETR
CTR1
SIMKK
+p
MPK6
EIN2
EBF1/2
+u
EIN3
ERF1/2
DNA
Fruit ripening
Senescence
Ubiquitin mediated proteolysis
Brassinosteroid biosynthesis
Brassinosteroid
BAK1
BRI1
+p
BKI1
+p
BSK
BSU1
BIN2
+p
BZR1/2
DNA
TCH4
CYCD3
Cell elongation
Cell division
Proteasomal degradation
α-Linolenic acid metabolism
Jasmonic acid
JAR1
JA-Ile
COI1
+u
JAZ
MYC2
DNA
ORCA3
Monoterpenoid biosynthesis
Indole alkaloid biosynthesis
Ubiquitin mediated proteolysis
Senescence
Stress response
Phenylalanine metabolism
Salicylic acid
NPR1
TGA
DNA
PR-1
Disease resistance
04075 9/6/16
(c) Kanehisa Laboratories
DEG
Differentially Expressed Genes
4 9 21 39
Days Post Inoculation (dpi)
Over-expressed
Under-expressed
Over / Under-expressed
No differentially expressed

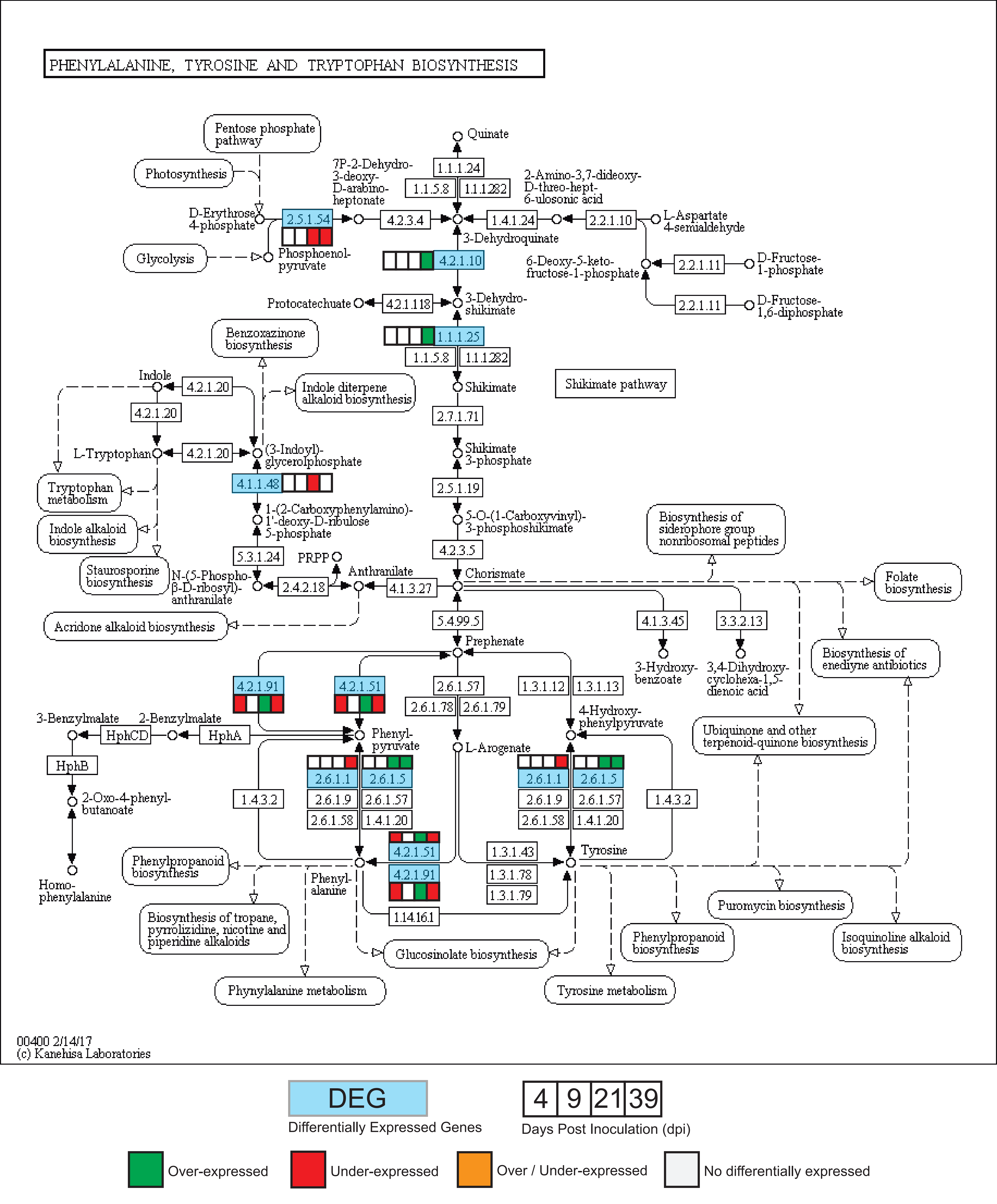
PHENYLALANINE, TYROSINE AND TRYPTOPHAN BIOSYNTHESIS
Pentose phosphate pathway
Photosynthesis
Glycolysis
D-Erythrose 4-phosphate
2.5.1.54
Phosphoenol-pyruvate
7P-2-Dehydro-3-deoxy-D-arabino-heptonate
4.2.3.4
Quinate
1.1.1.24
1.1.5.8
1.1.1.282
3-Dehydroquinate
4.2.1.10
1.4.1.24
2-Amino-3,7-dideoxy-D-threo-hept-6-ulosonic acid
2.2.1.10
L-Aspartate 4-semialdehyde
6-Deoxy-5-keto-fructose-1-phosphate
2.2.1.11
D-Fructose-1-phosphate
D-Fructose-1,6-diphosphate
Protocatechuate
4.2.1.118
3-Dehydro-shikimate
1.1.1.25
Shikimate
Shikimate pathway
2.7.1.71
Shikimate 3-phosphate
2.5.1.19
5-O-(1-Carboxyvinyl)-3-phosphoshikimate
4.2.3.5
Chorismate
Benzoxazinone biosynthesis
Indole diterpene alkaloid biosynthesis
Indole
4.2.1.20
L-Tryptophan
(3-Indoyl)-glycerolphosphate
4.1.1.48
1-(2-Carboxyphenylamino)-1'-deoxy-D-ribulose 5-phosphate
5.3.1.24
Tryptophan metabolism
Indole alkaloid biosynthesis
Staurosporine biosynthesis
N-(5-Phospho-β-D-ribosyl)-anthranilate
2.4.2.18
PRPP
Anthranilate
4.1.3.27
Acridone alkaloid biosynthesis
Biosynthesis of siderophore group nonribosomal peptides
Folate biosynthesis
5.4.99.5
Prephenate
4.1.3.45
3.3.2.13
3-Hydroxy-benzoate
3,4-Dihydroxy-cyclohexa-1,5-dienoic acid
Biosynthesis of enediyne antibiotics
4.2.1.91
4.2.1.51
2.6.1.57
2.6.1.78
2.6.1.79
1.3.1.12
1.3.1.13
4-Hydroxy-phenylpyruvate
3-Benzylmalate
2-Benzylmalate
HphCD
HphA
HphB
Phenyl-pyruvate
L-Arogenate
Ubiquinone and other terpenoid-quinone biosynthesis
2-Oxo-4-phenyl-butanoate
1.4.3.2
2.6.1.1
2.6.1.5
2.6.1.9
2.6.1.58
1.4.1.20
Homo-phenylalanine
Phenylpropanoid biosynthesis
Phenyl-alanine
1.3.1.43
1.3.1.78
1.3.1.79
Tyrosine
1.14.16.1
Biosynthesis of tropane, pyrrolizidine, nicotine and piperidine alkaloids
Glucosinolate biosynthesis
Puromycin biosynthesis
Isoquinoline alkaloid biosynthesis
Phynylalanine metabolism
Tyrosine metabolism
00400 2/14/17
(c) Kanehisa Laboratories
DEG
Differentially Expressed Genes
4 9 21 39
Days Post Inoculation (dpi)
Over-expressed
Under-expressed
Over / Under-expressed
No differentially expressed

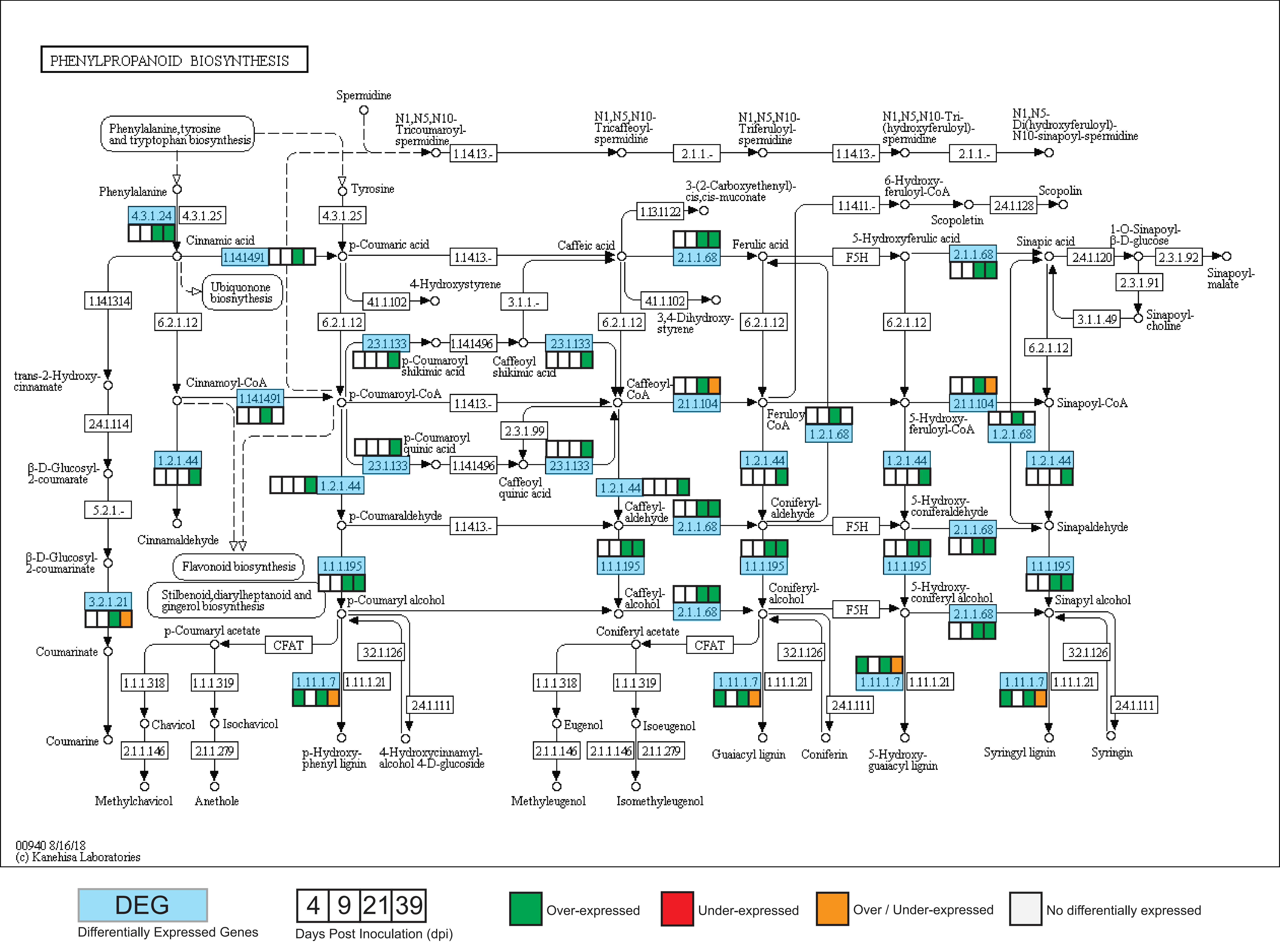
PHENYLPROPANOID BIOSYNTHESIS
Spermidine
N1,N5,N10-Tricoumaroyl-spermidine
N1,N5,N10-Tricaffeoyl-spermidine
N1,N5,N10-Triferuloyl-spermidine
N1,N5,N10-Tri-(hydroxyferuloyl)-spermidine
N1,N5-Di(hydroxyferuloyl)-N10-sinapoyl-spermidine
Phenylalanine,tyrosine and tryptophan biosynthesis
Phenylalanine
Tyrosine
4.3.1.24
4.3.1.25
Cinnamic acid
1.14.14.91
p-Coumaric acid
1.14.13.-
Caffeic acid
2.1.1.68
Ferulic acid
F5H
5-Hydroxyferulic acid
Sinapic acid
2.4.1.120
1-O-Sinapoyl-β-D-glucose
2.3.1.92
Sinapoyl-malate
2.3.1.91
3.1.1.49
Sinapoyl-choline
3-(2-Carboxyethenyl)-cis,cis-muconate
1.13.11.22
6-Hydroxy-feruloyl-CoA
1.14.11.-
Scopoletin
2.4.1.128
Scopolin
2.1.1.-
1.14.13.14
Ubiquinone biosynthesis
4-Hydroxystyrene
4.1.1.102
3.1.1.-
3,4-Dihydroxy-styrene
6.2.1.12
2.3.1.133
1.14.14.96
p-Coumaroyl shikimic acid
Caffeoyl shikimic acid
trans-2-Hydroxy-cinnamate
Cinnamoyl-CoA
p-Coumaroyl-CoA
Caffeoyl-CoA
2.1.1.104
Feruloyl CoA
5-Hydroxy-feruloyl-CoA
Sinapoyl-CoA
1.2.1.68
2.4.1.114
2.3.1.99
p-Coumaroyl quinic acid
Caffeoyl quinic acid
β-D-Glucosyl-2-coumarate
1.2.1.44
5.2.1.-
Cinnamaldehyde
p-Coumaraldehyde
Caffeyl-aldehyde
Coniferyl-aldehyde
5-Hydroxy-coniferaldehyde
Sinapaldehyde
β-D-Glucosyl-2-coumarinate
Flavonoid biosynthesis
1.1.1.195
3.2.1.21
Stilbenoid,diarylheptanoid and gingerol biosynthesis
p-Coumaryl alcohol
Caffeyl-alcohol
Coniferyl-alcohol
5-Hydroxy-coniferyl alcohol
Sinapyl alcohol
Coumarinate
p-Coumaryl acetate
CFAT
Coniferyl acetate
3.2.1.126
1.1.1.318
1.1.1.319
1.11.1.7
1.11.1.21
2.4.1.111
Chavicol
Isochavicol
Eugenol
Isoeugenol
Coumarine
2.1.1.146
2.1.1.279
p-Hydroxy-phenyl lignin
4-Hydroxycinnamyl-alcohol 4-D-glucoside
Guaiacyl lignin
Coniferin
5-Hydroxy-guaiacyl lignin
Syringyl lignin
Syringin
Methylchavicol
Anethole
Methyleugenol
Isomethyleugenol
00940 8/16/18
(c) Kanehisa Laboratories
DEG
Differentially Expressed Genes
4 9 21 39
Days Post Inoculation (dpi)
Over-expressed
Under-expressed
Over / Under-expressed
No differentially expressed

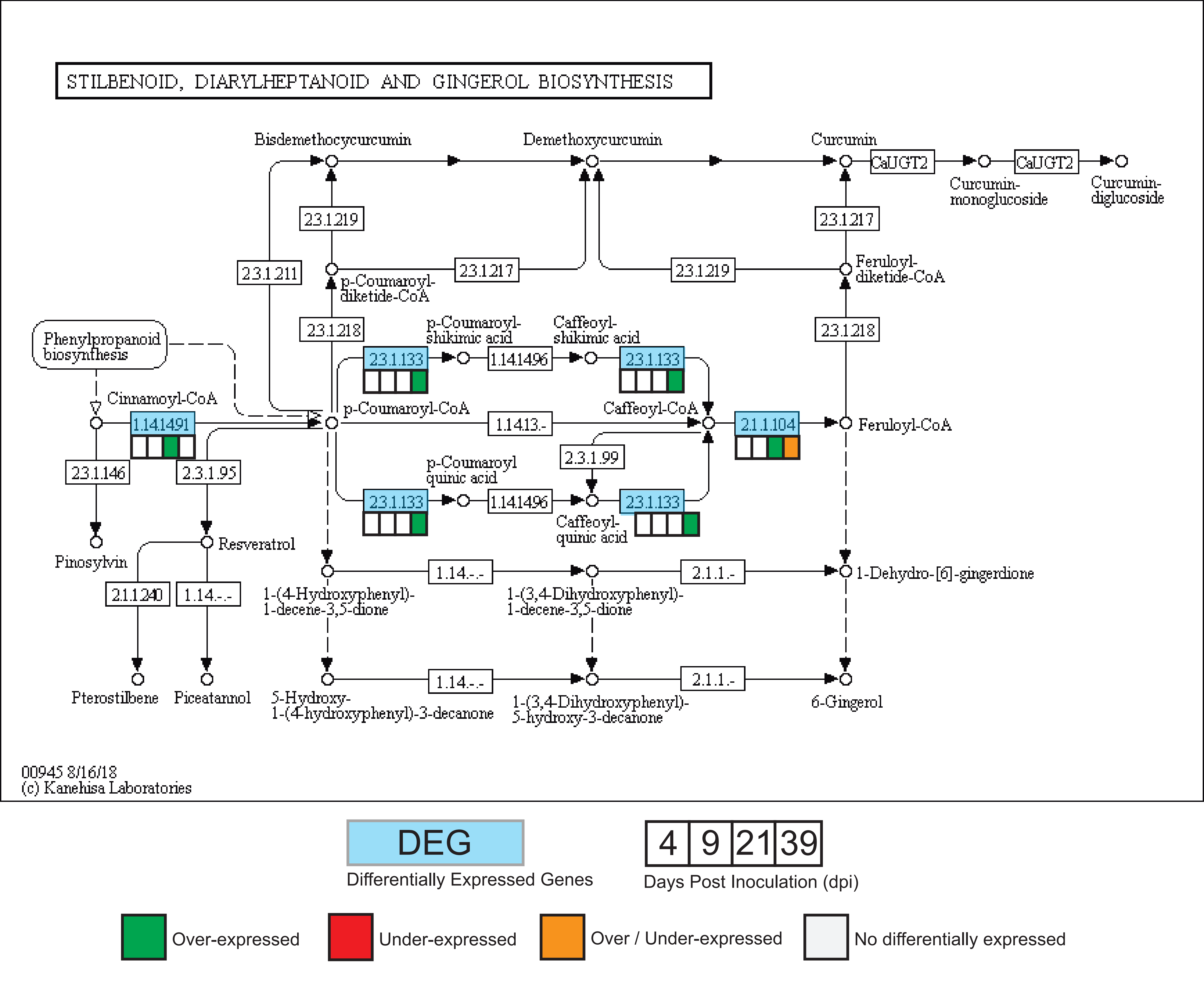

STILBENOID, DIARYLHEPTANOID AND GINGEROL BIOSYNTHESIS
Bisdemethoxycurcumin
Demethoxycurcumin
Curcumin
CaUGT2
CaUGT2
Curcumin-monoglucoside
Curcumin-diglucoside
2.3.1.219
2.3.1.217
2.3.1.211
2.3.1.217
2.3.1.219
p-Coumaroyl-diketide-CoA
Feruloyl-diketide-CoA
2.3.1.218
2.3.1.218
Phenylpropanoid biosynthesis
p-Coumaroyl-shikimic acid
Caffeoyl-shikimic acid
2.3.1.133
1.14.14.96
2.3.1.133
Cinnamoyl-CoA
p-Coumaroyl-CoA
Caffeoyl-CoA
1.14.14.91
1.14.13.-
2.1.1.104
Feruloyl-CoA
2.3.1.99
2.3.1.146
2.3.1.95
p-Coumaroyl quinic acid
2.3.1.133
1.14.14.96
2.3.1.133
Caffeoyl-quinic acid
Pinosylvin
Resveratrol
1.14.-.-
2.1.1.-
1-Dehydro-[6]-gingerdione
2.1.1.240
1.14.-.-
1-(4-Hydroxyphenyl)-1-decene-3,5-dione
1-(3,4-Dihydroxyphenyl)-1-decene-3,5-dione
1.14.-.-
2.1.1.-
Pterostilbene
Piceatannol
5-Hydroxy-1-(4-hydroxyphenyl)-3-decanone
1-(3,4-Dihydroxyphenyl)-5-hydroxy-3-decanone
6-Gingerol
00945 8/16/18
(c) Kanehisa Laboratories
DEG
Differentially Expressed Genes
4
9
21
39
Days Post Inoculation (dpi)
Over-expressed
Under-expressed
Over / Under-expressed
No differentially expressed

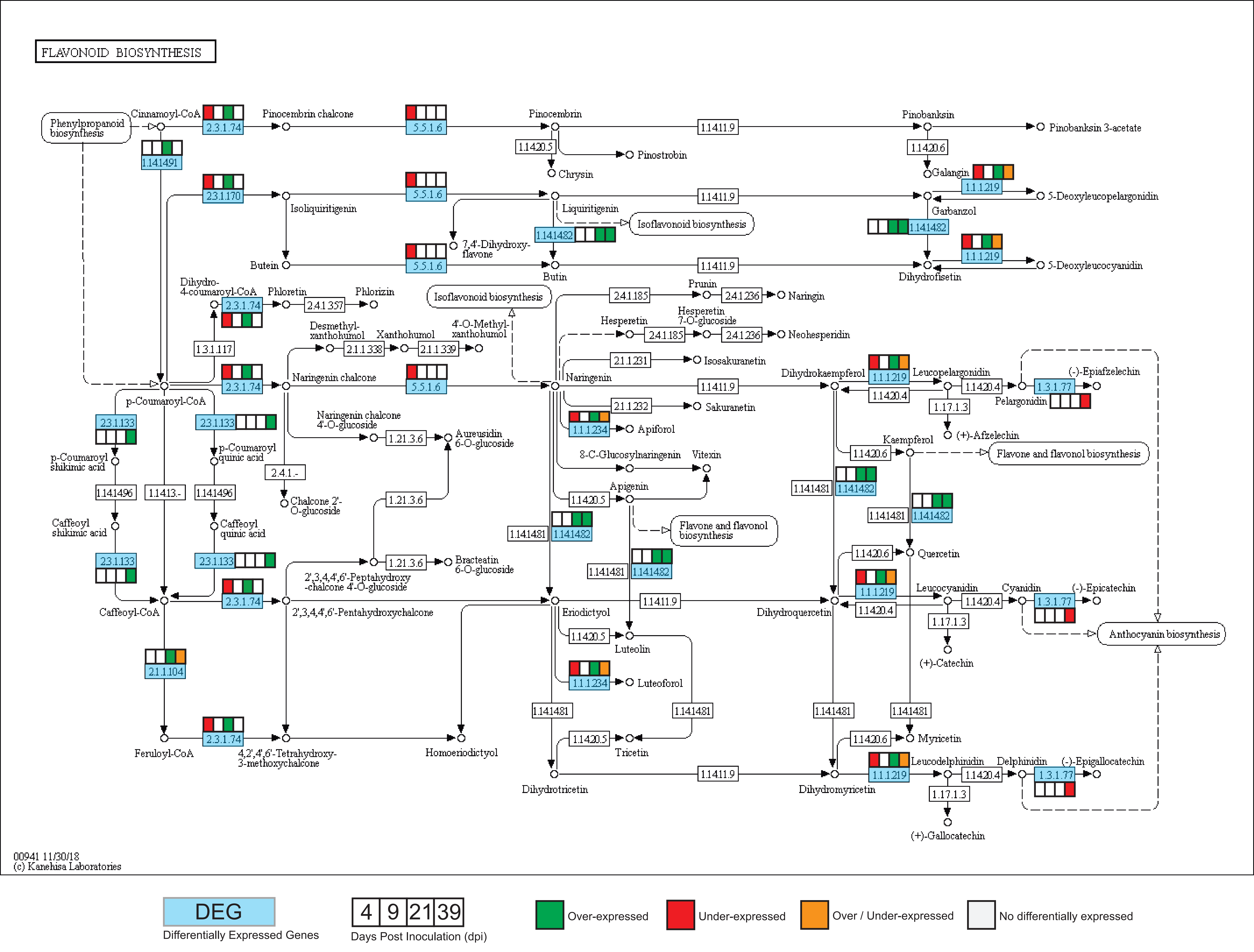
FLAVONOID BIOSYNTHESIS
Phenylpropanoid biosynthesis
Cinnamoyl-CoA
2.3.1.74
1.14.14.91
Pinocembrin chalcone
5.5.1.6
Pinocembrin
1.14.11.9
Pinobanksin
Pinobanksin 3-acetate
1.14.20.5
Pinostrobin
Chrysin
1.14.20.6
Galangin
1.1.1.219
5-Deoxyleucopelargonidin
2.3.1.170
Isoliquiritigenin
Liquiritigenin
Isoflavonoid biosynthesis
Garbanzol
1.14.14.82
7,4'-Dihydroxy-flavone
Butein
Butin
Dihydrofisetin
5-Deoxyleucocyanidin
Dihydro-4-coumaroyl-CoA
Phloretin
2.4.1.357
Phlorizin
1.3.1.117
Desmethyl-xanthohumol
2.1.1.338
Xanthohumol
2.1.1.339
4'-O-Methyl-xanthohumol
2.4.1.185
Prunin
2.4.1.236
Naringin
Hesperetin
Hesperetin 7-O-glucoside
Neohesperidin
2.1.1.231
Isosakuranetin
Naringenin chalcone
Naringenin
Dihydrokaempferol
Leucopelargonidin
1.14.20.4
(-)-Epiafzelechin
1.3.1.77
Pelargonidin
1.17.1.3
(+)-Afzelechin
p-Coumaroyl-CoA
2.3.1.133
2.1.1.232
Sakuranetin
1.1.1.234
Apiforol
Naringenin chalcone 4'-O-glucoside
1.21.3.6
Aureusidin 6-O-glucoside
p-Coumaroyl shikimic acid
p-Coumaroyl quinic acid
8-C-Glucosylnaringenin
Vitexin
Kaempferol
Flavone and flavonol biosynthesis
1.14.14.81
2.4.1.-
Chalcone 2'-O-glucoside
Apigenin
1.14.13.-
1.14.14.96
Caffeoyl shikimic acid
Caffeoyl quinic acid
Quercetin
Bracteatin 6-O-glucoside
2',3,4,4',6'-Peptahydroxy-chalcone 4'-O-glucoside
Caffeoyl-CoA
2',3,4,4',6'-Pentahydroxychalcone
Eriodictyol
Dihydroquercetin
Leucocyanidin
Cyanidin
(-)-Epicatechin
Anthocyanin biosynthesis
Luteolin
(+)-Catechin
2.1.1.104
Luteoforol
Feruloyl-CoA
4,2',4',6'-Tetrahydroxy-3-methoxychalcone
Homoeriodictyol
Tricetin
Myricetin
Dihydrotricetin
Dihydromyricetin
Leucodelphinidin
Delphinidin
(-)-Epigallocatechin
(+)-Gallocatechin
00941 11/30/18
(c) Kanehisa Laboratories
DEG
Differentially Expressed Genes
4 9 21 39
Days Post Inoculation (dpi)
Over-expressed
Under-expressed
Over / Under-expressed
No differentially expressed

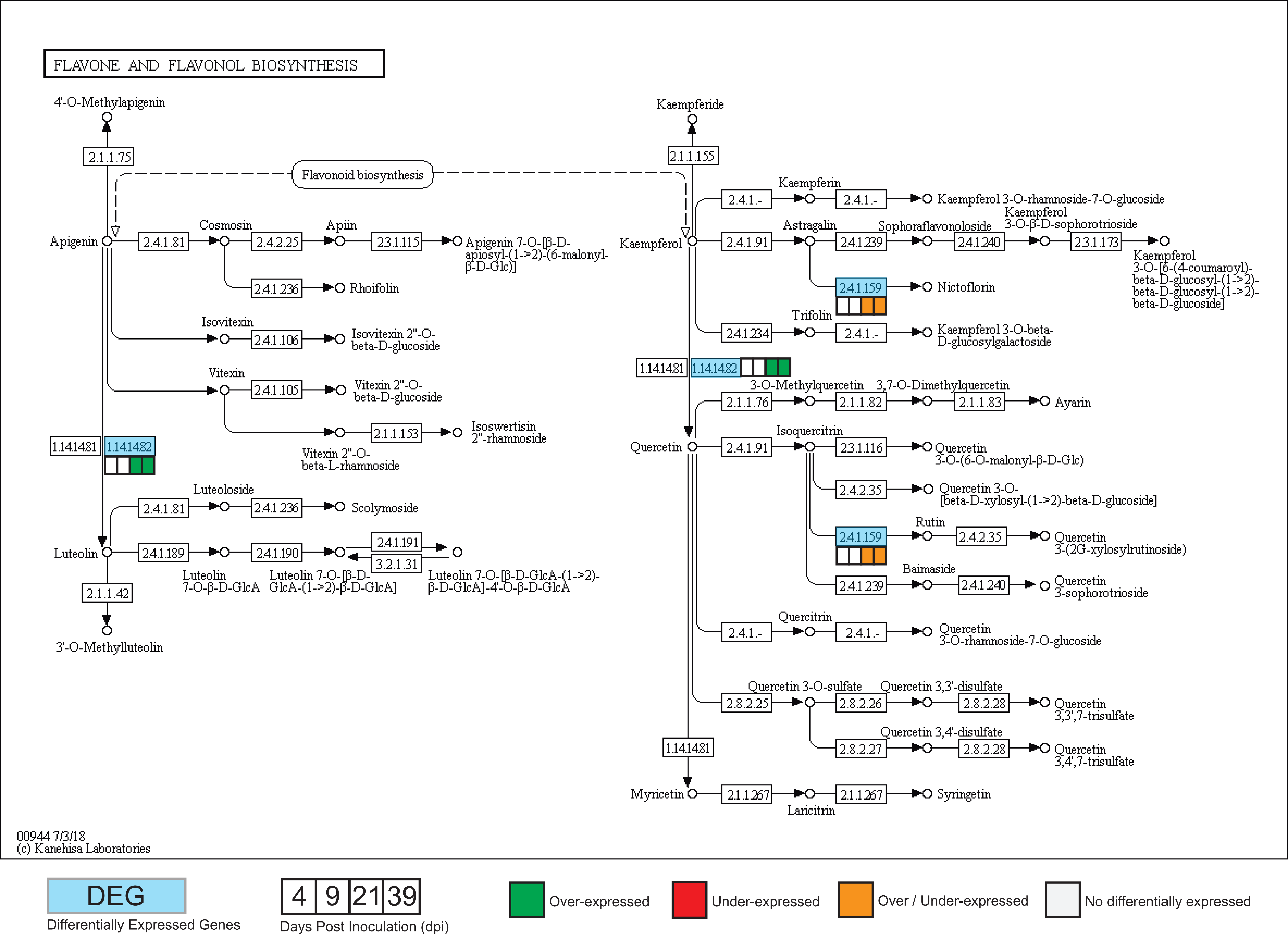
FLAVONE AND FLAVONOL BIOSYNTHESIS
4'-O-Methylapigenin
2.1.1.75
Flavonoid biosynthesis
Apigenin
2.4.1.81
Cosmosin
2.4.2.25
Apiin
2.3.1.115
Apigenin 7-O-[β-D-apiosyl-(1->2)-(6-malonyl-β-D-Glc)]
2.4.1.236
Rhoifolin
Isovitexin
2.4.1.106
Isovitexin 2''-O-beta-D-glucoside
Vitexin
2.4.1.105
Vitexin 2''-O-beta-D-glucoside
2.1.1.153
Isoswertisin 2''-rhamnoside
Vitexin 2''-O-beta-L-rhamnoside
1.14.14.81
1.14.14.82
Luteoloside
2.4.1.81
2.4.1.236
Scolymoside
Luteolin
2.4.1.189
2.4.1.190
2.4.1.191
3.2.1.31
Luteolin 7-O-β-D-GlcA
Luteolin 7-O-[β-D-GlcA-(1->2)-β-D-GlcA]
Luteolin 7-O-[β-D-GlcA-(1->2)-β-D-GlcA]-4'-O-β-D-GlcA
2.1.1.42
3'-O-Methylluteolin
Kaempferide
2.1.1.155
Kaempferol
Kaempferin
2.4.1.-
2.4.1.-
Kaempferol 3-O-rhamnoside-7-O-glucoside
Astragalin
2.4.1.91
2.4.1.239
Sophoraflavonoloside
2.4.1.240
Kaempferol 3-O-β-D-sophorotrioside
2.3.1.173
Kaempferol 3-O-[6-(4-coumaroyl)-beta-D-glucosyl-(1->2)-beta-D-glucosyl-(1->2)-beta-D-glucoside]
2.4.1.159
Nictoflorin
Trifolin
2.4.1.234
2.4.1.-
Kaempferol 3-O-beta-D-glucosylgalactoside
1.14.14.81
1.14.14.82
3-O-Methylquercetin
3,7-O-Dimethylquercetin
2.1.1.76
2.1.1.82
2.1.1.83
Ayarin
Quercetin
Isoquercitrin
2.4.1.91
2.3.1.116
Quercetin 3-O-(6-O-malonyl-β-D-Glc)
2.4.2.35
Quercetin 3-O-[beta-D-xylosyl-(1->2)-beta-D-glucoside]
Rutin
2.4.1.159
2.4.2.35
Quercetin 3-(2G-xylosylrutinoside)
Baimaside
2.4.1.239
2.4.1.240
Quercetin 3-sophorotrioside
Quercitrin
2.4.1.-
2.4.1.-
Quercetin 3-O-rhamnoside-7-O-glucoside
Quercetin 3-O-sulfate
Quercetin 3,3'-disulfate
2.8.2.25
2.8.2.26
2.8.2.28
Quercetin 3,3',7-trisulfate
Quercetin 3,4'-disulfate
2.8.2.27
2.8.2.28
Quercetin 3,4',7-trisulfate
1.14.14.81
Myricetin
2.1.1.267
Laricitrin
2.1.1.267
Syringetin
00944 7/3/18
(c) Kanehisa Laboratories
DEG
Differentially Expressed Genes
4 9 21 39
Days Post Inoculation (dpi)
Over-expressed
Under-expressed
Over / Under-expressed
No differentially expressed

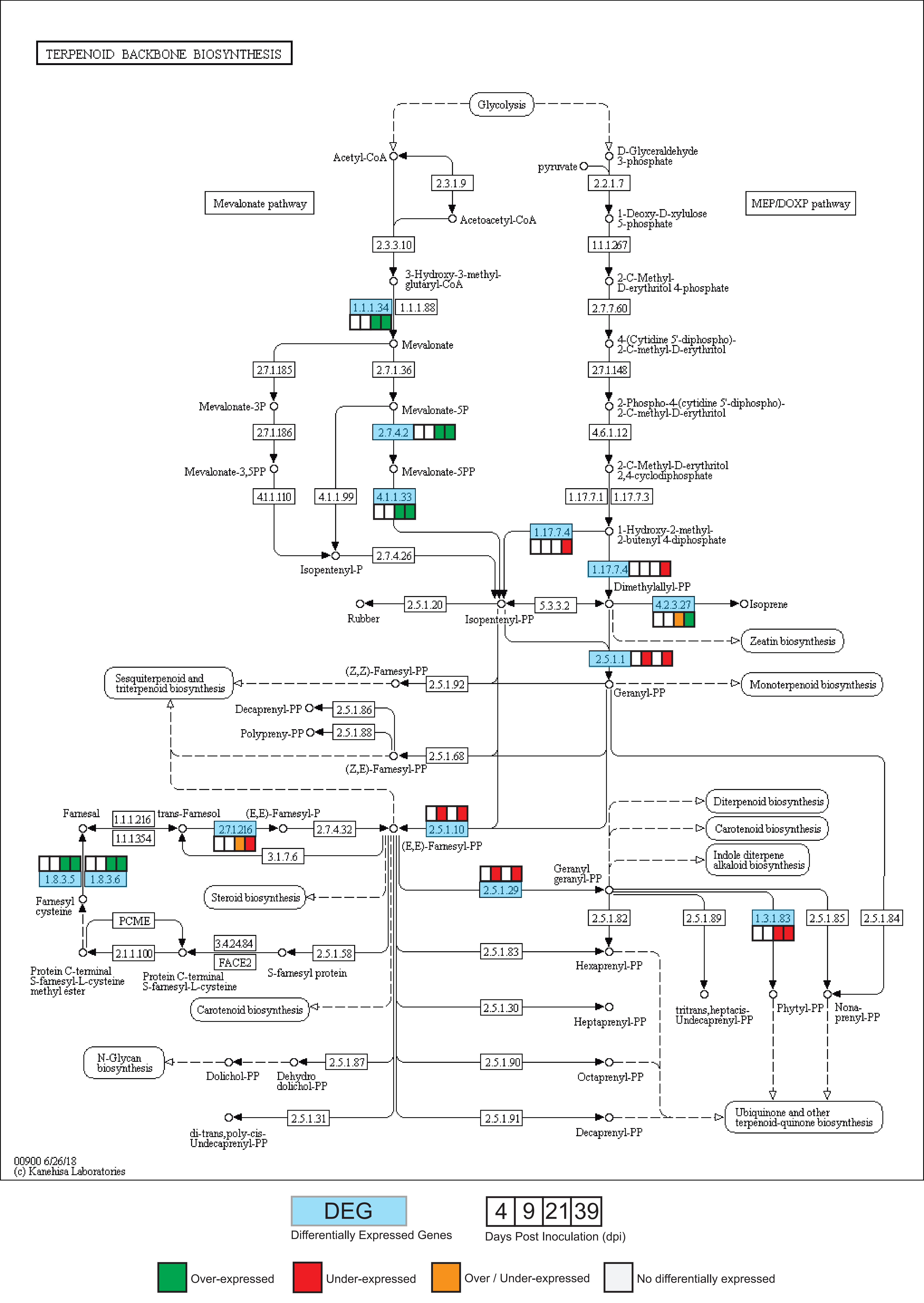
TERPENOID BACKBONE BIOSYNTHESIS
Glycolysis
Acetyl-CoA
2.3.1.9
Acetoacetyl-CoA
2.3.3.10
3-Hydroxy-3-methyl-glutaryl-CoA
1.1.1.34
1.1.1.88
Mevalonate
2.7.1.185
2.7.1.36
Mevalonate-3P
Mevalonate-5P
2.7.1.186
2.7.4.2
Mevalonate-3,5PP
Mevalonate-5PP
4.1.1.110
4.1.1.99
4.1.1.33
Isopentenyl-P
2.7.4.26
Mevalonate pathway
D-Glyceraldehyde 3-phosphate
pyruvate
2.2.1.7
1-Deoxy-D-xylulose 5-phosphate
MEP/DOXP pathway
1.1.1.267
2-C-Methyl-D-erythritol 4-phosphate
2.7.7.60
4-(Cytidine 5'-diphospho)-2-C-methyl-D-erythritol
2.7.1.148
2-Phospho-4-(cytidine 5'-diphospho)-2-C-methyl-D-erythritol
4.6.1.12
2-C-Methyl-D-erythritol 2,4-cyclodiphosphate
1.17.7.1
1.17.7.3
1.17.7.4
1-Hydroxy-2-methyl-2-butenyl 4-diphosphate
Dimethylallyl-PP
Rubber
2.5.1.20
Isopentenyl-PP
5.3.3.2
4.2.3.27
Isoprene
Zeatin biosynthesis
2.5.1.1
Sesquiterpenoid and triterpenoid biosynthesis
(Z,Z)-Farnesyl-PP
2.5.1.92
Geranyl-PP
Monoterpenoid biosynthesis
Decaprenyl-PP
2.5.1.86
Polypreny-PP
2.5.1.88
2.5.1.68
(Z,E)-Farnesyl-PP
Diterpenoid biosynthesis
Carotenoid biosynthesis
Indole diterpene alkaloid biosynthesis
Farnesal
1.1.1.216
trans-Farnesol
(E,E)-Farnesyl-P
1.1.1.354
2.7.1.216
2.7.4.32
2.5.1.10
(E,E)-Farnesyl-PP
3.1.7.6
1.8.3.5
1.8.3.6
Farnesyl cysteine
Steroid biosynthesis
2.5.1.29
Geranyl geranyl-PP
PCME
3.4.24.84
FACE2
2.1.1.100
2.5.1.58
Protein C-terminal S-farnesyl-L-cysteine methyl ester
Protein C-terminal S-farnesyl-L-cysteine
S-farnesyl protein
2.5.1.82
2.5.1.89
1.3.1.83
2.5.1.85
2.5.1.84
2.5.1.83
Hexaprenyl-PP
Carotenoid biosynthesis
2.5.1.30
Heptaprenyl-PP
tritrans,heptacis-Undecaprenyl-PP
Phytyl-PP
Nona-prenyl-PP
N-Glycan biosynthesis
Dolichol-PP
Dehydro dolichol-PP
2.5.1.87
2.5.1.90
Octaprenyl-PP
Ubiquinone and other terpenoid-quinone biosynthesis
2.5.1.31
di-trans,poly-cis-Undecaprenyl-PP
2.5.1.91
Decaprenyl-PP
00900 6/26/18
(c) Kanehisa Laboratories
DEG
Differentially Expressed Genes
4
9
21
39
Days Post Inoculation (dpi)
Over-expressed
Under-expressed
Over / Under-expressed
No differentially expressed

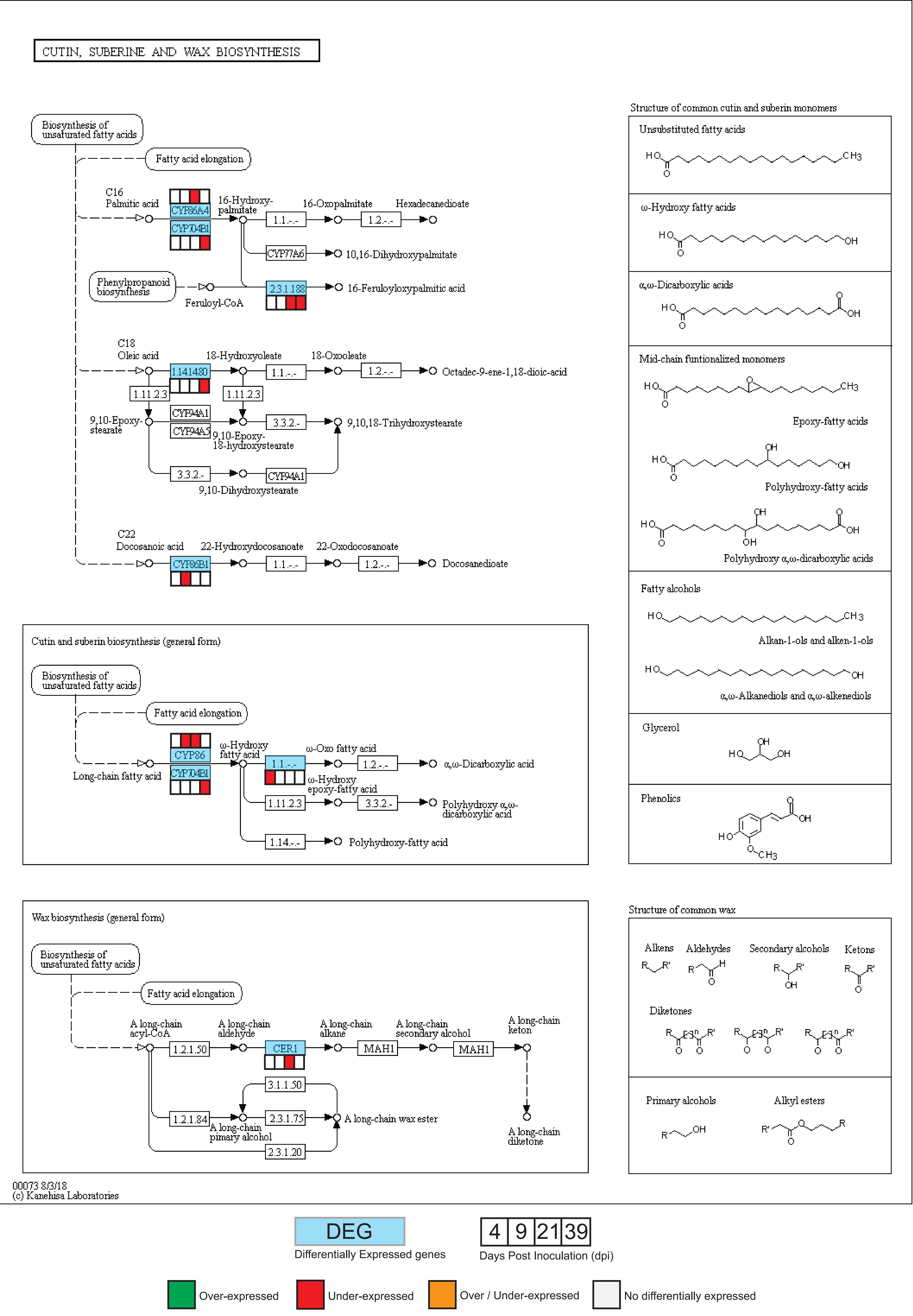
CUTIN, SUBERINE AND WAX BIOSYNTHESIS
Biosynthesis of unsaturated fatty acids
Fatty acid elongation
C16
Palmitic acid
CYP86A4
CYP704B1
16-Hydroxy-palmitate
16-Oxopalmitate
Hexadecanedioate
1.1.-.-
1.2.-.-
CYP77A6
10,16-Dihydroxypalmitate
Phenylpropanoid biosynthesis
Feruloyl-CoA
2.3.1.188
16-Feruloyloxypalmitic acid
C18
Oleic acid
1.14.14.80
18-Hydroxyoleate
18-Oxooleate
Octadec-9-ene-1,18-dioic-acid
1.11.2.3
9,10-Epoxy-stearate
CYP94A1
CYP94A5
9,10-Epoxy-18-hydroxystearate
3.3.2.-
9,10,18-Trihydroxystearate
9,10-Dihydroxystearate
C22
Docosanoic acid
22-Hydroxydocosanoate
22-Oxodocosanoate
CYP86B1
Docosanedioate
Cutin and suberin biosynthesis (general form)
CYP86
ω-Hydroxy fatty acid
ω-Oxo fatty acid
α,ω-Dicarboxylic acid
Long-chain fatty acid
ω-Hydroxy epoxy-fatty acid
Polyhydroxy α,ω-dicarboxylic acid
1.14.-.-
Polyhydroxy-fatty acid
Wax biosynthesis (general form)
A long-chain acyl-CoA
A long-chain aldehyde
A long-chain alkane
A long-chain secondary alcohol
A long-chain keton
1.2.1.50
CER1
MAH1
3.1.1.50
1.2.1.84
2.3.1.75
A long-chain wax ester
A long-chain pimary alcohol
2.3.1.20
A long-chain diketone
00073 8/3/18
(c) Kanehisa Laboratories
Structure of common cutin and suberin monomers
Unsubstituted fatty acids
ω-Hydroxy fatty acids
α,ω-Dicarboxylic acids
Mid-chain funtionalized monomers
Epoxy-fatty acids
Polyhydroxy-fatty acids
Polyhydroxy α,ω-dicarboxylic acids
Fatty alcohols
Alkan-1-ols and alken-1-ols
α,ω-Alkanediols and α,ω-alkenediols
Glycerol
Phenolics
Structure of common wax
Alkens
Aldehydes
Secondary alcohols
Ketons
Diketones
Primary alcohols
Alkyl esters
DEG
Differentially Expressed genes
4 9 21 39
Days Post Inoculation (dpi)
Over-expressed
Under-expressed
Over / Under-expressed
No differentially expressed
