# Supplementary material for: RNA-Seq and secondary metabolite analyses reveal a putative defence-transcriptome in Norway spruce (Picea abies) against needle bladder rust (Chrysomyxa rhododendri) infection
Source: BMC Genomics. 2020 May 1;21:336. doi: 10.1186/s12864-020-6587-z (PMC7195740; doi:10.1186/s12864-020-6587-z)
Supplement: Supplementary file 12 — Additional file 12: Command S1. STAR command. The detailed STAR settings used for mapping and genome index generation. [file 12864_2020_6587_MOESM12_ESM.doc]

STAR --runMode genomeGenerate --runThreadN 12 --genomeDir star_index2/ --genomeFastaFiles Pabies01-genome-collapsed3.fa ../ERCC/ERCC92.fa ../SIRV/SIRV_170504a.fasta --sjdbGTFfile Pabies01b_ERCC_SIRV.gtf --sjdbOverhang 100 --genomeSAindexNbases 10 --genomeSAsparseD 5 --limitGenomeGenerateRAM 99000000000
