# Supplementary material for: RNA-Seq and secondary metabolite analyses reveal a putative defence-transcriptome in Norway spruce (Picea abies) against needle bladder rust (Chrysomyxa rhododendri) infection
Source: BMC Genomics. 2020 May 1;21:336. doi: 10.1186/s12864-020-6587-z (PMC7195740; doi:10.1186/s12864-020-6587-z)
Supplement: Supplementary file 15 — Additional file 15: Figure S6. RT-qPCR assay details. Primer sequences of RT-qPCR assays. [file 12864_2020_6587_MOESM15_ESM.pdf]

RT-qPCR assay details

Primer for candidate reference genes

Gene ID*	Gene symbol	Name	Oligo sequence (5' – 3')	Amplicon size (bp)	*E*	R^2	Reference
MA_10427661g0030	ACT	actin	F: TGAGCTCCCTGATGGGCAGGTGA<br>R: TGGATACCAGCAGCTTCCATCCCAAT	104	0.9	0.998	Yakovlev et al. 2006
MA_93486g0010	ATUB	tubulin α-3 chain	F: GGCATACCGGCAGCTCTTC<br>R: AAGTTGTTGGCGGCGTCTT	66	0.99	0.996	Yakovlev et al. 2006
N/A	CAC	clathrin adaptor complex subunit	F: ACGGAATAATGATGTGCTAA<br>R: CCCTCTTGTGTGATGTAAA	108	n.d.**	n.d.**	de Vega-Bartol et al. 2013
MA_434977g0010	EF1a	elongation factor 1-α	F: GGATTGCCACACTTGCCACA<br>R: CTTGGGTTCCTTCTCCAGTTCC	94	0.91	0.999	Yakovlev et al. 2014
MA_130835g0010	GAPDH	glyceraldehyde-3-phosphate dehydrogenase	F: TGCTGCAAAGGCAGTTGGGAAGG<br>R: GGCGACATGTCAGATCCACGACTG	110	0.92	0.994	Yakovlev et al. 2006
MA_14427g0010	pUBQ	polyubiquitin	F: TGGTCGTACTCTGGCCGATTATA<br>R: ACACCTAGCGGCACACAGTTAA	97	0.9	0.998	Yakovlev et al. 2006
MA_8884419g0010, MA_10435699g0020	UBI	ubiquitin	F: GTTGATTTTTGCTGGCAAGC<br>R: CACCTCTCAGACGAAGTAC	101	0.91	0.997	Schmidt and Gershenzon, 2007

*ConGenIE database (http://congenie.org/); F and R: forward and reverse primer; *E*: amplification efficiency; R^2: correlation coefficient of standard curve; **not determined (Cq values out of quantitative range or not detected)

RefFinder (Xie et al. 2012) ranking of candidate reference genes

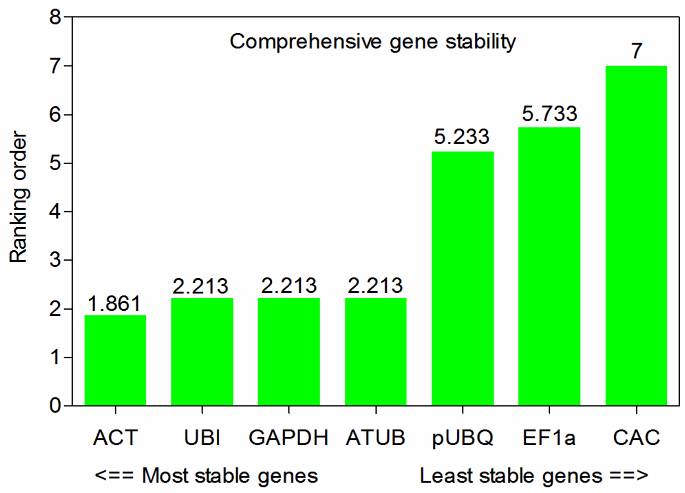

Primer for genes of interest

Gene ID*	Gene symbol	Name	Oligo sequence (5' – 3')	Amplicon size (bp)	*E*	R^2
MA_10204459g0010	CALM	calmodulin	F: GCGTTGAAGGAGGCATTTA<br>R: CGGTGATGGAGGAGAAGAA	91	0.999	0.994
MA_10280648g0010	CALM	calmodulin	F: GCTGAAGGAGGCGTTTAAT<br>R: CGATGAAGGAGGAGGAGAA	89	0.899	0.999
MA_52889g0010	CALM/CML	calmodulin / calcium-binding protein CML	F: AAATTGCGGAGTTCAGAGAG<br>R: CCAGCTCCTTGGTTGTTATG	75	0.963	0.980
MA_10313114g0010	CHIB	basic endochitinase B	F: CCACGGACGAGGACCTATT<br>R: CGGGTTGTTTATGAGATCGTATCC	88	0.971	0.990
MA_8921185g0010	CHIB	basic endochitinase B	F: CCGCATCGGGTTCTACAAA<br>R: GGCCTCTGGTTGTTACAGTC	81	1.07	0.992
MA_5735g0010	CHS	chalcone synthase	F: CTGCCCAGACAATTCTTCC<br>R: GTAAGTCCGACCTCTCTCAA	67	0.999	0.990
MA_10431324g0020	CML	calcium-binding protein CML	F: TCATACTTGGGACCTCTCTC<br>R: CAGTAACCCTTGTACGTCTTC	88	0.999	0.993
MA_115550g0010	CPK	calcium-dependent protein kinase	F: TCGTGGAGGAGAGACATTTA<br>R: CATCAGCTTCAGGACCATAAT	92	0.848	0.994
MA_9458g0010	CPK	calcium-dependent protein kinase	F: GACTAACAGCTCAGCAAGTG<br>R: CCCAAGAGGAACATTAGGAAC	74	1	0.994
MA_35694g0010	CTR1	serine/threonine-protein kinase CTR1	F: GGATTGGAGCAGGTTCTTT<br>R: CTTCGTGGAAGTCTTGTTCTAT	96	0.985	0.993
MA_76780g0010	CYP75B1	flavonoid 3'-monooxygenase	F: GCAACCCACAGATGATGAA<br>R: GACTCCTTTAGCGTCCTTTG	79	0.981	0.958
MA_10431201g0020	EDS1	enhanced disease susceptibility 1 protein	F: AAGAGGATGTAACAAACATGGA<br>R: CGATCACGCACATAATGAAAC	87	0.918	0.994
MA_101621g0020	EDS1	enhanced disease susceptibility 1 protein	F: AAGTTGATACGGAATCCTCTTAC<br>R: TTCAATTTCTCCTGCTACTTGA	101	0.999	0.997
MA_20516g0010	EIN3	ethylene-insensitive protein 3	F: CAAGAGCAAGCGAGAAGAA<br>R: GAGCCTTACAAACCTCCATC	88	0.988	0.990
MA_10434030g0010	HSP90A	molecular chaperone HtpG	F: TAGCCTCATCATCAACACTTT<br>R: TTTATCCGTTAGCCCTTCG	112	1	0.996
MA_12191g0010	HSP90A	molecular chaperone HtpG	F: GTCTGATCTCGTGAACAACTT<br>R: CAAGTGCTTCCATAAACTCTTTG	68	1.05	0.999
MA_10431770g0010	HSP90B	heat shock protein 90kDa beta	F: CTTCGGGAGCTTGTTAGTAATG<br>R: TCTCTAAGTCACCACCATCTC	100	1.06	0.998
MA_10431031g0010	HSP90B	heat shock protein 90kDa beta	F: CGACACCAAGCTCGAAAT<br>R: CCACGATCTCGGATTGAAAG	69	1.05	0.998
MA_10194g0020	MKK9	mitogen-activated kinase kinase 9	F: CCTGATGGGTATGGTGGAAAG<br>R: GCGCACACTCCAGTAAAGATA	79	1.05	0.999
MA_10437020g0010	MPK6	mitogen-activated protein kinase 6	F: TCCCTCCGCTATTTCCTATT<br>R: GCAACTTGCTCATTGGTTTC	82	1	0.988
MA_10435905g0020	MYC2	transcription factor MYC2	F: TGGCCAGAGGAAACTTTGAG<br>R: CAGAGTTCAGGGACTGGATTTC	100	0.856	0.991
MA_53673g0010	PR1	pathogenesis-related protein 1	F: CGTGATGGCTTGGGTAAAC<br>R: TCTCCACACCACTTGAGTATAG	100	0.999	0.986
MA_133206g0010	SGT1	suppressor of G2 allele of SKP1	F: CCTCGGCGAACATTAAACTC<br>R: GGTATGCCTTTGCCAGATTAG	93	1.12	0.991

*ConGenIE database (http://congenie.org/); F and R: forward and reverse primer; *E*: amplification efficiency; R^2: correlation coefficient of standard curve

References:

de Vega-Bartol JJ, Santos RR, Simões M, Miguel CM: Normalizing gene expression by quantitative PCR during somatic embryogenesis in two representative conifer species: Pinus pinaster and Picea abies. Plant Cell Reports 2013;32(5);715-729.

Schmidt A, Gershenzon J: Cloning and characterization of two different types of geranyl diphosphate synthases from Norway spruce (Picea abies). Phytochemistry 2008;69;49-57.

Yakovlev IA, Fossdal C-G, Johnsen Ø, Junttila O, Skrøppa T: Analysis of gene expression during bud burst initiation in Norway spruce via ESTs from subtracted cDNA libraries. Tree Genetics and Genomes 2006;2;39-52.

Yakovlev IA, Lee Y, Rotter B, Olsen JE, Skrøppa T, Johnsen Ø, Fossdal CG: Temperature-dependent differential transcriptomes during formation of an epigenetic memory in Norway spruce embryogenesis. Tree Genetics and Genomes 2014;10(2);355-366.

Xie F, Xiao P, Chen D, Xu L, Zhang B: miRDeepFinder: a miRNA analysis tool for deep sequencing of plant small RNAs. Plant Mol Biol. 2012;80:75-84.
